# Supplementary material for: In Silico Evaluation of CRISPR-Based Assays for Effective Detection of SARS-CoV-2
Source: Pathogens. 2022 Aug 25;11(9):968. doi: 10.3390/pathogens11090968 (PMC9506389; doi:10.3390/pathogens11090968)

Figure S1: Multiple sequence alignments of SARS-CoV-2 VOC (Alpha, Beta, Gamma, Delta and Omicron strains). The black highlight represented the binding sites of primers or crRNAs obtained from each assay. The dots represent the conserve sequences whereas the bases (A T, C or G) represent the mutations occurred in the VOC.

Title: A CRISPR-based and post-amplification coupled SARS-CoV-2 detection with a portable evanescent wave biosensor

Target: S gene
Oligo: SARS-CoV-2 gene S crRNA (anti-sense)

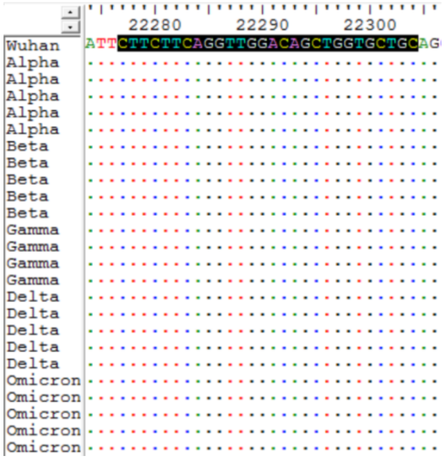

Target: N gene
Oligo: SARS-CoV-2 gene N crRNA (anti-sense)

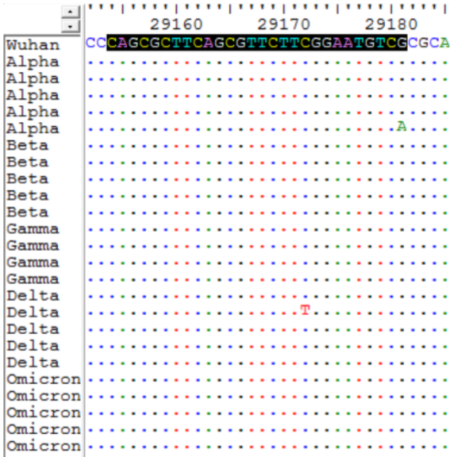

Target: Orf1ab gene
Oligo: Universal crRNA Orf1ab (anti-sense)

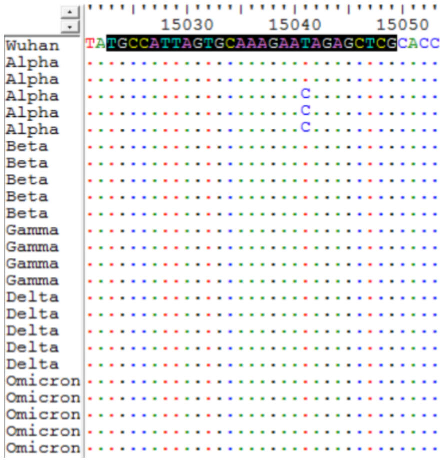

Title: A Novel Miniature CRISPR-Cas13 System for SARS-CoV Diagnostics

Target: N gene
Oligo: SC-F3

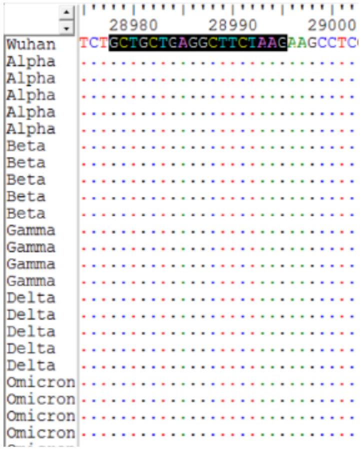

Target: N gene
Oligo: SC-B3 (anti-sense)

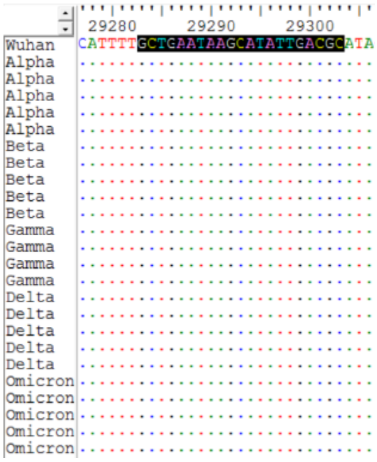

Target: N gene
Oligo: SC-FIP

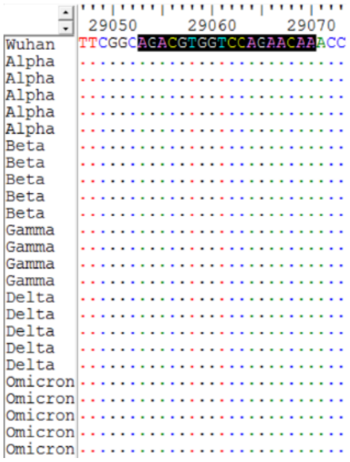

Target: N gene
Oligo: SC-BIP (anti-sense)

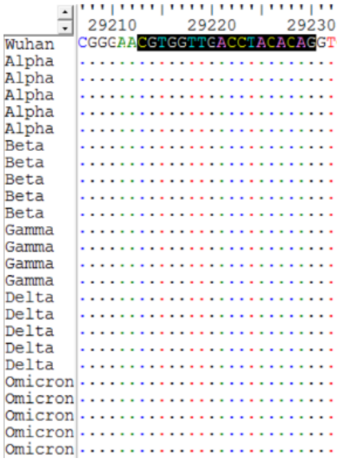

Target: N gene
Oligo: SC-LF (anti-sense)

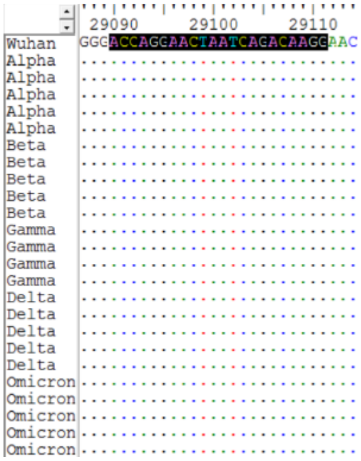

Target: N gene
Oligo: SC-LB

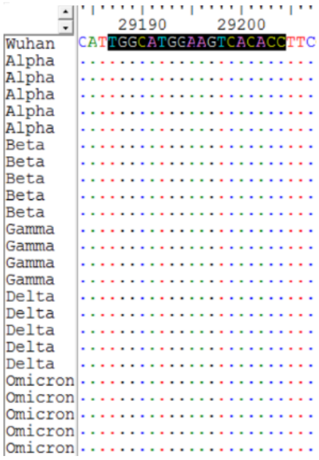

Target: N gene

Oligo: crRNA 4

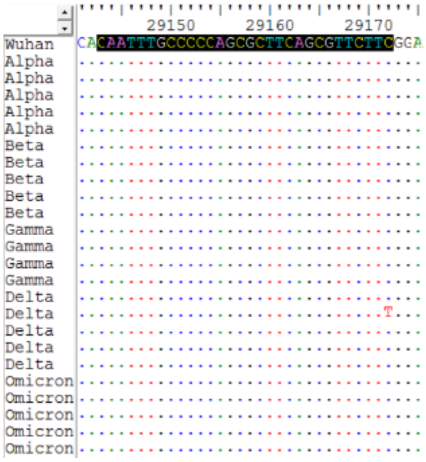

Title: A one-step, one-pot CRISPR nucleic acid detection platform (CRISPR-top): Application for the diagnosis of COVID-19

Target: Orf1ab
Oligo: ORF1ab-F3

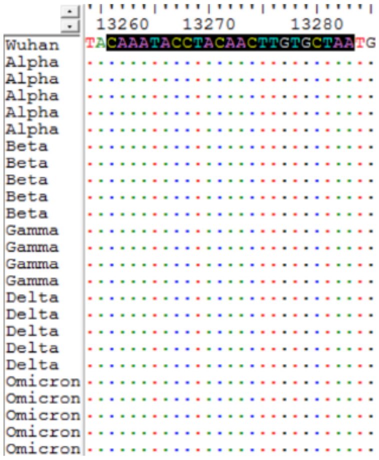

Target: Orf1ab
Oligo: ORF1ab-B3 (anti-sense)

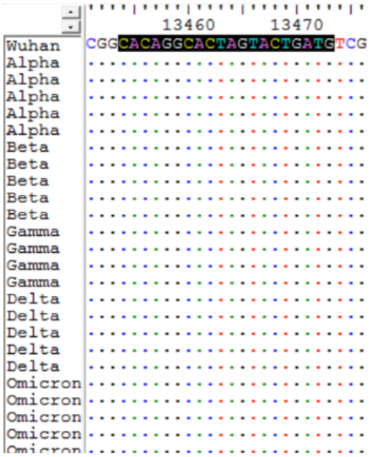

Target: Orf1ab
Oligo: ORF1ab-FIP

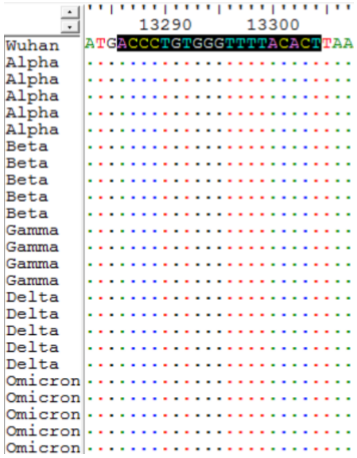

Target: Orf1ab
Oligo: ORF1ab-BIP (anti-sense)

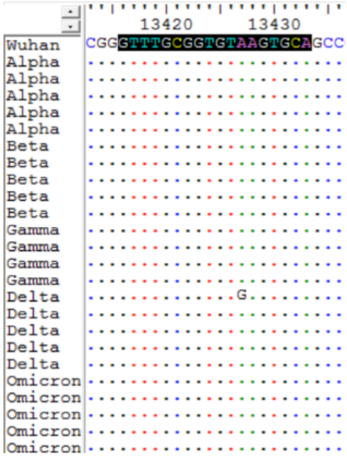

Target: Orf1ab
Oligo: ORF1ab-LF (anti-sense)

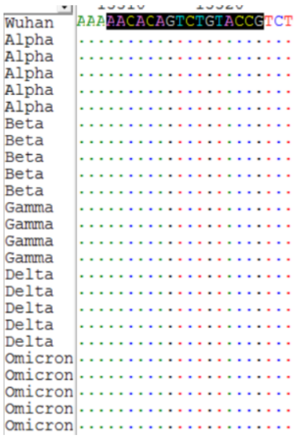

Target: Orf1ab
Oligo: ORF1ab-LB

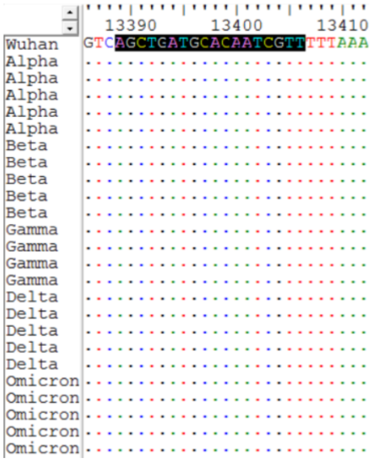

Target: N gene
Oligo: NP-F3

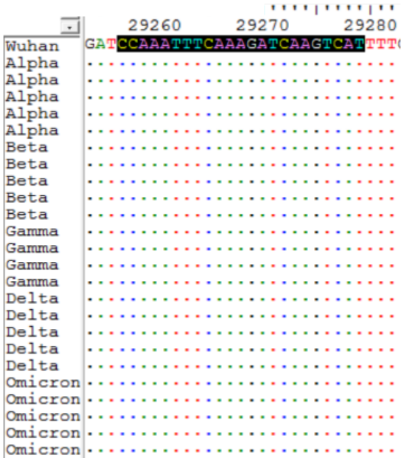

Target: N gene
Oligo: NP-B3 (anti-sense)

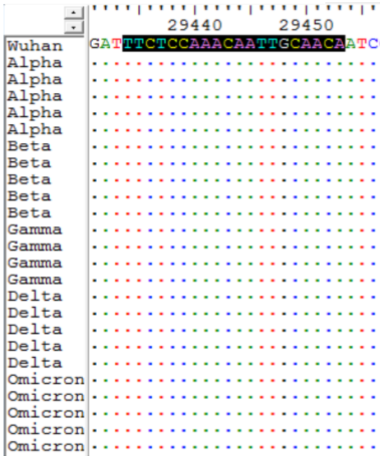

Target: N gene
Oligo: NP-FIP

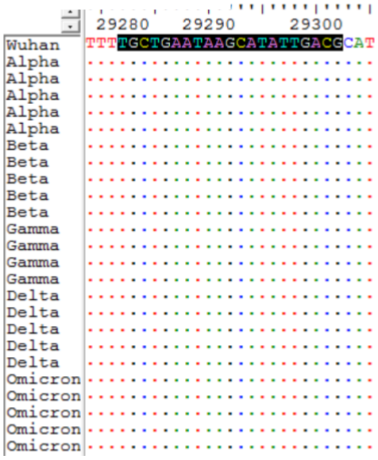

Target: N gene
Oligo: NP-BIP (anti-sense)

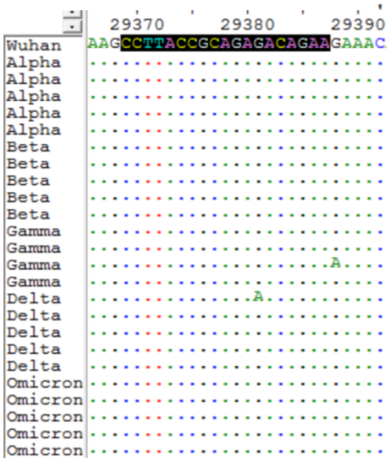

Target: N gene
Oligo: NP-LF (anti-sense)

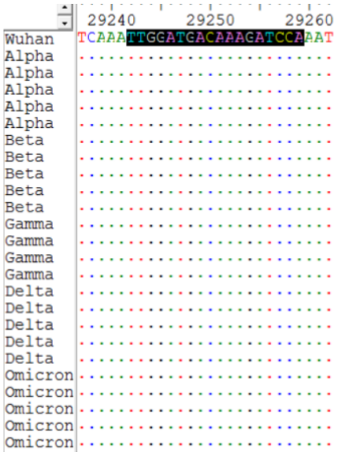

Target: N gene
Oligo: NP-LB

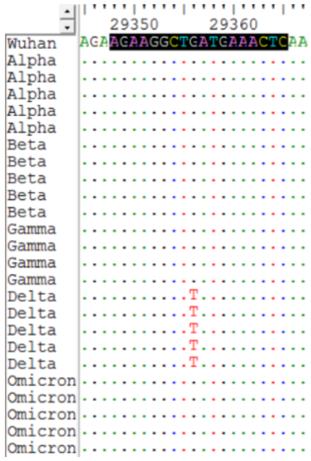

Target: Orf1ab
Oligo: ORF1ab-gRNA

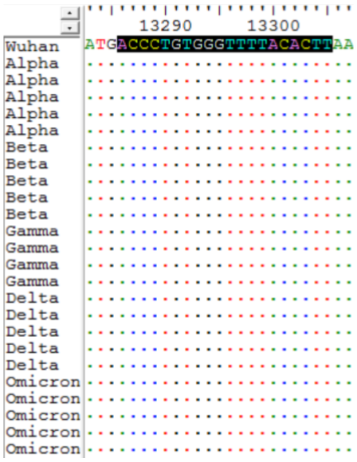

Target: Orf1ab
Oligo: NP-gRNA

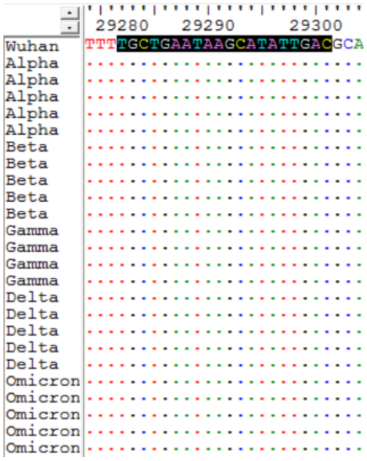

Title: A Saliva-Based RNA Extraction-Free Workflow Integrated With Cas13a for SARS-CoV-2 Detection

Target: S gene
Oligo: T7-S-FP

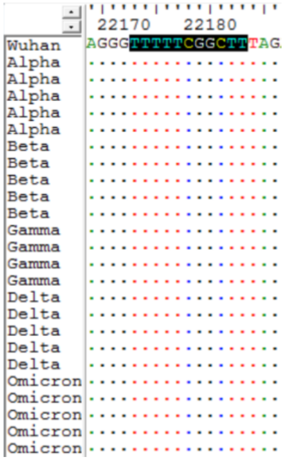

Target: S gene
Oligo: T7-S-RP (anti-sense)

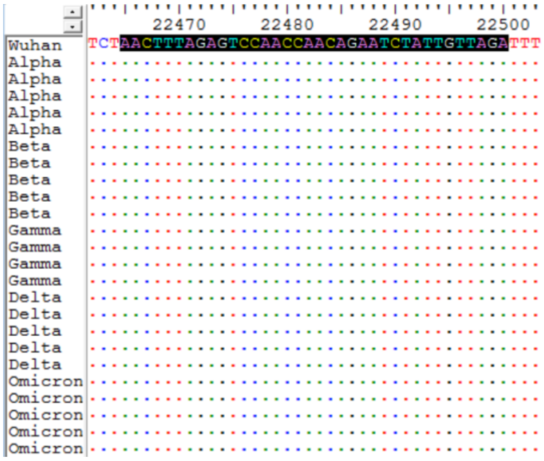

Target: Orf1ab
Oligo: T7-Orf1ab-FP

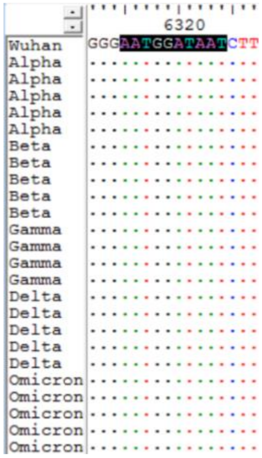

Target: Orf1ab
Oligo: T7-Orf1ab-RP (anti-sense)

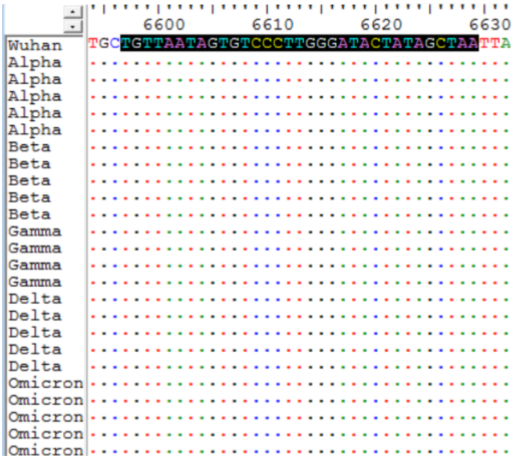

Target: S gene
Oligo: S gene crRNA (anti-sense)

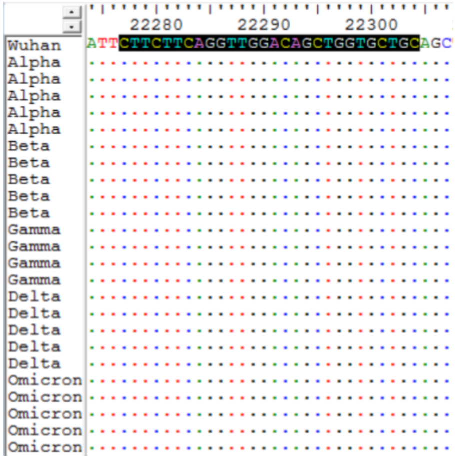

Target: Orf1ab
Oligo: Orf1ab crRNA (anti-sense)

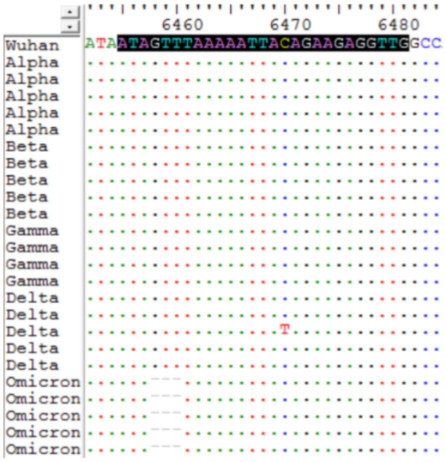

Title: A Scalable, Easy-to-Deploy Protocol for Cas13-Based Detection of SARS-CoV-2 Genetic Material

Target: N gene
Oligo: N1 fwd

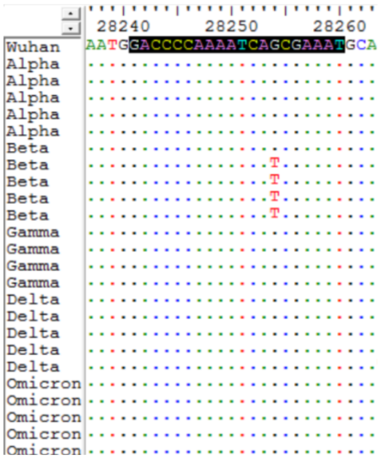

Target: N gene
Oligo: N1 rev (anti-sense)

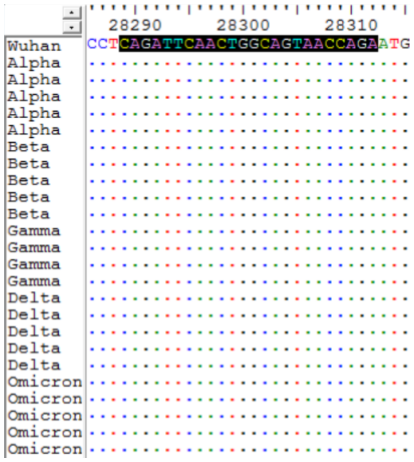

Target: N gene
Oligo: N2 fwd

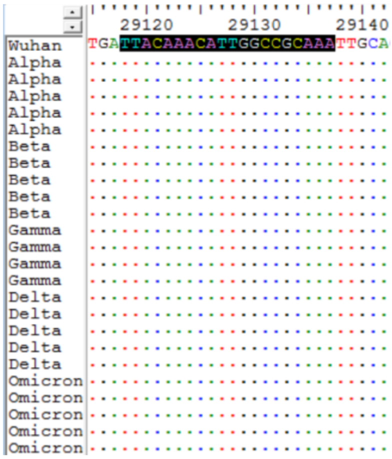

Target: N gene
Oligo: N2 rev (anti-sense)

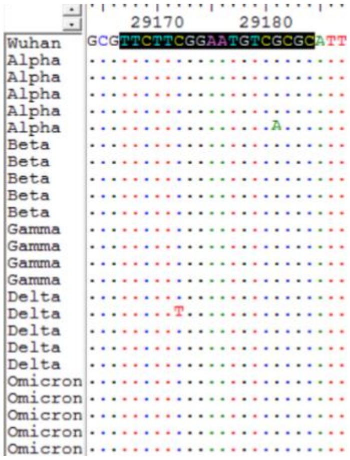

Target: N gene
Oligo: N3 fwd

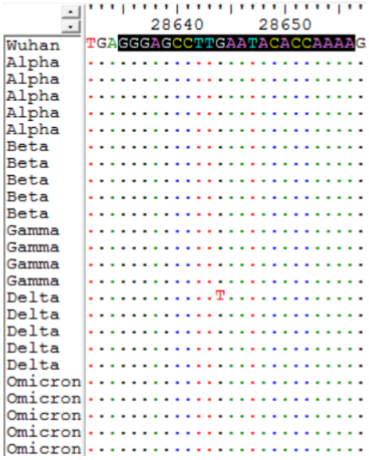

Target: N gene
Oligo: N3 rev (anti-sense)

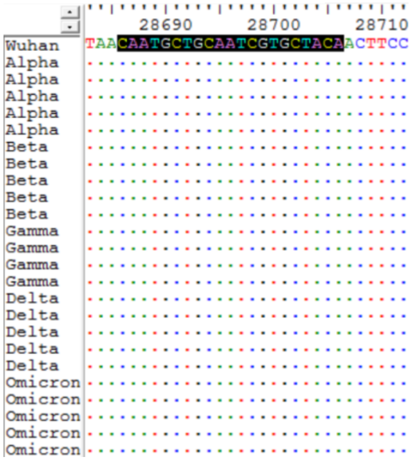

Title: A smartphone-based visual biosensor for CRISPR-Cas powered SARS-CoV-2 diagnostics

Target: N gene

Oligo: PCR Forward primer Target: N gene

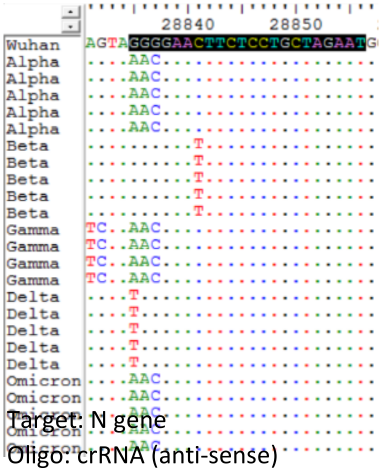

Target: N gene

Oligo: crRNA (anti-sense)

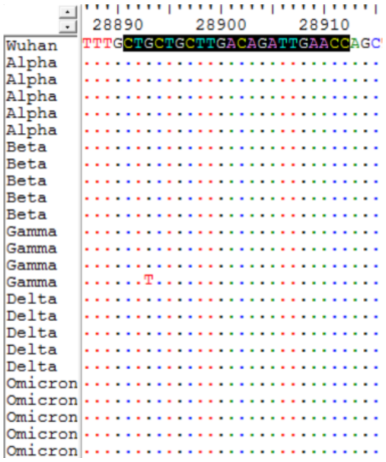

Target: N gene

Oligo: PCR Reverse primer (anti-sense)

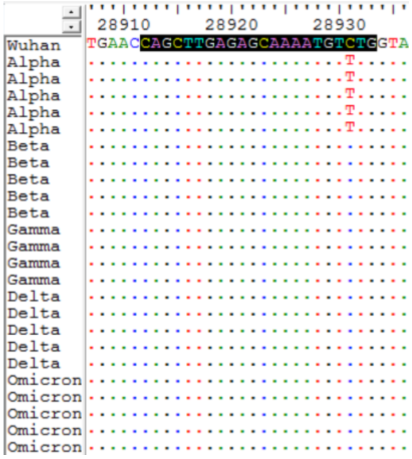

Title: A smartphone-read ultrasensitive and quantitative saliva test for COVID-19

Target: ORF1ab
Oligo: ORF1ab-F

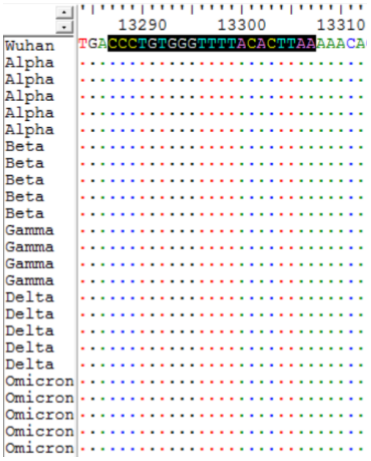

Target: ORF1ab
Oligo: ORF1ab-R (anti-sense)

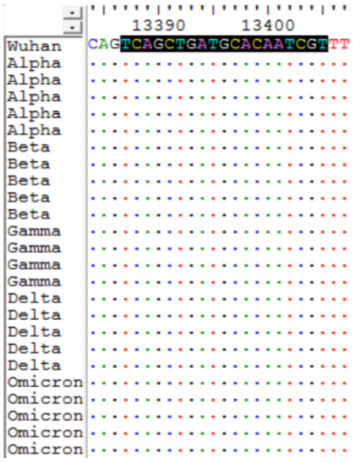

Target: N gene
Oligo: N Forward Primer

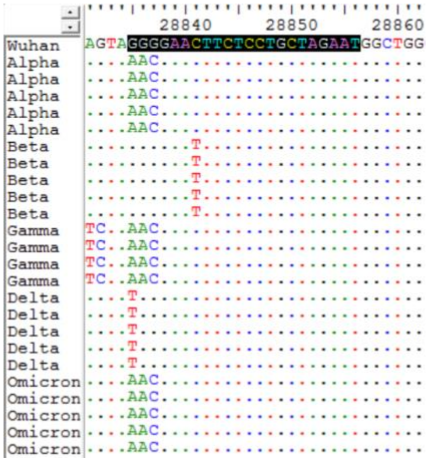

Target: N gene
Oligo: N Reverse Primer (anti-sense)

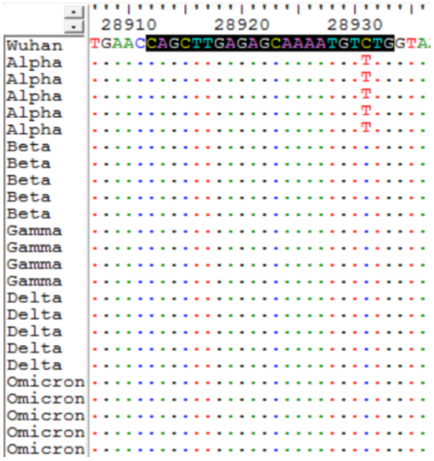

Target: ORF1ab
Oligo: gRNA -ORF1ab (anti-sense)

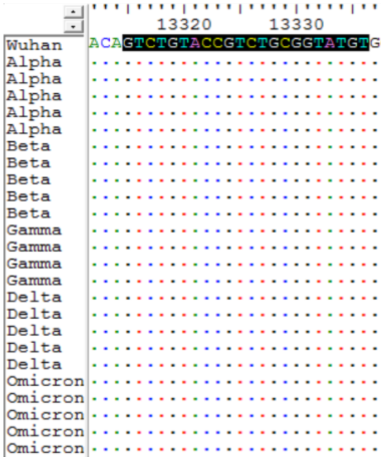

Target: N gene
Oligo: N gRNA (sense)

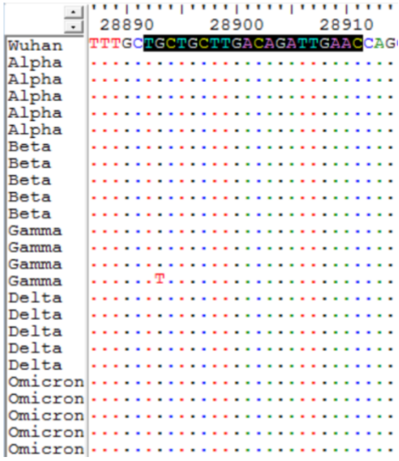

Title: A Thermostable Cas12b from Brevibacillus Leverages One-pot Detection of SARS-CoV-2 Variants of Concern

Target: N gene
Oligo: N_F3

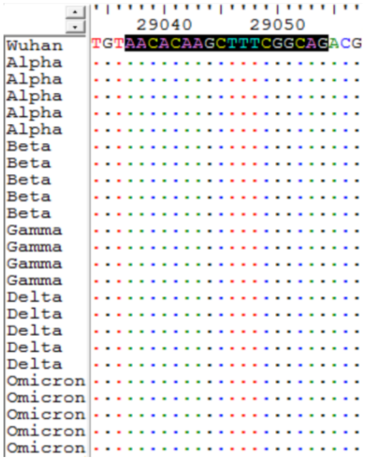

Target: N gene
Oligo: N_B3 (anti-sense)

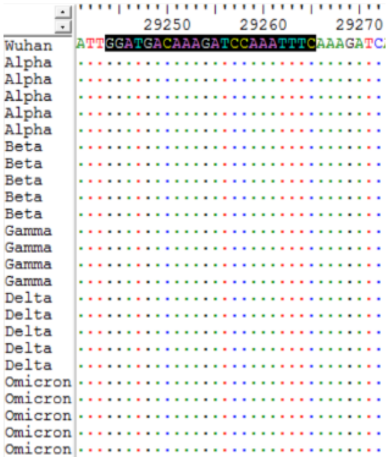

Target: N gene
Oligo: N_FIP

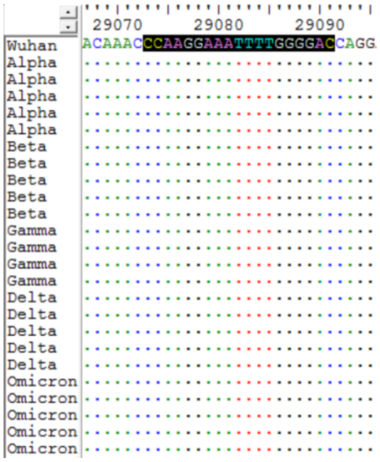

Target: N gene
Oligo: N_BIP (anti-sense)

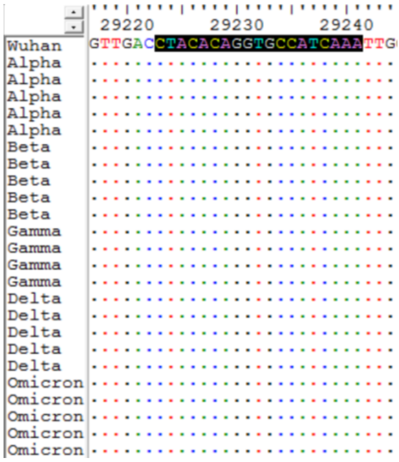

Target: N gene
Oligo: N_LF (anti-sense)

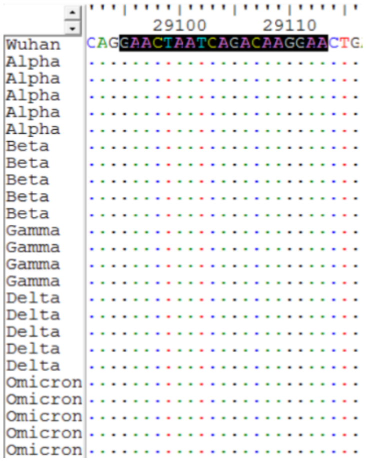

Target: N gene
Oligo: N_LB

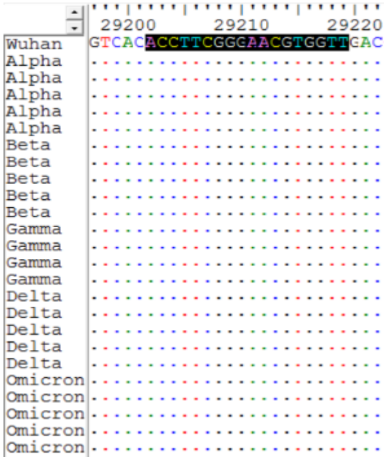

Target: N gene

Oligo: N gene crRNA for presence of SARS-CoV-2 (anti-sense)

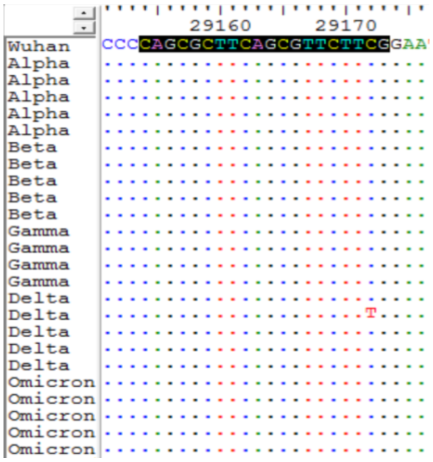

Title: Amplification-free detection of SARS-CoV-2 with CRISPR-Cas13a and mobile phone microscopy

Target: N gene
Oligo: SARS-CoV-2 crRNA 2 (anti-sense)

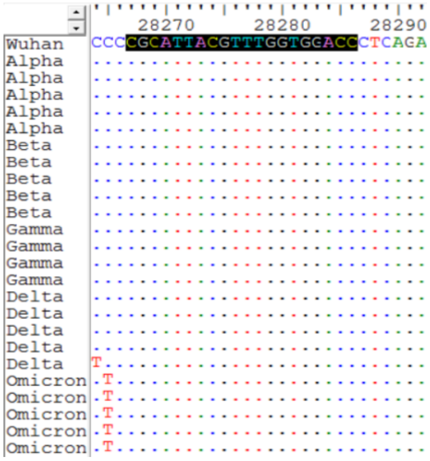

Target: N gene
Oligo: SARS-CoV-2 crRNA 4 (anti-sense)

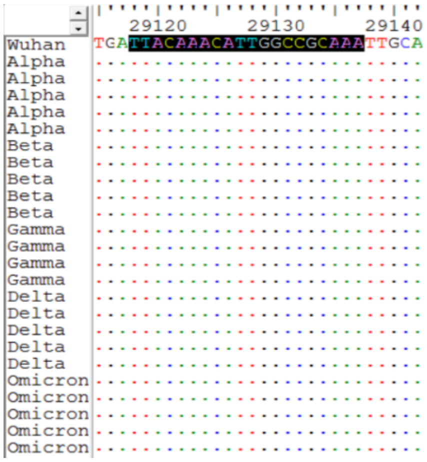

Target: N gene
Oligo: SARS-CoV-2 crRNA 21 (anti-sense)

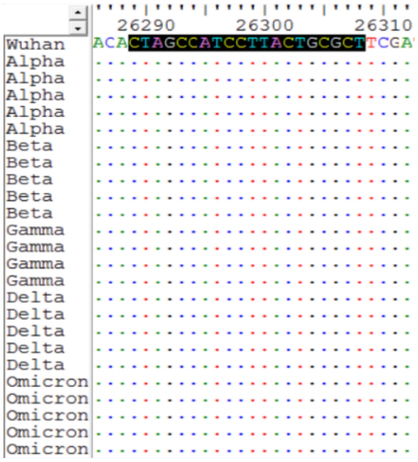

Title: An engineered CRISPR-Cas12a variant and DNA-RNA hybrid guides enable robust and rapid COVID-19 testing

Target: S gene
Oligo: S2 F3

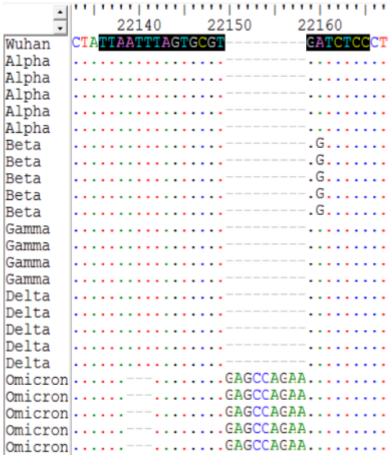

Target: S gene
Oligo: S2 B3 (anti-sense)

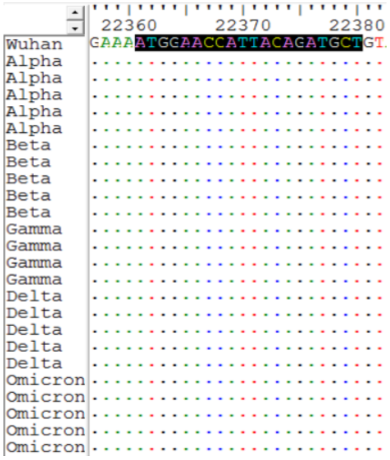

Target: S gene
Oligo: S2 FIP (set C)

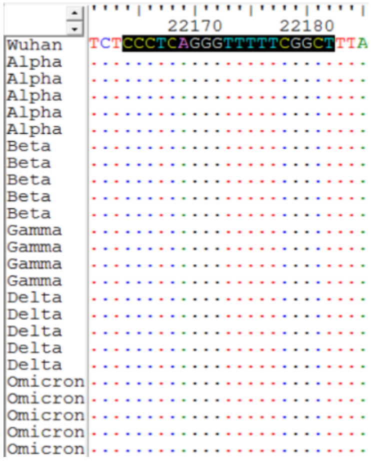

Target: S gene
Oligo: S2 BIP (anti-sense)

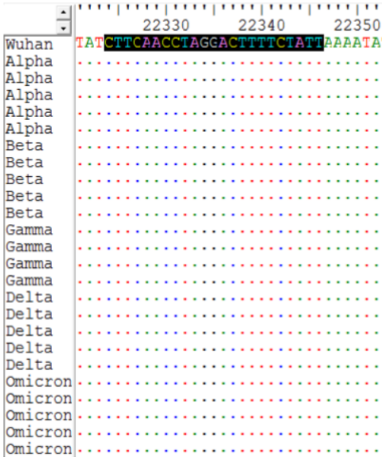

Target: S gene
Oligo: S2 LF (anti-sense)

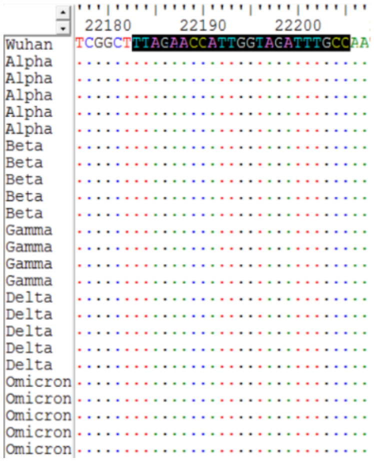

Target: S gene
Oligo: S2 LB

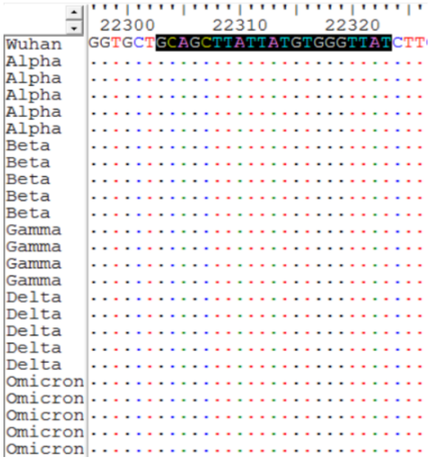

Target: S gene
Oligo: S2 Swarm F1c (anti-sense)

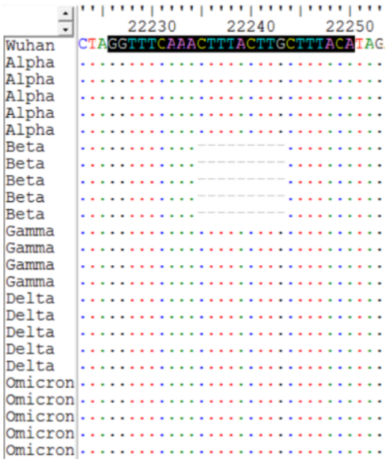

Target: S gene
Oligo: S2 Swarm B1c

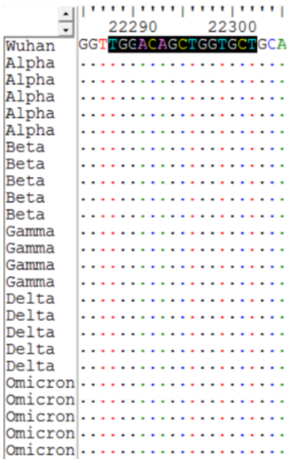

Target: S gene
Oligo: As-S2

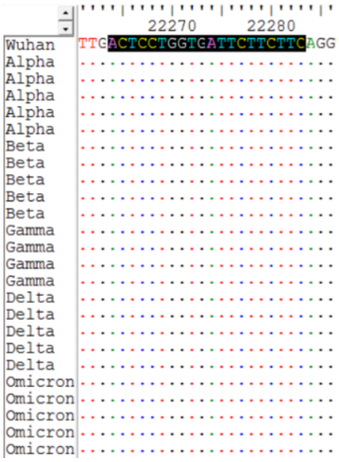

Target: S gene
Oligo: As-S6 (anti-sense)

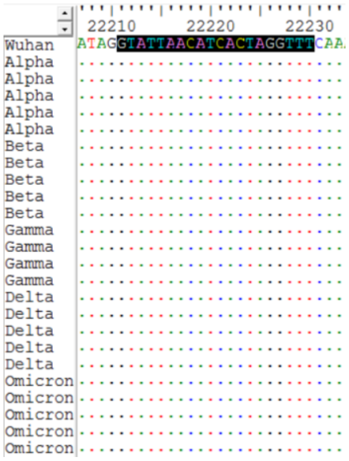

Title: Application of the amplification-free SERS-based CRISPR/Cas12a platform in the identification of SARS-CoV-2 from clinical samples

Target: N gene

Oligo: SARS-CoV-2 N gene crRNA

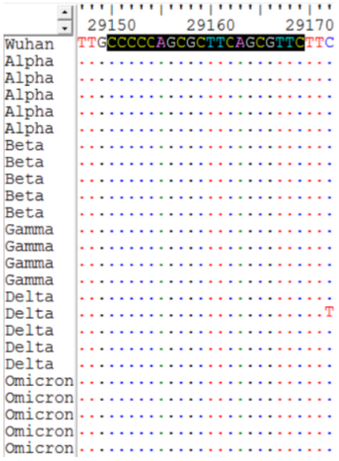

Title: Clinical validation of a Cas13-based assay for the detection of SARS-CoV-2 RNA

Target: S gene
Oligo: SL_S-RPA-Forward_v1

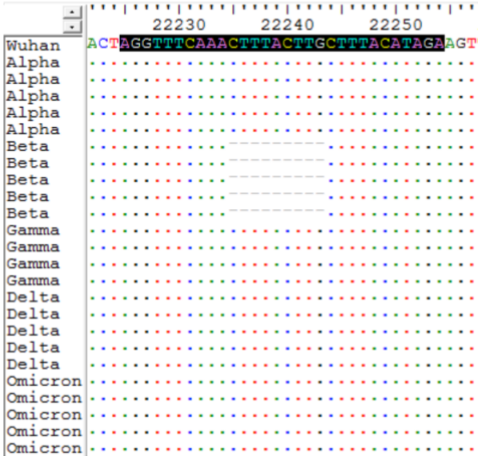

Target: S gene
Oligo: SL_S-RPA-Reverse_v1 (anti-sense)

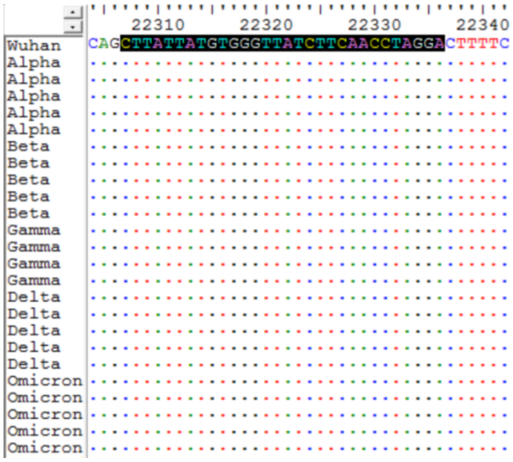

Target: Orf1ab
Oligo: SL_Orf1ab-RPAForward_v1

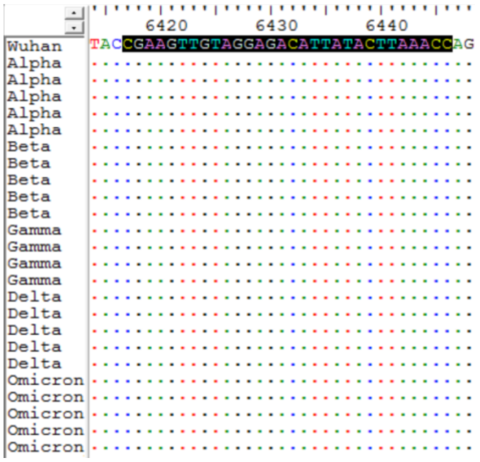

Target: Orf1ab
Oligo: SL_Orf1ab-RPAReverse_v1 (anti-sense)

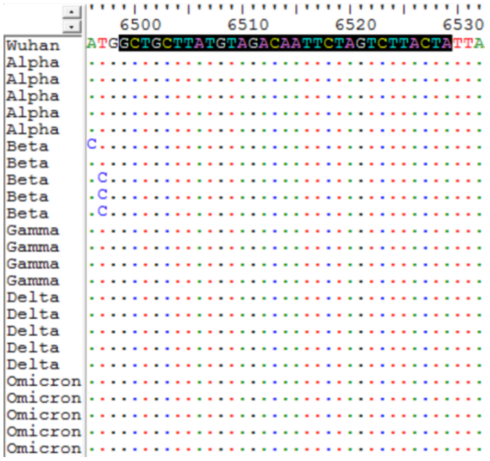

Target: Orf1b
Oligo: SI_Orf1b-RPAForward_v1

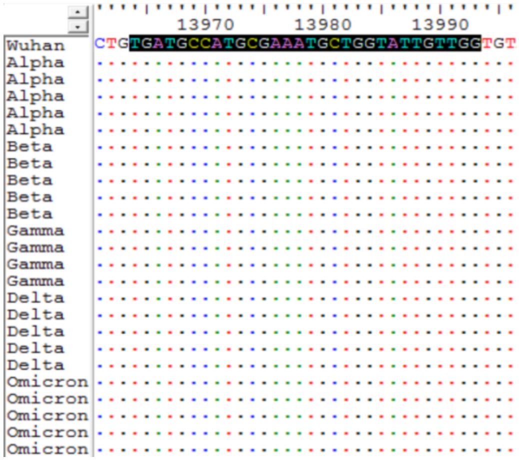

Target: Orf1b
Oligo: SI_Orf1b-RPAReverse_v1 (anti-sense)

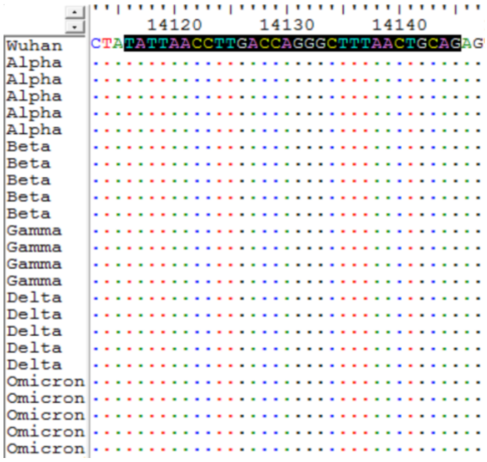

Target: N gene
Oligo: SI_N-RPA-Forward_v1

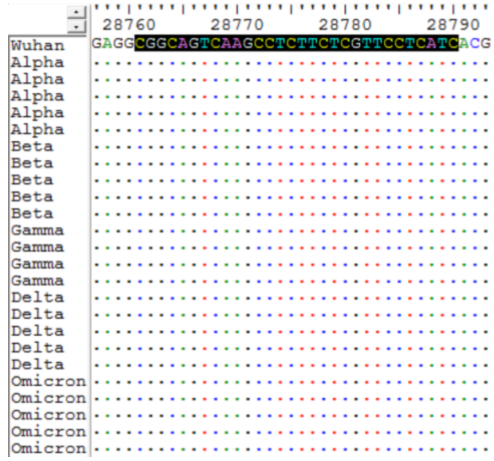

Target: N gene
Oligo: SI_N-RPA-Reverse_v1 (anti-sense)

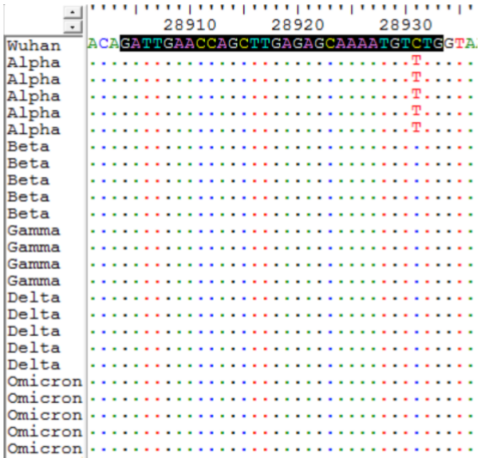

Target: S gene
Oligo: 13a_SL_S-crRNA_v1 (anti-sense)

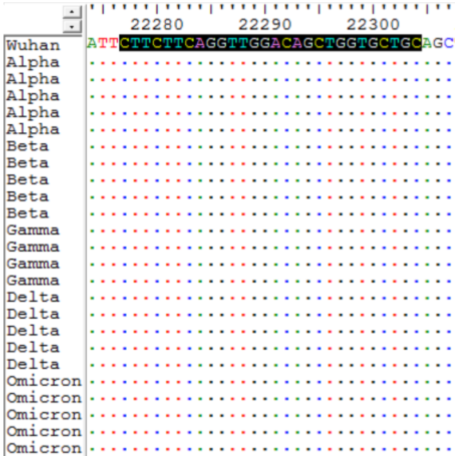

Target: Orf1ab
Oligo: 13a_SL_Orf1abcrRNA_v1 (anti-sense)

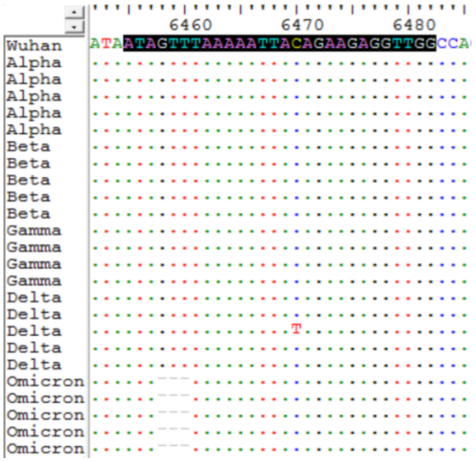

Target: Orf1b
Oligo: 13a_SI_Orf1b-crRNA_v1 (anti-sense)

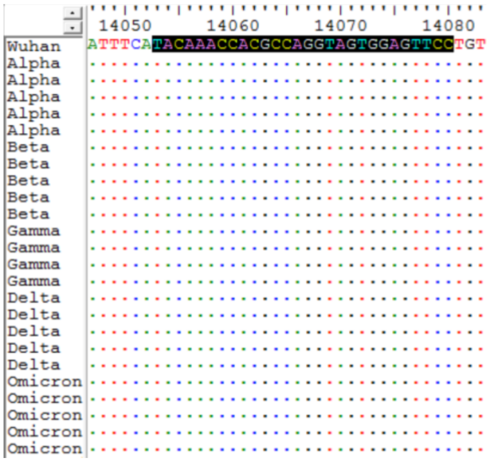

Target: N gene
Oligo: 13a_SI_N-crRNA_v1 (anti-sense)

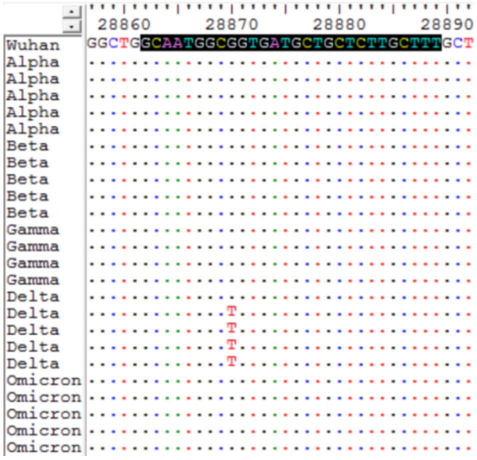

Title: Contamination-free visual detection of SARS-CoV-2 with CRISPR/Cas12a: A promising method in the point-of-care detection

Target: E gene
Oligo: RT-LAMP E-F3

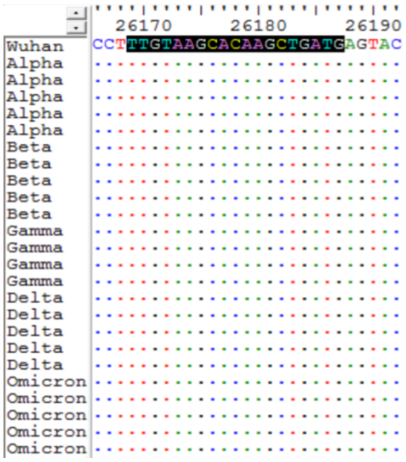

Target: E gene
Oligo: RT-LAMP E-B3 (anti-sense)

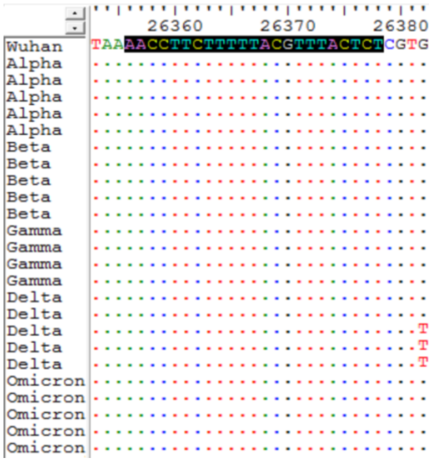

Target: E gene
Oligo: RT-LAMP E-FIP

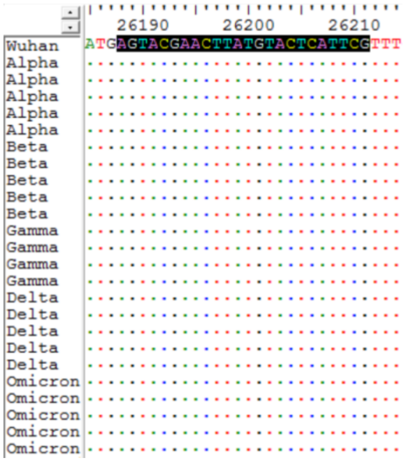

Target: E gene
Oligo: RT-LAMP E-BIP (anti-sense)

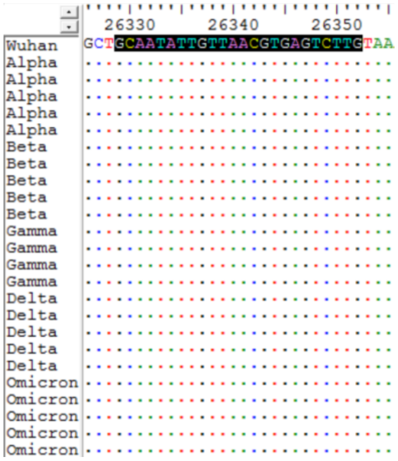

Target: E gene
Oligo: RT-LAMP E-LF (anti-sense)

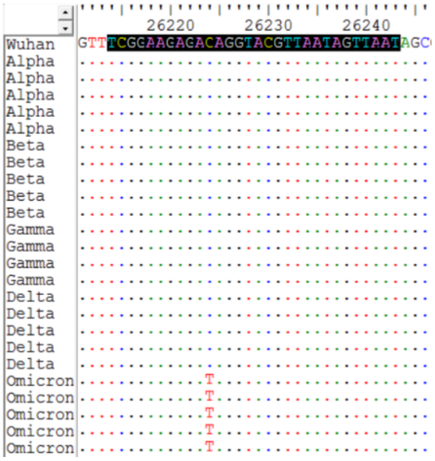

Target: E gene
Oligo: RT-LAMP E-LB

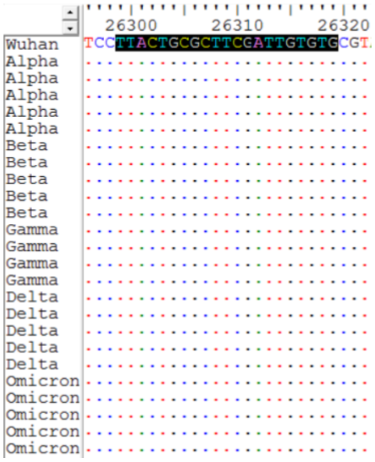

Target: Orf1ab
Oligo: RT-LAMP ORF-F3

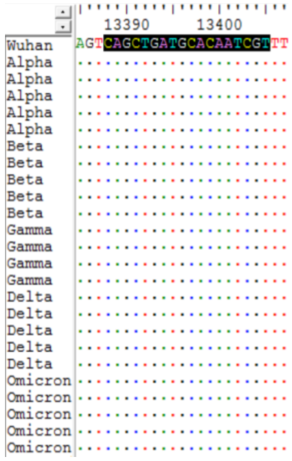

Target: Orf1ab
Oligo: RT-LAMP ORF-B3 (anti-sense)

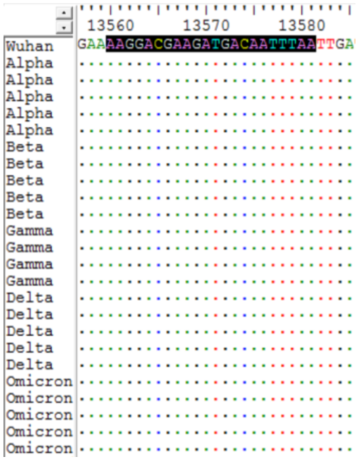

Target: Orf1ab
Oligo: RT-LAMP ORF-FIP

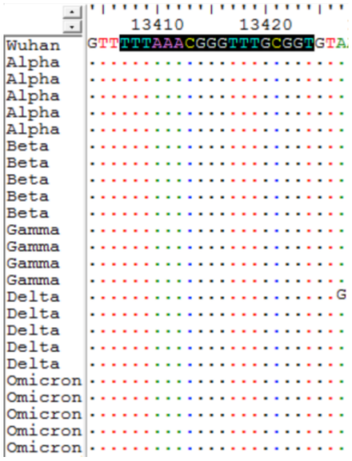

Target: Orf1ab
Oligo: RT-LAMP ORF-BIP (anti-sense)

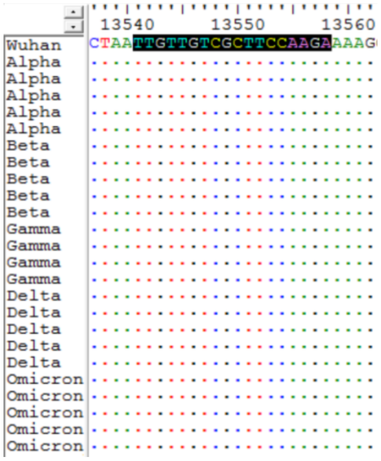

Target: Orf1ab
Oligo: RT-LAMP ORF-LF (anti-sense)

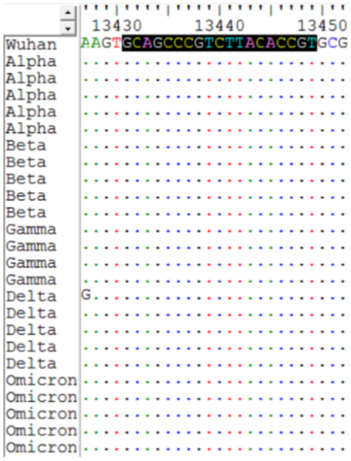

Target: Orf1ab
Oligo: RT-LAMP ORF-LB

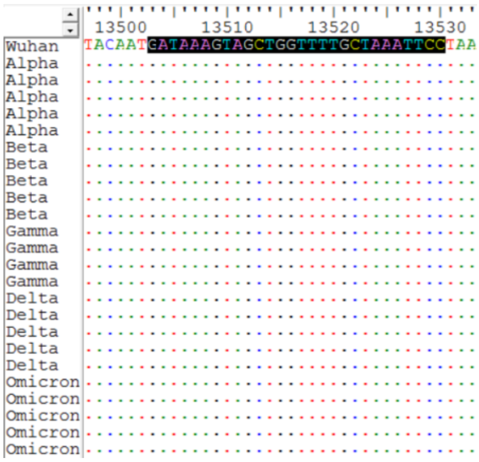

Target: N gene

Oligo: RT-LAMP N-F3

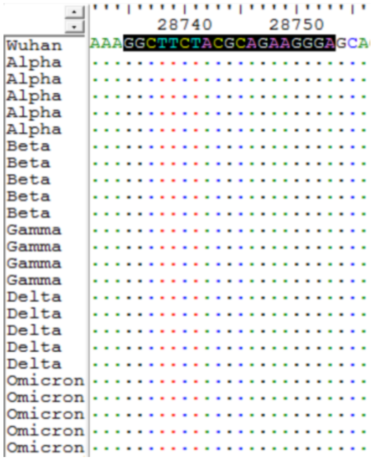

Target: N gene

Oligo: RT-LAMP N-B3 (anti-sense)

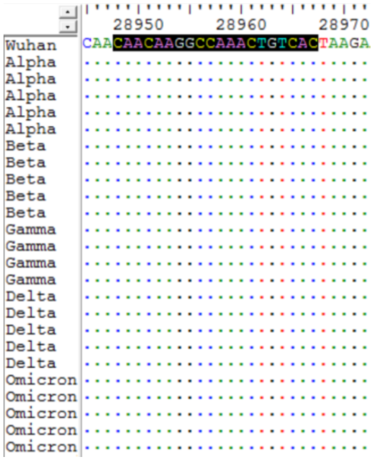

Target: N gene

Oligo: RT-LAMP N-FIP

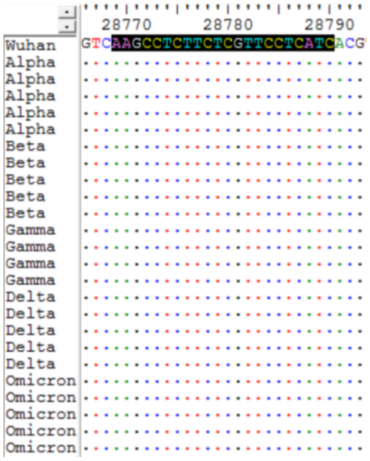

Target: N gene

Oligo: RT-LAMP N-BIP (anti-sense)

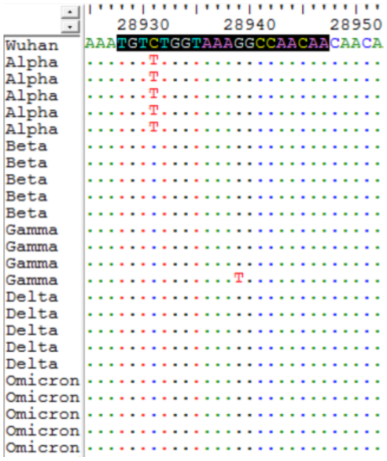

Target: N gene

Oligo: RT-LAMP N-LF (anti-sense)

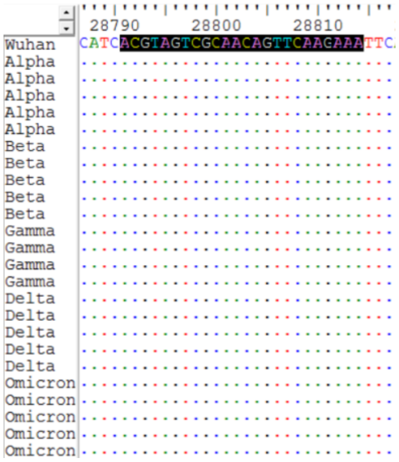

Target: N gene

Oligo: RT-LAMP N-LB

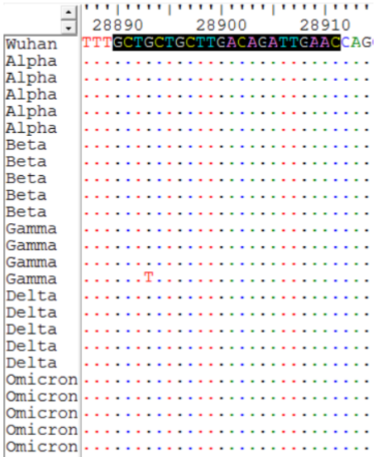

Target: E gene
Oligo: E-gRNA

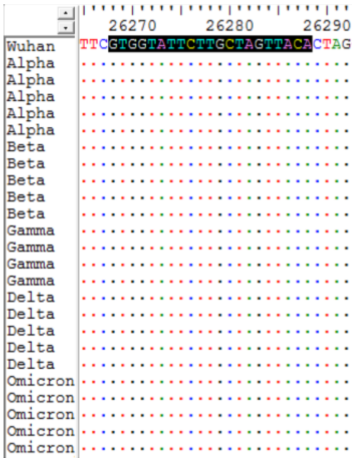

Target: Orf1ab
Oligo: ORF-gRNA

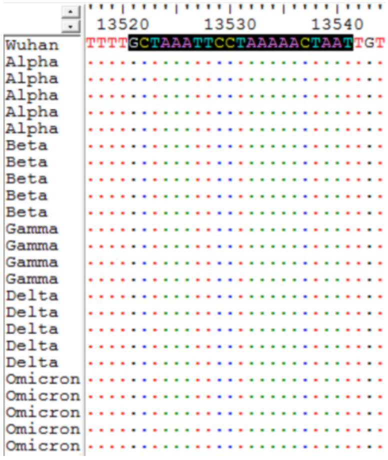

Target: N gene
Oligo: N-gRNA

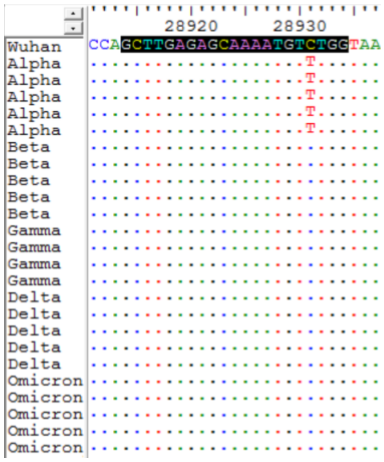

Title: CRISPR/Cas12a Technology Combined with RT-ERA for Rapid and Portable SARS-CoV-2 Detection

Target: N gene
Oligo: F

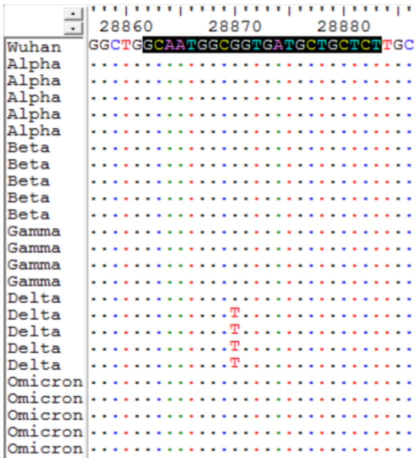

Target: N gene
Oligo: R (anti-sense)

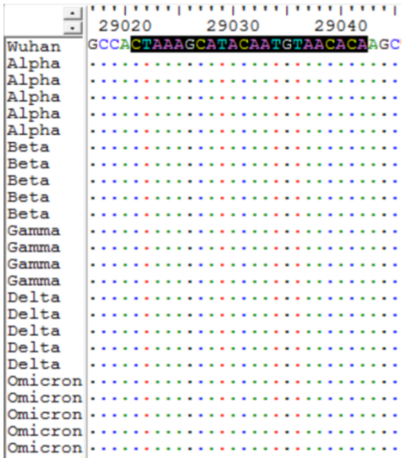

Target: N gene
Oligo: crRNA

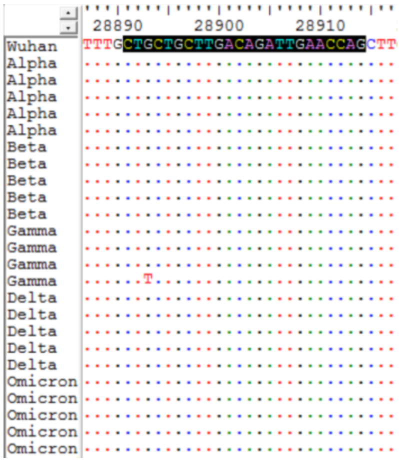

Target: Orf1ab
Oligo: crRNA (anti-sense)

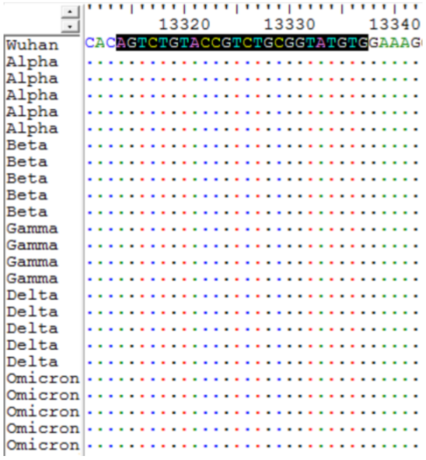

Target: Orf1ab
Oligo: F

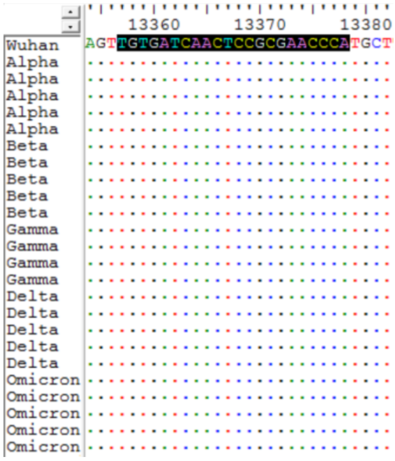

Target: Orf1ab
Oligo: R (anti-sense)

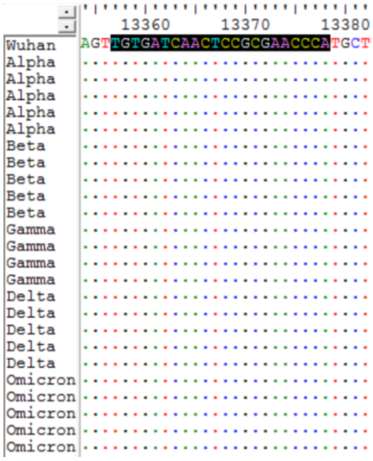

Title: CRISPR/Cas12a-mediated gold nanoparticle aggregation for colorimetric detection of SARS-CoV-2

Target: N gene
Oligo: N_F3

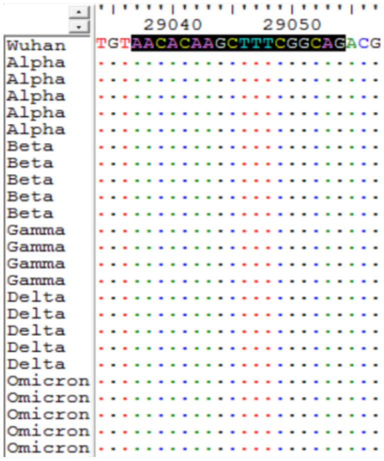

Target: N gene
Oligo: N_B3 (anti-sense)

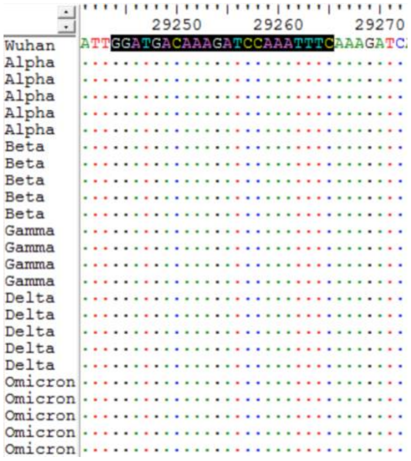

Target: N gene
Oligo: N_FIP

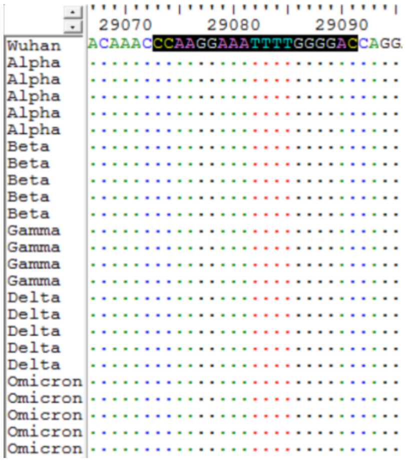

Target: N gene
Oligo: N_BIP (anti-sense)

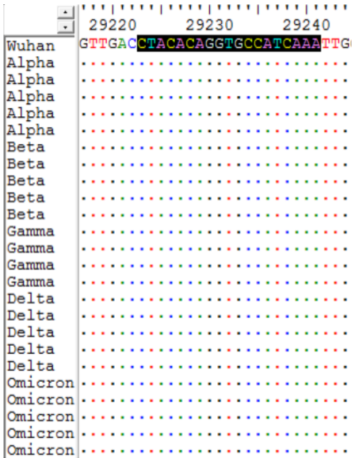

Target: N gene
Oligo: N-FL (anti-sense)

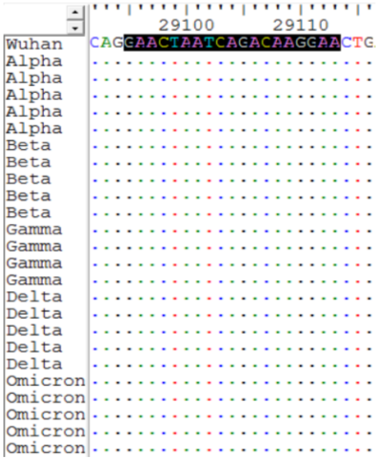

Target: N gene
Oligo: N-BL

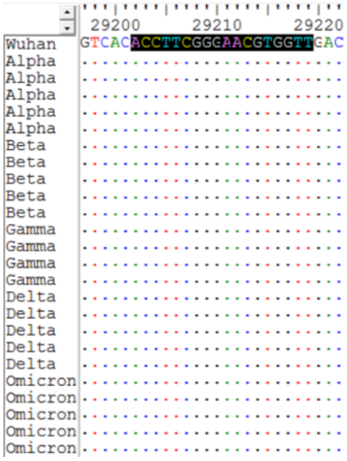

Target: E gene
Oligo: E_F3

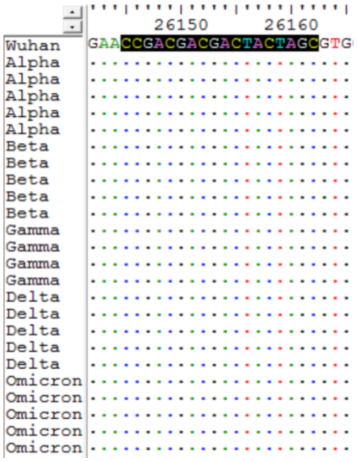

Target: E gene
Oligo: E_B3 (anti-sense)

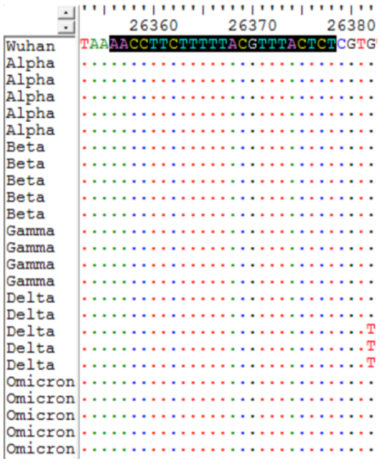

Target: E gene
Oligo: E_FIP

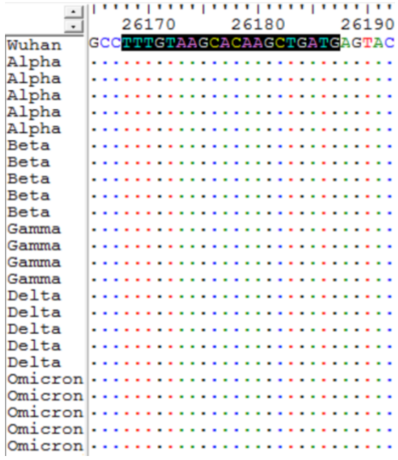

Target: E gene
Oligo: E_BIP (anti-sense)

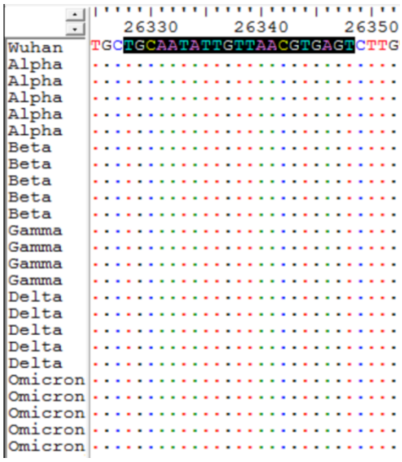

Target: E gene
Oligo: E_FL (anti-sense)

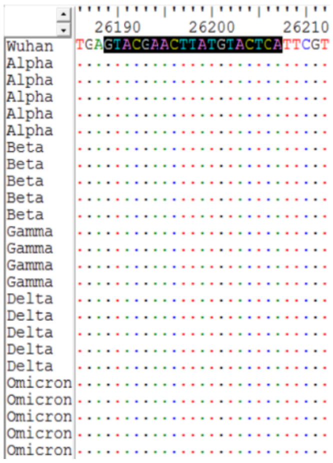

Target: E gene
Oligo: E_BL

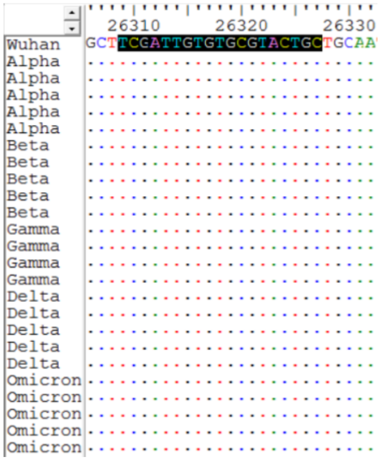

Target: N gene

Oligo: N-gRNA

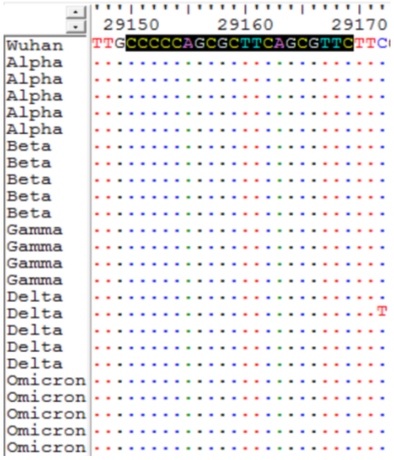

Target: E gene

Oligo: E-gRNA

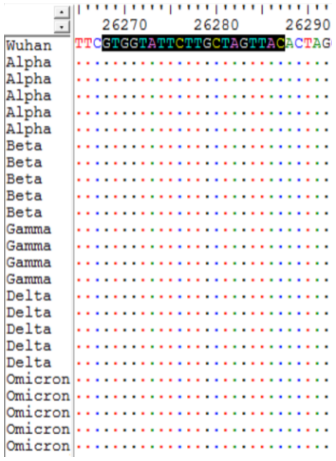

Title: CRISPR-Cas12-based detection of SARS-CoV-2

Target: N gene

Oligo: F3 2019-nCoV N-gene

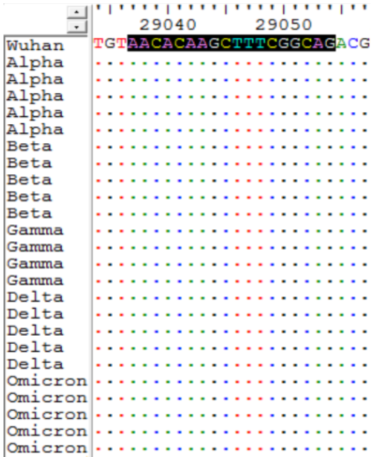

Target: N gene

Oligo: B3 2019-nCoV N-gene (anti-sense)

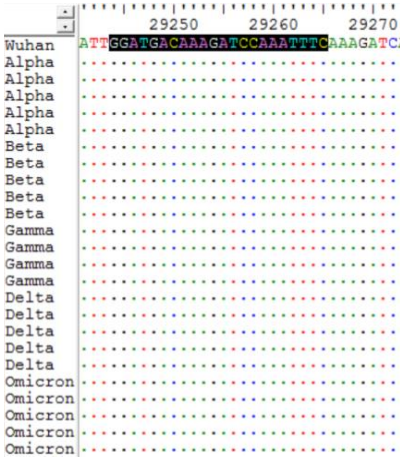

Target: N gene

Oligo: FIP 2019-nCoV N-gene

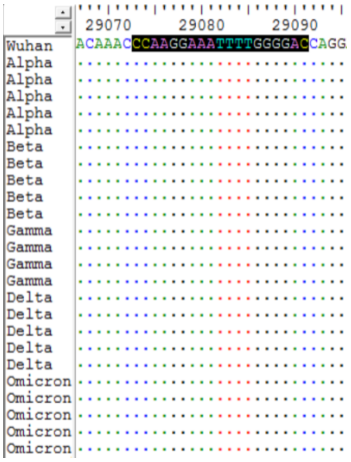

Target: N gene

Oligo: BIP 2019-nCoV N-gene (anti-sense)

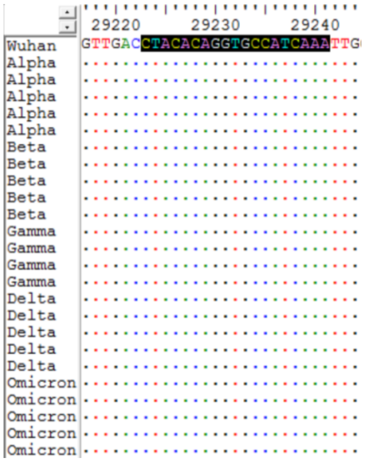

Target: N gene

Oligo: LF 2019-nCoV N-gene (anti-sense)

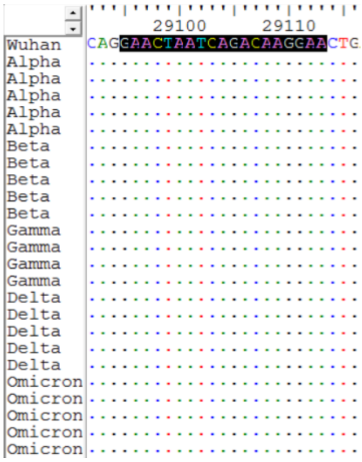

Target: N gene

Oligo: LB 2019-nCoV N-gene

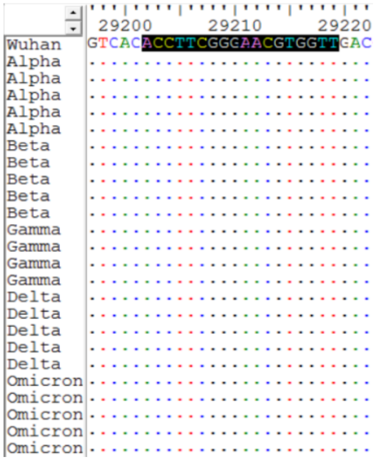

Target: E gene
Oligo: F3 2019-nCoV E-gene

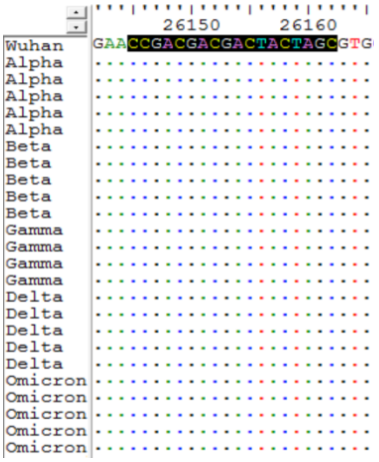

Target: E gene
Oligo: B3 2019-nCoV E-gene (anti-sense)

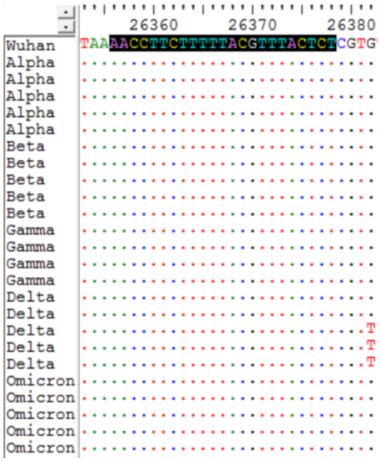

Target: E gene
Oligo: FIP 2019-nCoV E-gene

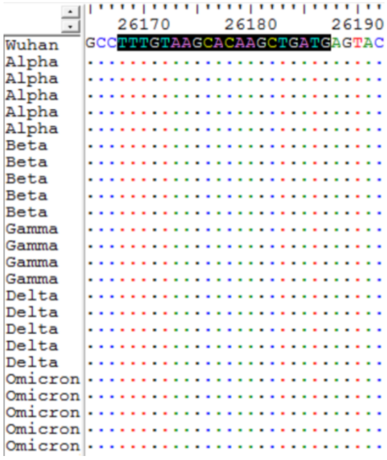

Target: E gene
Oligo: BIP 2019-nCoV E-gene (anti-sense)

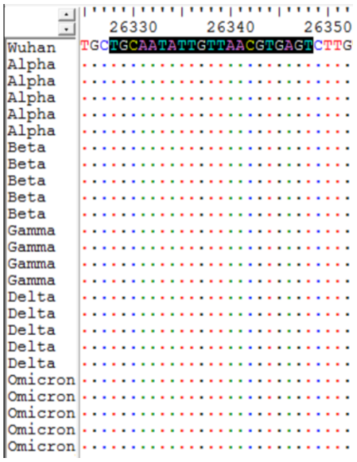

Target: E gene
Oligo: LF 2019-nCoV E-gene

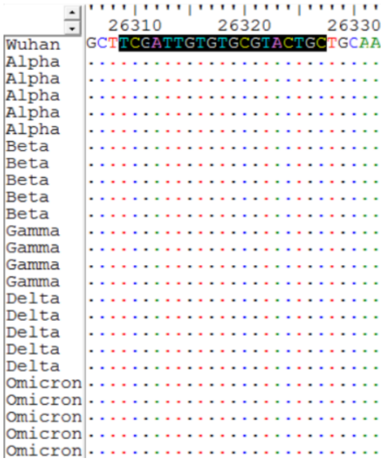

Target: E gene
Oligo: LB 2019-nCoV E-gene (anti-sense)

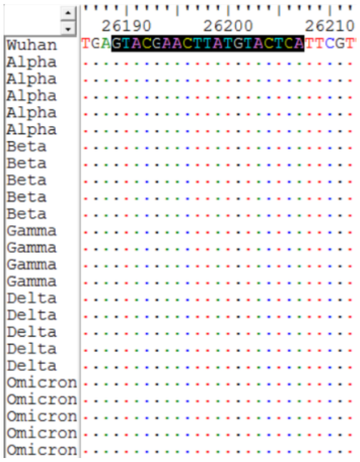

Target: N gene

Oligo: N-gene gRNA #1

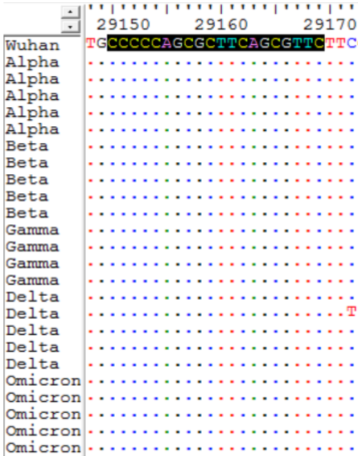

Target: N gene

Oligo: N-gene gRNA #2 (anti-sense)

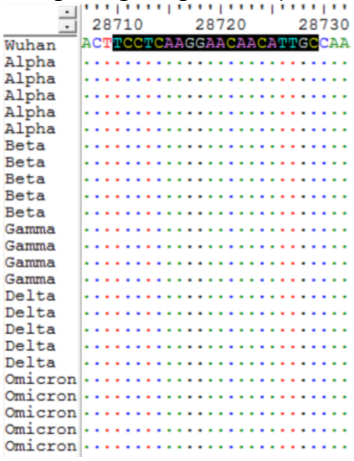

Target: E gene

Oligo: E-gene gRNA #1

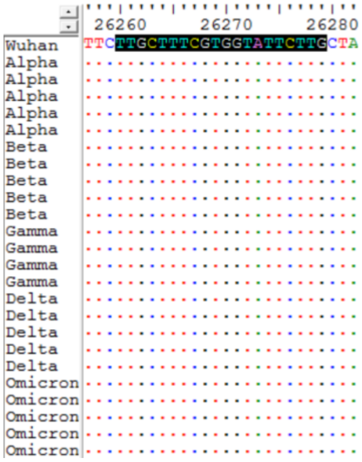

Target: E gene

Oligo: E-gene gRNA #2

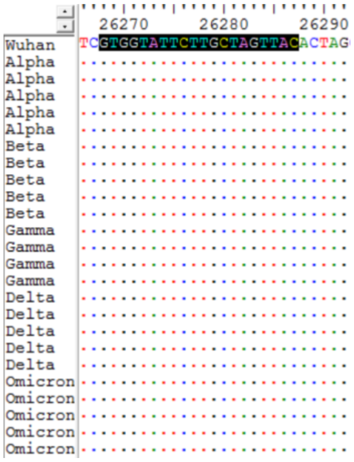

Title: Detection of Infectious Viruses Using CRISPR-Cas12-Based Assay

Target: S gene
Oligo: SARS-CoV-2 S FP

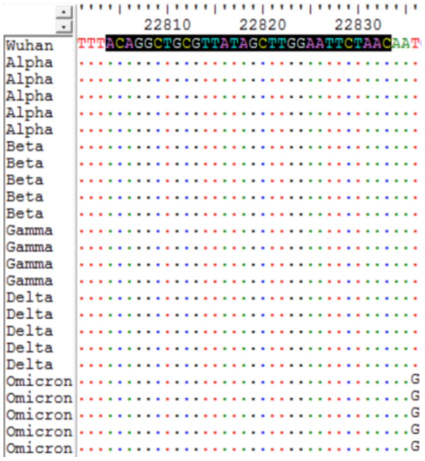

Target: S gene
Oligo: SARS-CoV-2 S RP

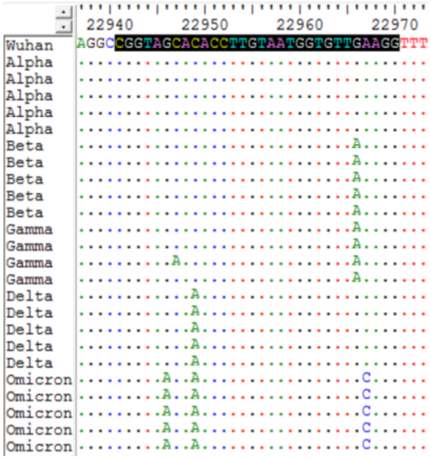

Target: S gene
Oligo: SARS-CoV-2 T1 gRNA

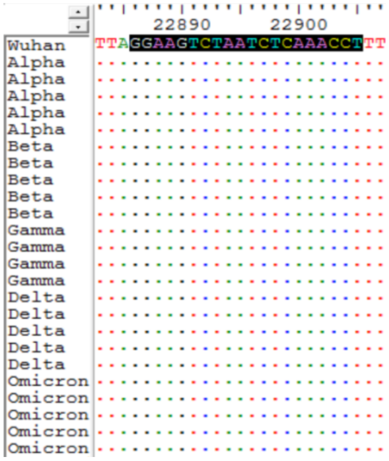

Target: S gene
Oligo: SARS-CoV-2 G2 gRNA

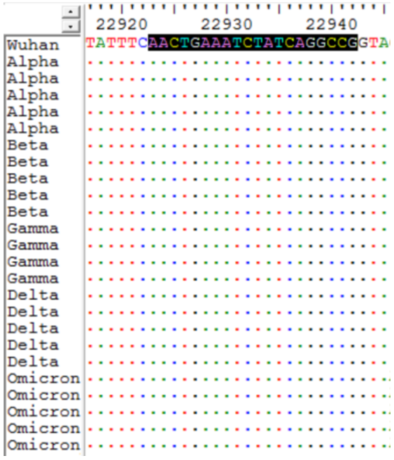

Title: Detection of SARS-CoV-2 by CRISPR/Cas12a-Enhanced Colorimetry

Target: E gene

Oligo: E-gene F

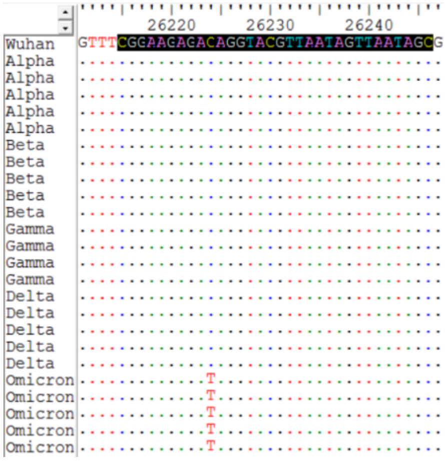

Target: E gene

Oligo: E-gene R (anti-sense)

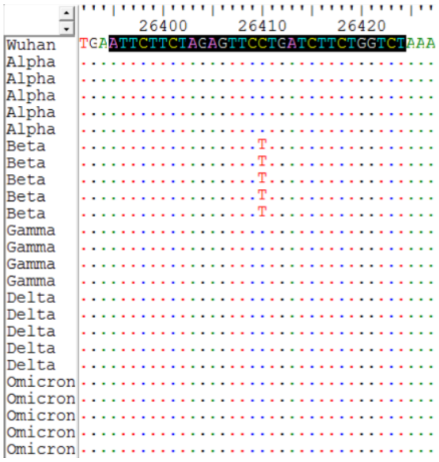

Target: Orf1ab

Oligo: O-gene F

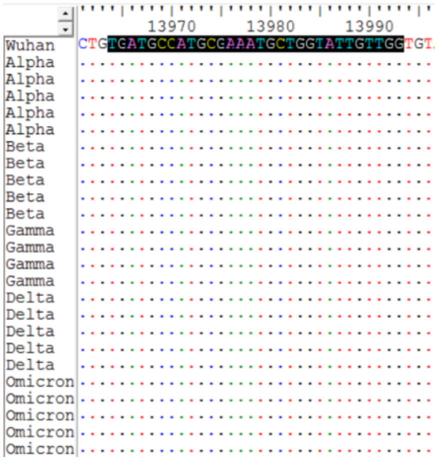

Target: Orf1ab

Oligo: O-gene R (anti-sense)

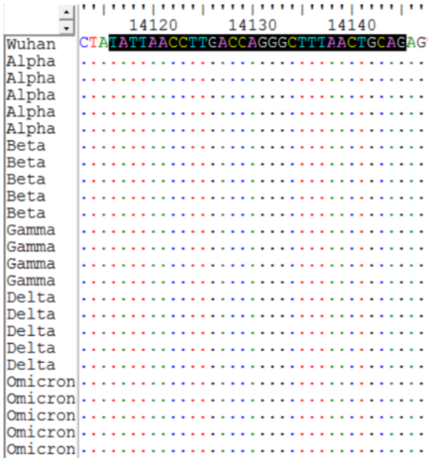

Target: N gene

Oligo: N-gene region1 F

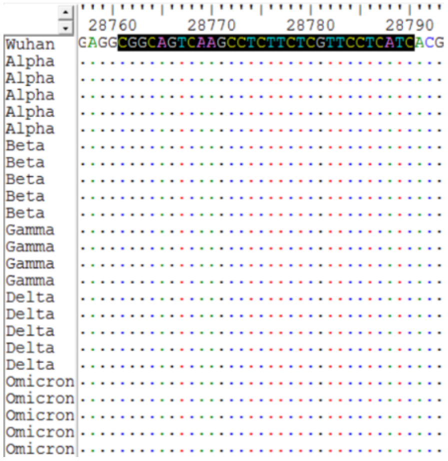

Target: N gene

Oligo: N-gene region1 R (anti-sense)

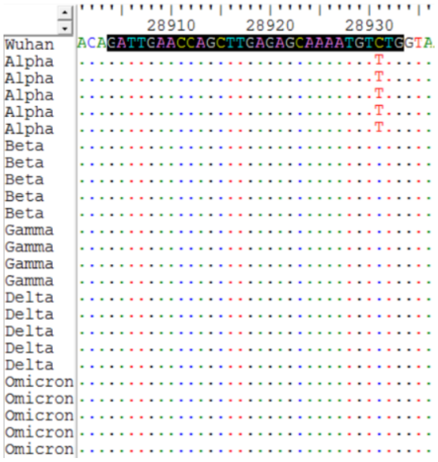

Target: N gene

Oligo: N-gene region2 F

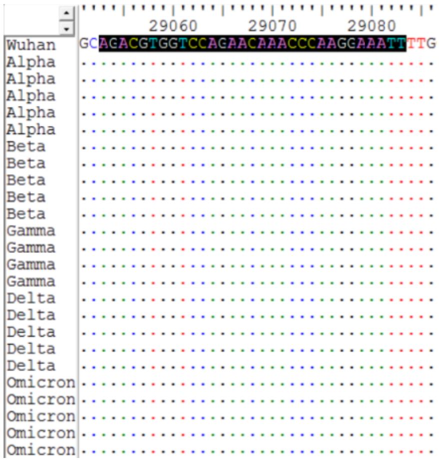

Target: N gene

Oligo: N-gene region2 R (anti-sense)

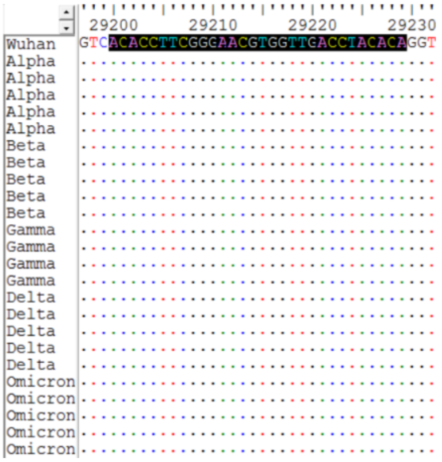

Target: E gene

Oligo: E-gene crRNA

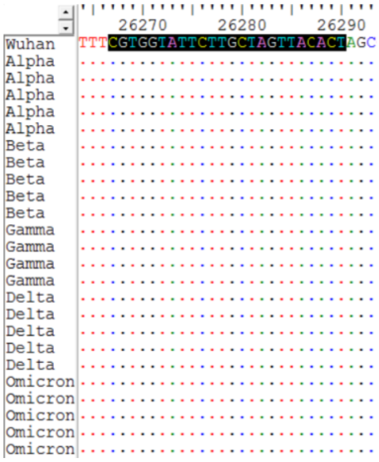

Target: Orf1ab

Oligo: O-gene crRNA

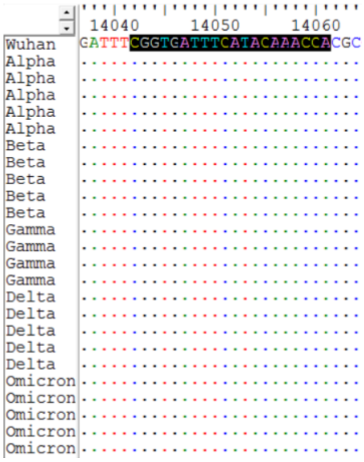

Target: N gene

Oligo: N-gene crRNA region 1 (anti-sense)

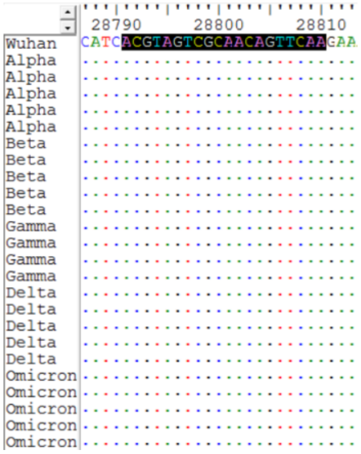

Target: N gene

Oligo: N-gene crRNA region 2

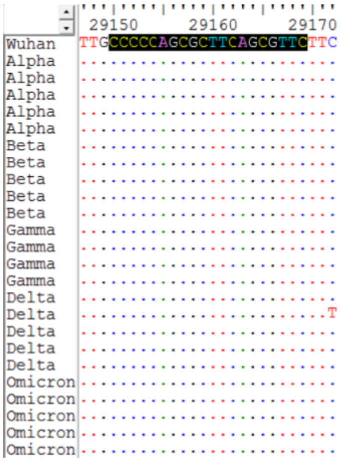

Title: Detection of severe acute respiratory syndrome coronavirus 2 and influenza viruses based on CRISPR-Cas12a

Target: S gene
Oligo: SARS2_spike1-F

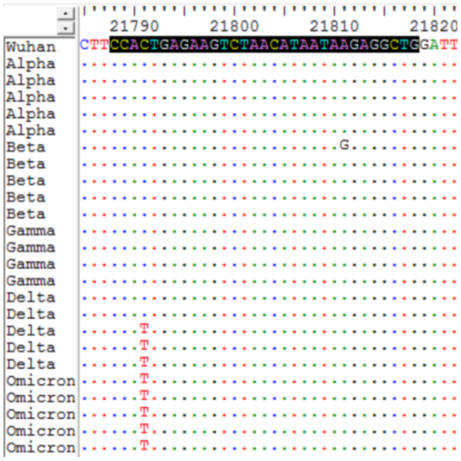

Target: S gene
Oligo: SARS2_spike1-R (anti-sense)

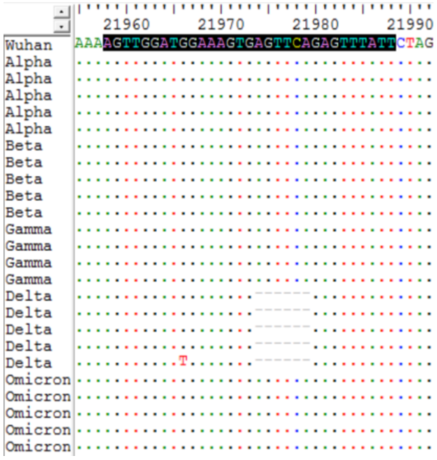

Target: S gene
Oligo: SARS2_spike2-F

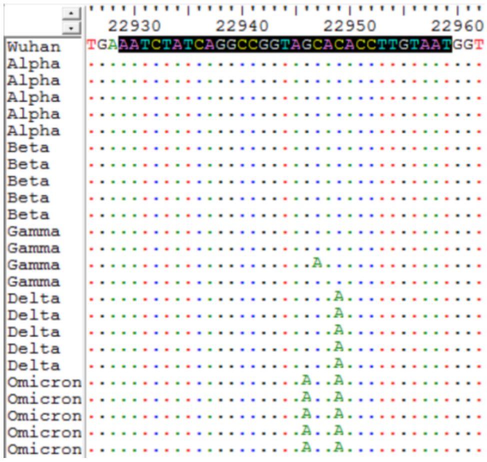

Target: S gene
Oligo: SARS2_spike2-R (anti-sense)

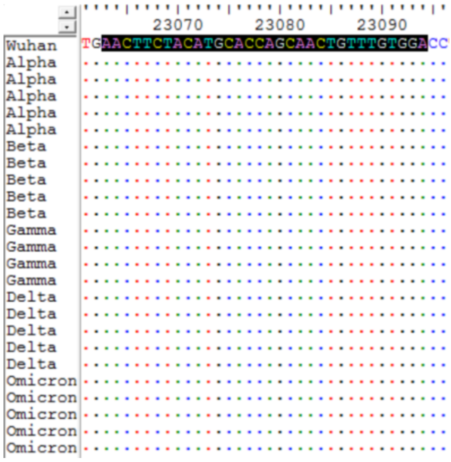

Target: S gene
Oligo: SARS2-S1

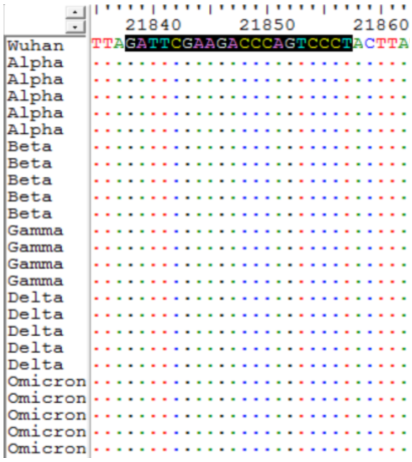

Target: S gene
Oligo: SARS2-S2

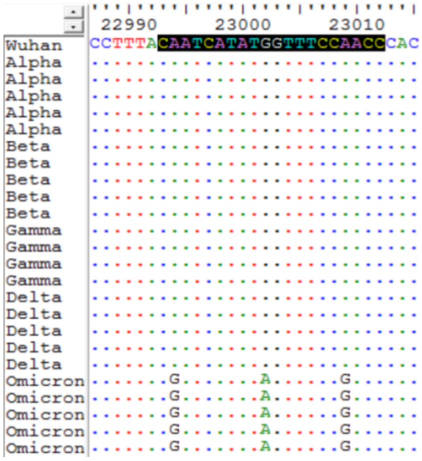

Title: Detection of the SARS-CoV-2 D614G mutation using engineered Cas12a guide RNA

Target: E gene
Oligo: E-RPA-F

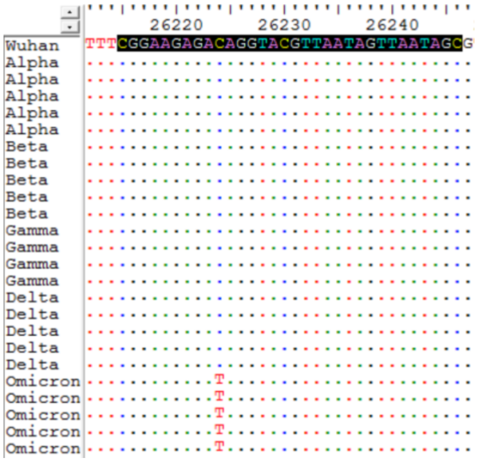

Target: E gene
Oligo: E-RPA-R

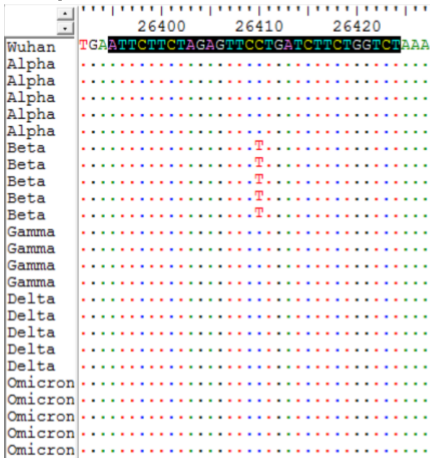

Target: E gene
Oligo: core E gene

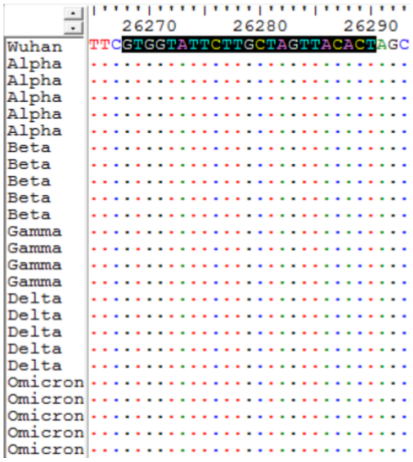

Title: Development and evaluation of a rapid CRISPR-based diagnostic for COVID-19

Target: Orf1ab
Oligo: forward primer

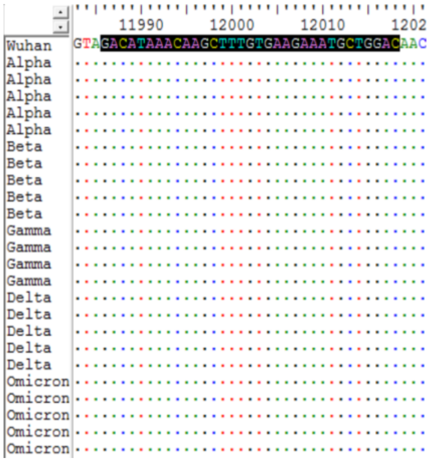

Target: Orf1ab
Oligo: reverse primer

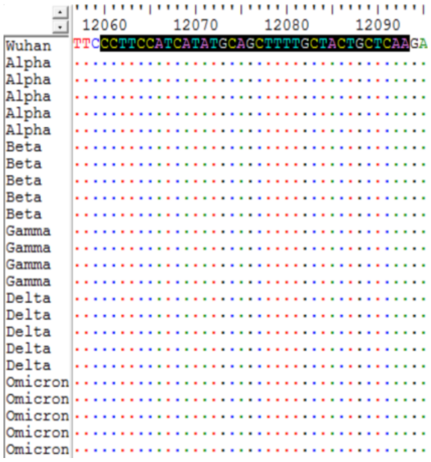

Target: Orf1ab
Oligo: gRNA for Orf1ab (anti-sense)

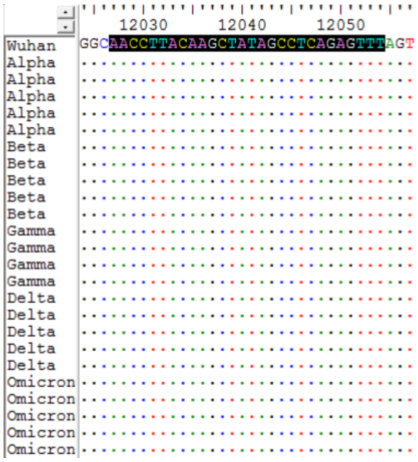

Title: Development of a Broadly Applicable Cas12a-Linked Beam Unlocking Reaction for Sensitive and Specific Detection of Respiratory Pathogens Including SARS-CoV-2

Target: S gene
Oligo: SARSCoV2-S-FWD-1

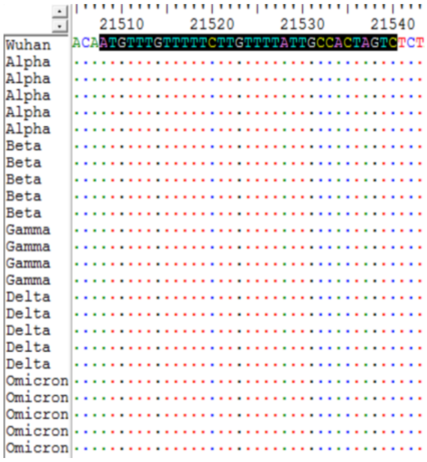

Target: S gene
Oligo: SARSCoV2-S-REV-1 (anti-sense)

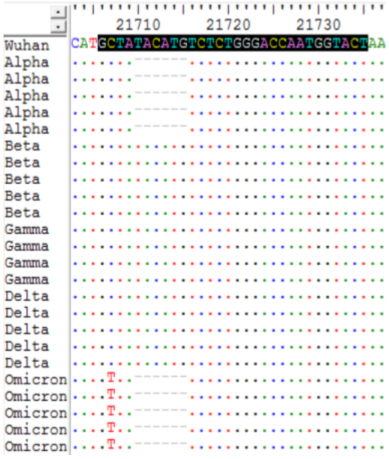

Target: S gene
Oligo: SARSCoV2-S-crRNA2

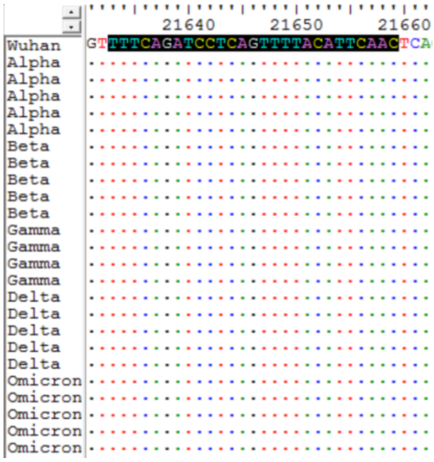

Title: Development of a Rapid and Sensitive CasRx-Based Diagnostic Assay for SARS-CoV-2

Target: S gene
Oligo: RPA-S3-F

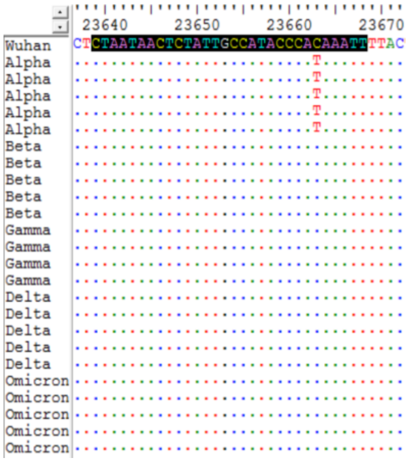

Target: S gene
Oligo: RPA-S3-R (anti-sense)

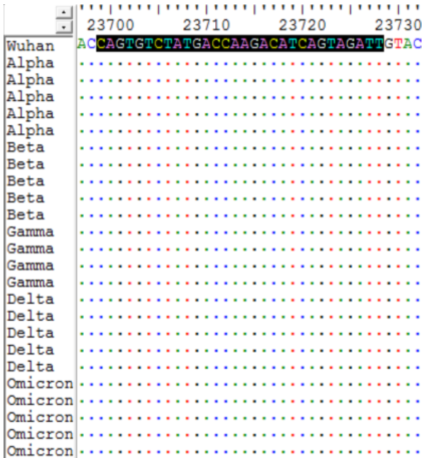

Target: N gene
Oligo: RPA-N1-F

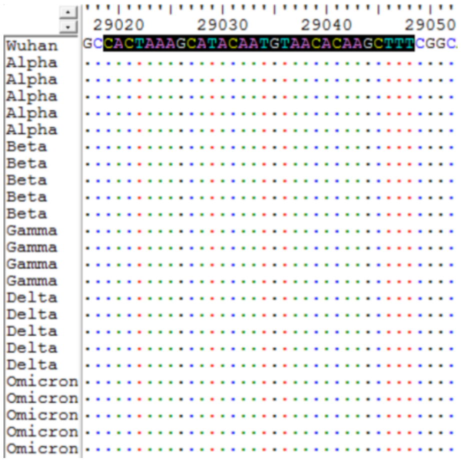

Target: N gene
Oligo: RPA-N1-R (anti-sense)

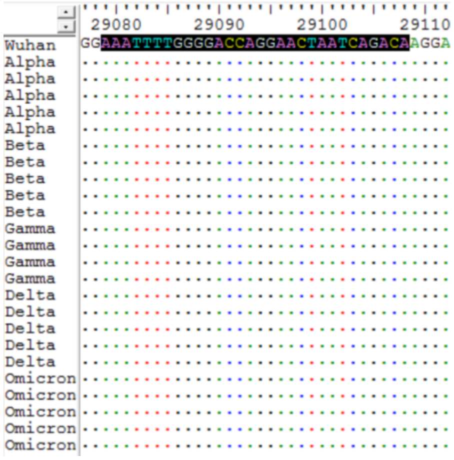

Target: S gene
Oligo: gRNA S3 (anti-sense)

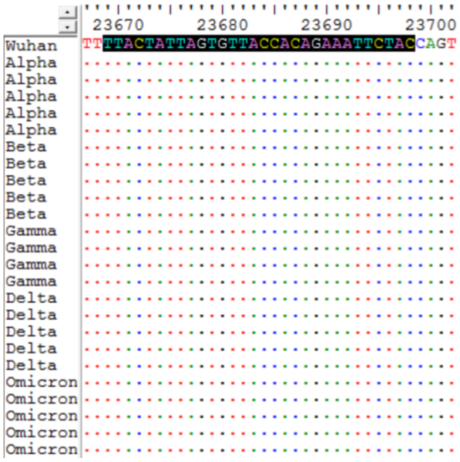

Target: N gene
Oligo: gRNA N1 (anti-sense)

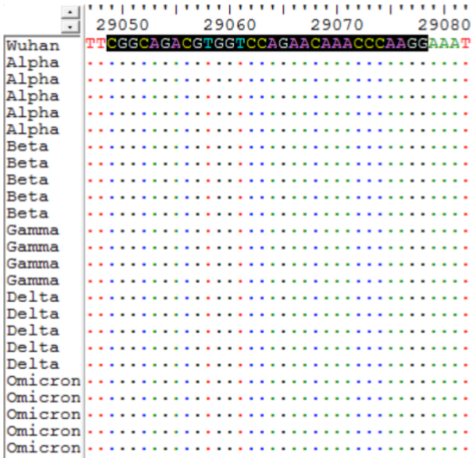

Title: Digital CRISPR/Cas-Assisted Assay for Rapid and Sensitive Detection of SARS-CoV-2

Target: N gene
Oligo: RPA Forward primer

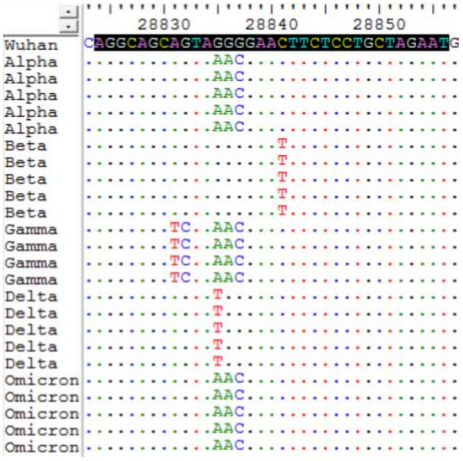

Target: N gene
Oligo: RPA Reverse primer (anti-sense)

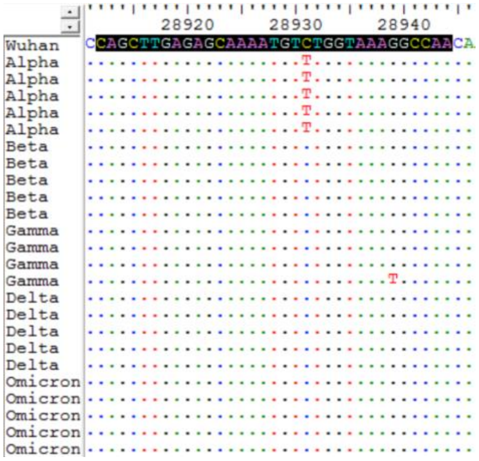

Target: N gene
Oligo: Lba Cas12a-guide RNA1 (anti-sense)

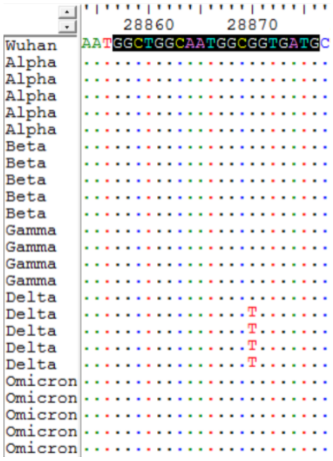

Target: N gene
Oligo: Lba Cas12a-guide RNA2

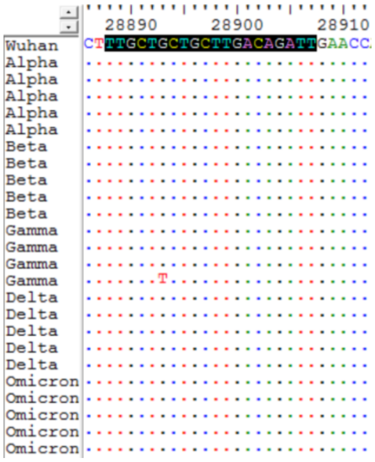

Title: Electric field-driven microfluidics for rapid CRISPR-based diagnostics and its application to detection of SARS-CoV-2

Target: N gene
Oligo: N-gene F3

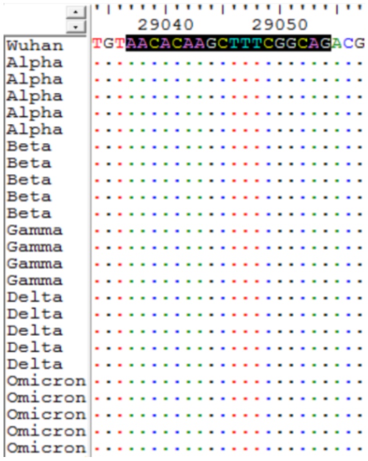

Target: N gene
Oligo: N-gene B3

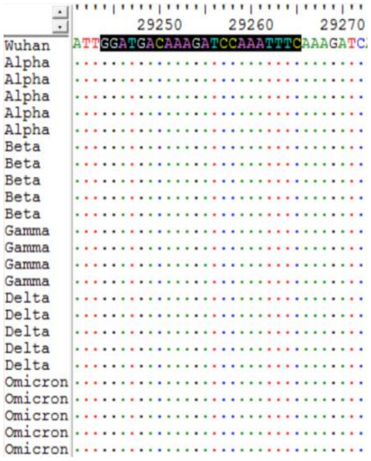

Target: N gene
Oligo: N-gene FIP

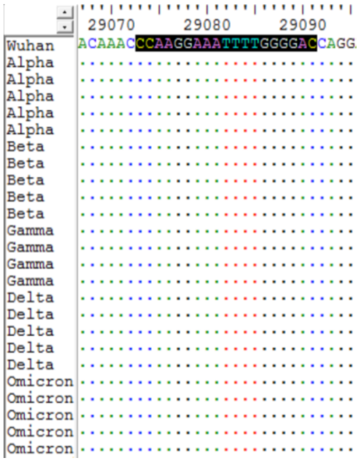

Target: N gene
Oligo: N-gene BIP

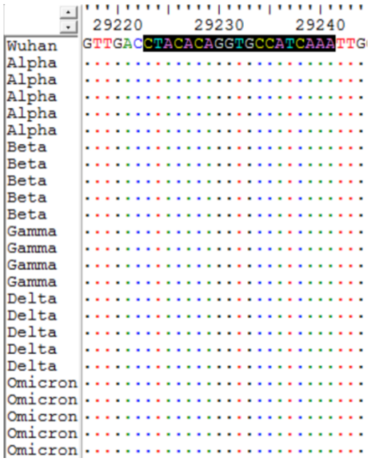

Target: N gene
Oligo: N gene-FL (anti-sense)

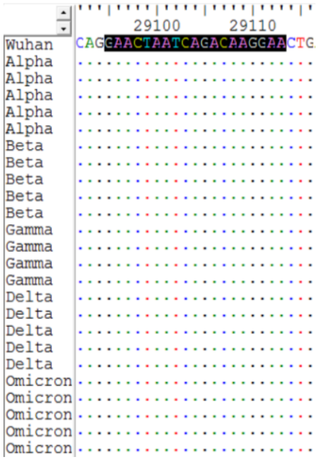

Target: N gene
Oligo: N gene-BL

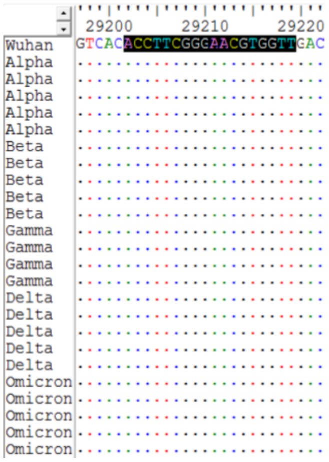

Target: E gene

Oligo: E gene_F3

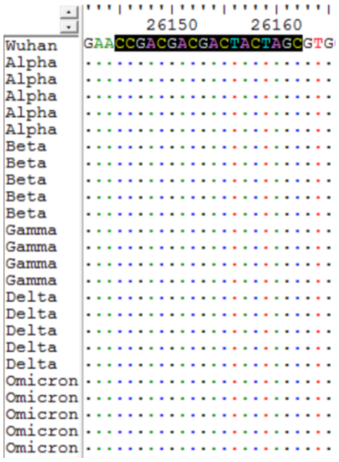

Target: E gene

Oligo: E gene_B3 (anti-sense)

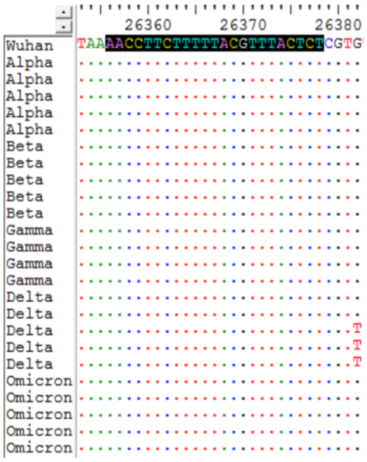

Target: E gene

Oligo: E gene_FIP

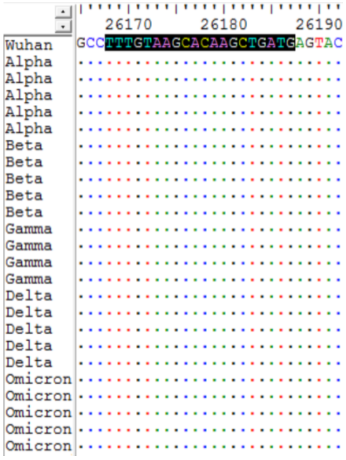

Target: E gene

Oligo: E gene_BIP (anti-sense)

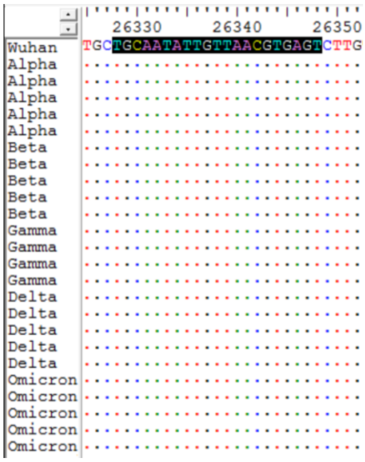

Target: E gene

Oligo: E gene_FL (anti-sense)

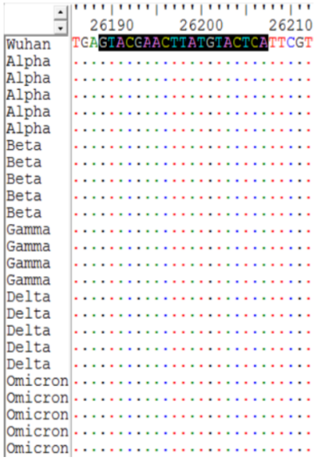

Target: E gene

Oligo: E gene_BL

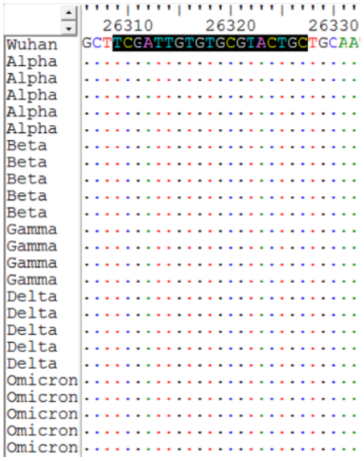

Target: N gene

Oligo: N gene-gRNA

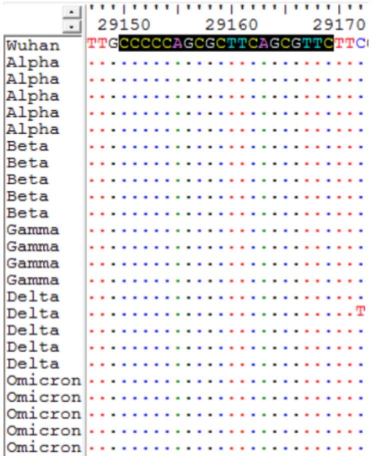

Target: E gene

Oligo: E gene-gRNA

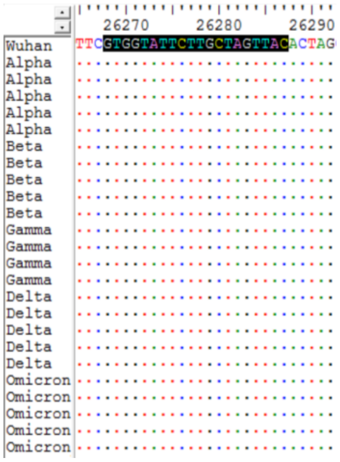

Title: Enhancement of trans-cleavage activity of Cas12a with engineered crRNA enables amplified nucleic acid detection

Target: N gene

Oligo: F3_LAMP_N1

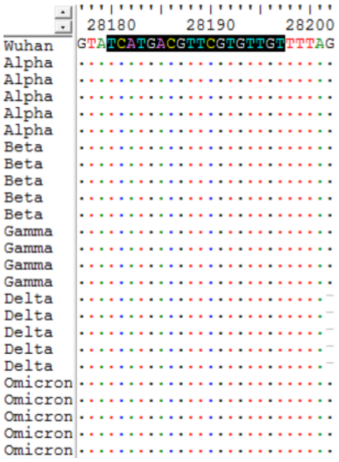

Target: N gene

Oligo: B3_LAMP_N1 (anti-sense)

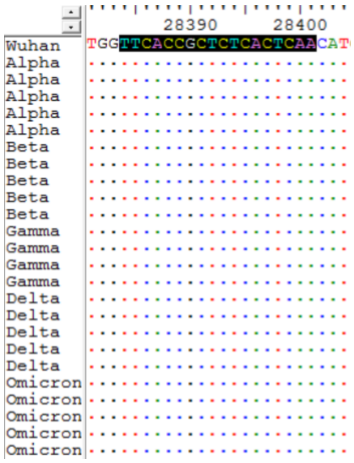

Target: N gene

Oligo: FIP_LAMP_N1

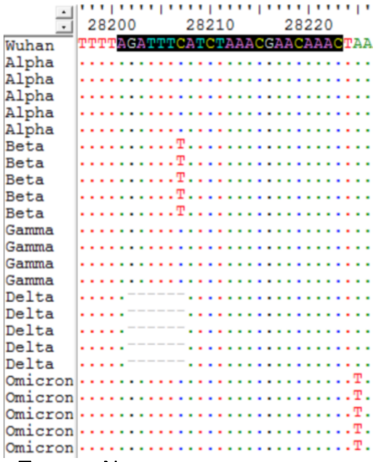

Target: N gene

Oligo: BIP_LAMP_N1 (anti-sense)

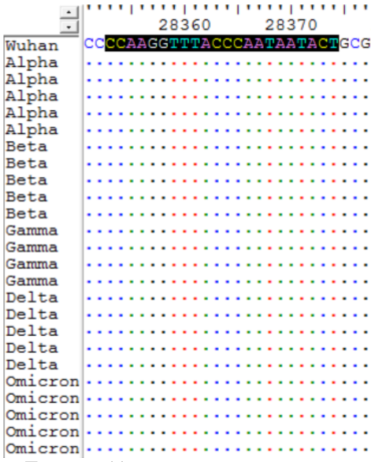

Target: N gene

Oligo: LF_LAMP_N1 (anti-sense)

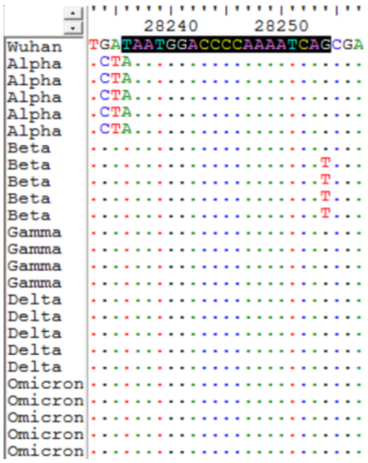

Target: N gene

Oligo: LB_LAMP_N1

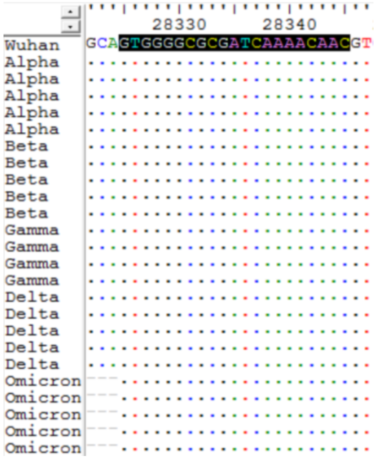

Target: N gene

Oligo: F3_LAMP_N2

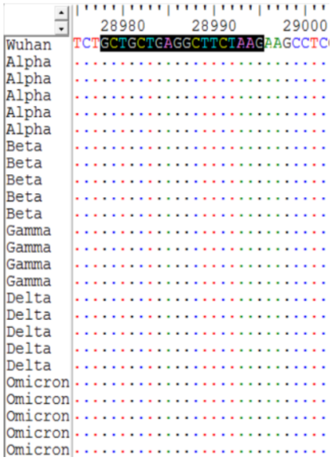

Target: N gene

Oligo: B3_LAMP_N2 (anti-sense)

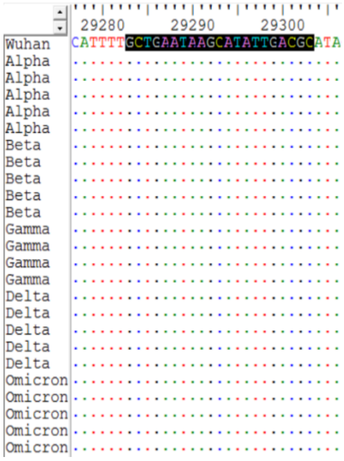

Target: N gene

Oligo: FIP_LAMP_N2

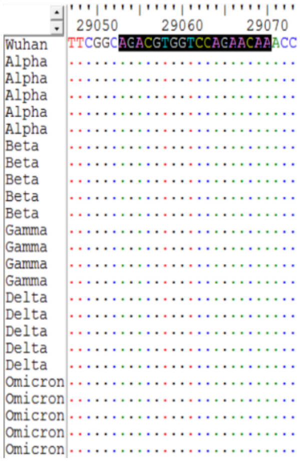

Target: N gene

Oligo: BIP_LAMP_N2 (anti-sense)

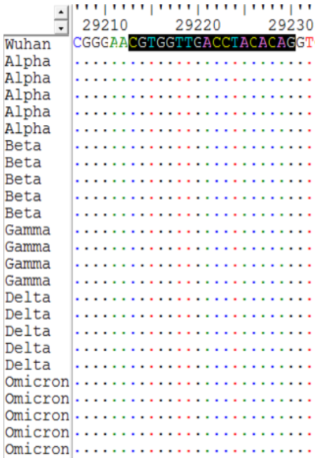

Target: N gene

Oligo: LF_LAMP_N2 (anti-sense)

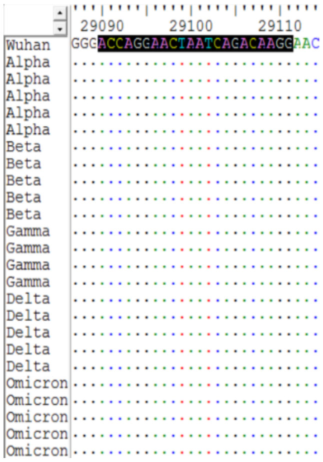

Target: N gene

Oligo: LB_LAMP_N2

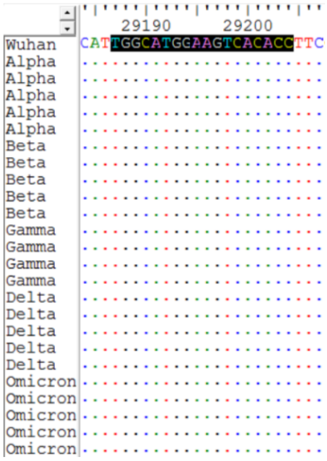

Target: N gene

Oligo: N1 crRNA

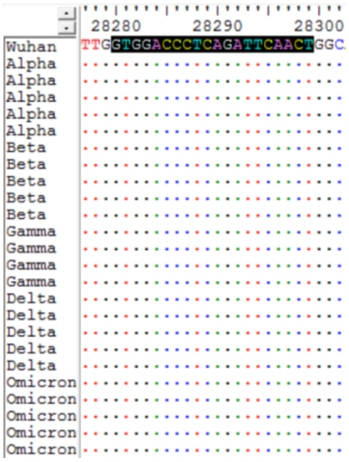

Target: N gene

Oligo: N2 crRNA (anti-sense)

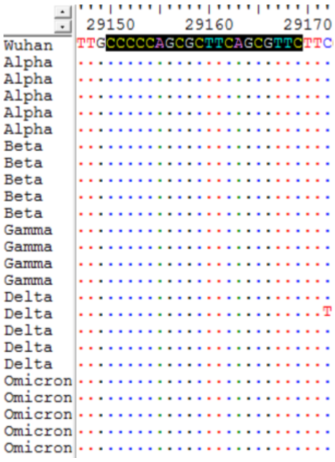

Title: Fluorescence polarization system for rapid COVID-19 diagnosis

Target: N gene
Oligo: N1-Forward RPA primer

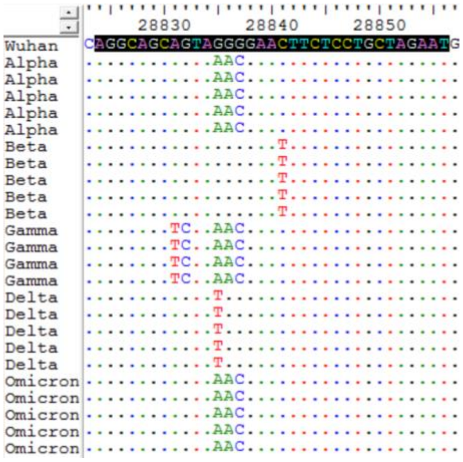

Target: N gene
Oligo: N1-Reverse RPA primer (anti-sense)

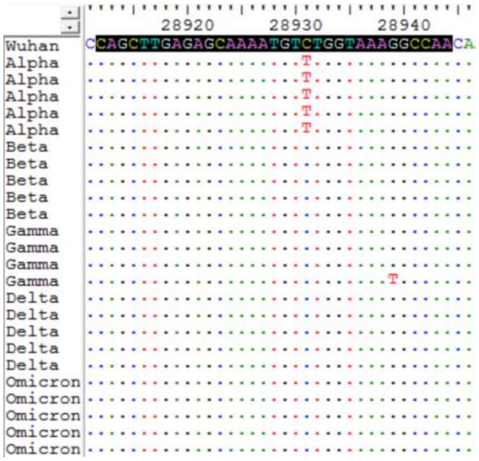

Target: N gene
Oligo: N2-Forward RPA primer

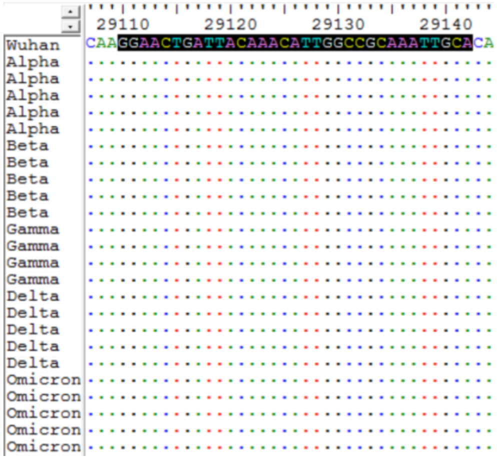

Target: N gene
Oligo: N2-Reverse RPA primer (anti-sense)

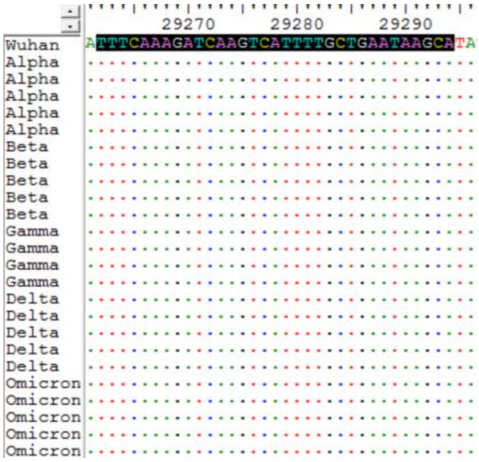

Target: N gene
Oligo: N1-Forward Cas12a gRNA (anti-sense)

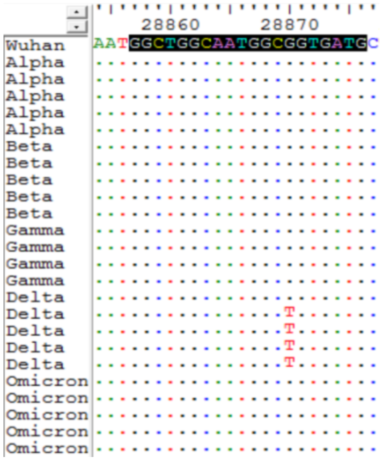

Target: N gene
Oligo: N1-Reverse Cas12a gRNA

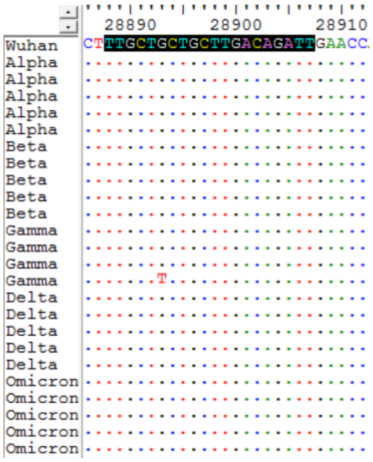

Target: N gene

Oligo: N2-Forward Cas12a gRNA (anti-sense)

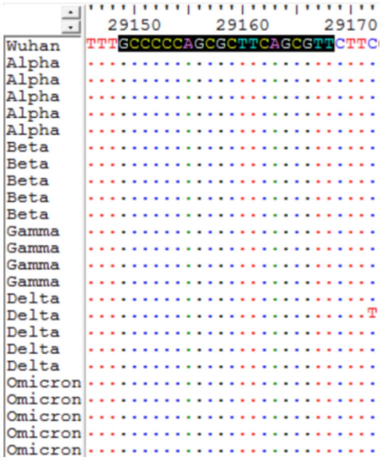

Target: N gene

Oligo: N2-Reverse Cas12a gRNA

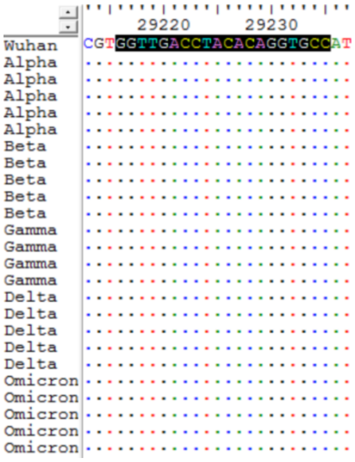

Title: Instrument-free, CRISPR-based diagnostics of SARS-CoV-2 using self-contained microfluidic system

Target: N gene
Oligo: FP_SARS-CoV-2

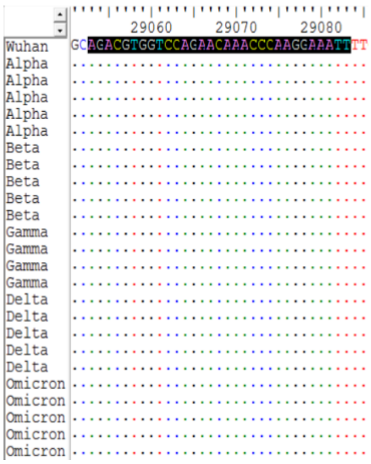

Target: N gene
Oligo: RP_SARS-CoV-2 (anti-sense)

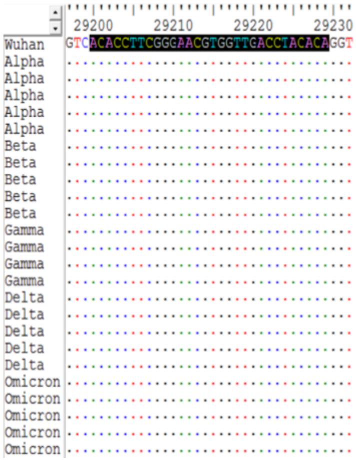

Target: N gene
Oligo: LbCas12a-crRNA-SARS-CoV-2

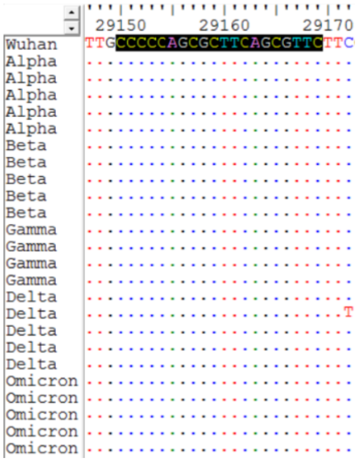

Title: iSCAN: An RT-LAMP-coupled CRISPR-Cas12 module for rapid, sensitive detection of SARS-CoV-2

Target: N gene

Oligo: F3-E-2

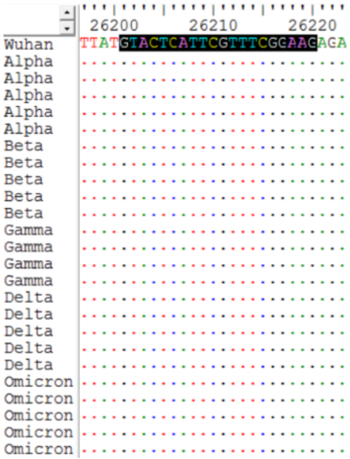

Target: N gene

Oligo: B3-E-1 (anti-sense)

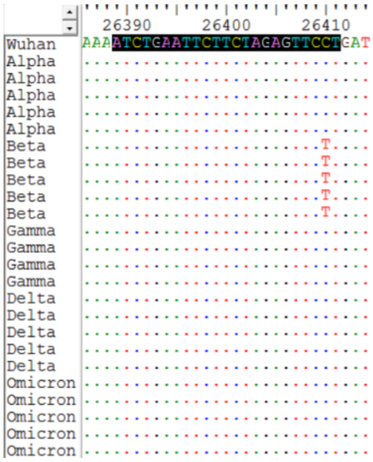

Target: N gene

Oligo: FIP-E-2

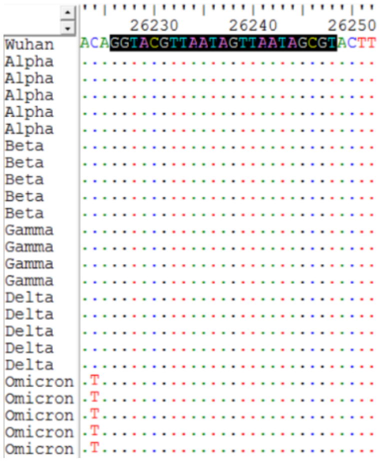

Target: N gene

Oligo: BIP-E-2 (anti-sense)

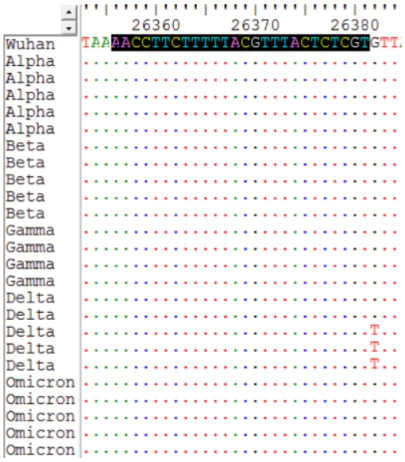

Target: N gene

Oligo: LF-E-2 (anti-sense)

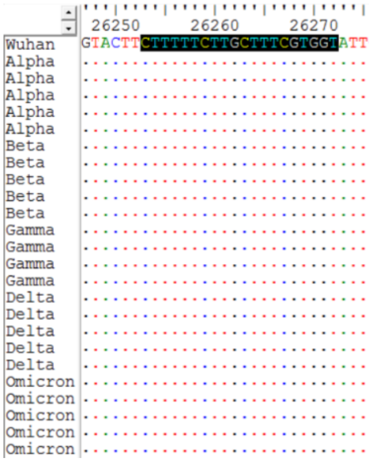

Target: N gene

Oligo: LB-E-1

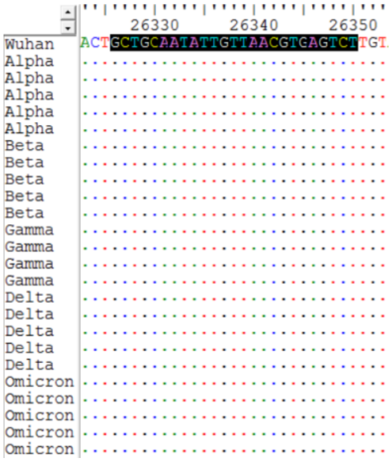

Target: E gene
Oligo: LbCas12a (anti-sense)

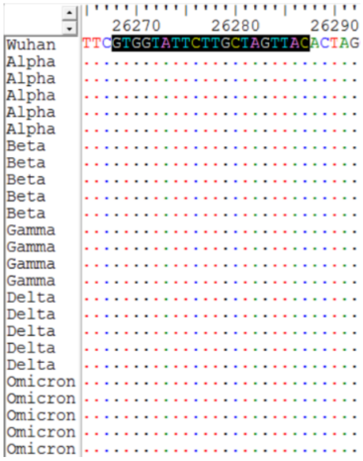

Target: E gene
Oligo: Cas12b (anti-sense)

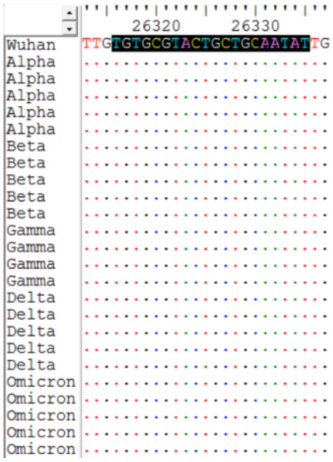

Title: Isothermal Amplification and Ambient Visualization in a Single Tube for the Detection of SARS-CoV-2 Using Loop-Mediated Amplification and CRISPR Technology

Target: N gene

Oligo: N-gene F3

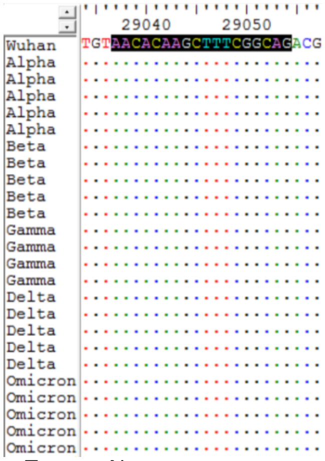

Target: N gene

Oligo: N-gene B3

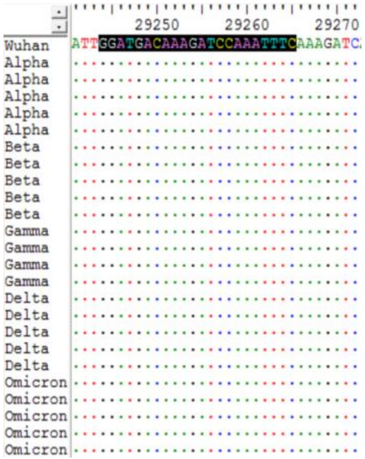

Target: N gene

Oligo: N-gene FIP

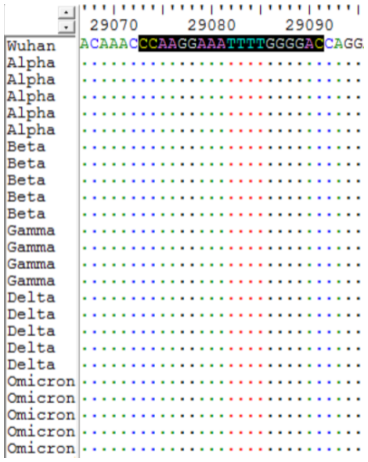

Target: N gene

Oligo: N-gene BIP

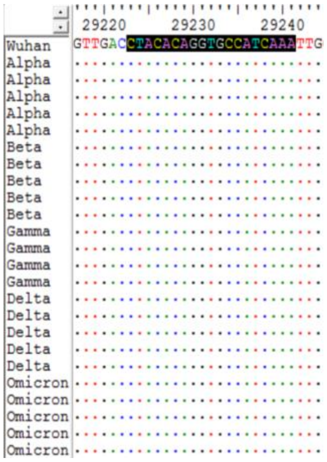

Target: N gene

Oligo: N gene-LF (anti-sense)

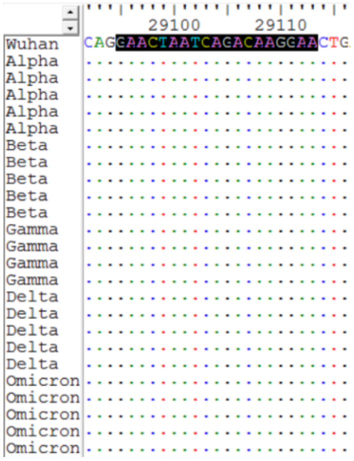

Target: N gene

Oligo: N gene-LB

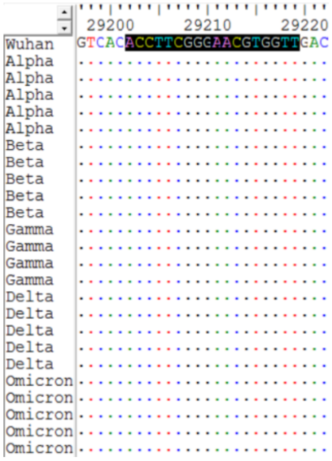

Target: E gene

Oligo: E gene_F3

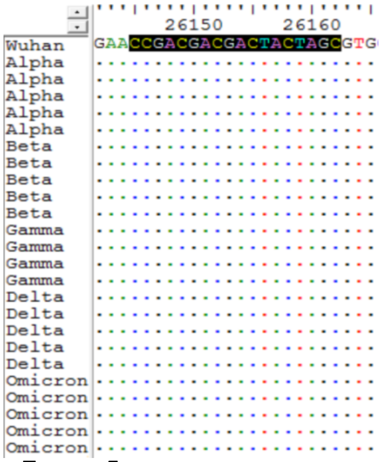

Target: E gene

Oligo: E gene_B3 (anti-sense)

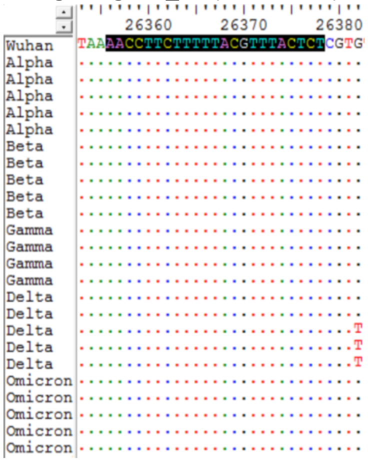

Target: E gene

Oligo: E gene_FIP

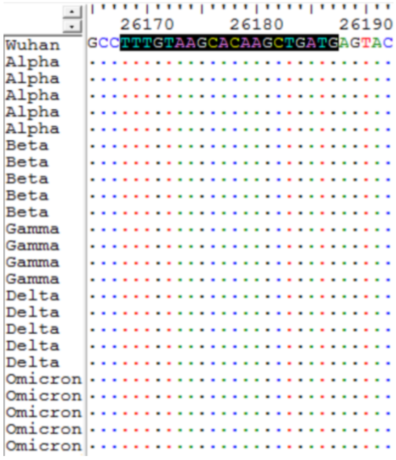

Target: E gene

Oligo: E gene_BIP (anti-sense)

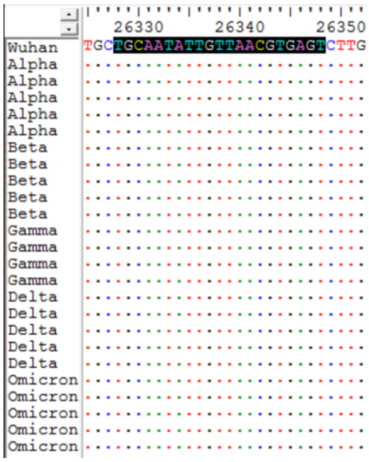

Target: E gene

Oligo: E gene_LF (anti-sense)

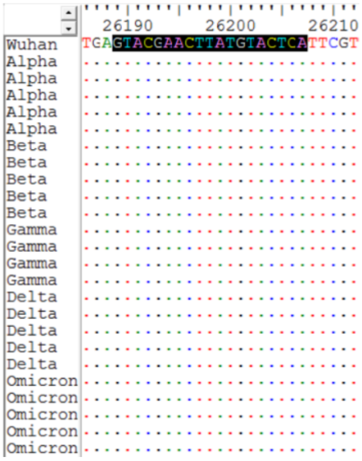

Target: E gene

Oligo: E gene_LB

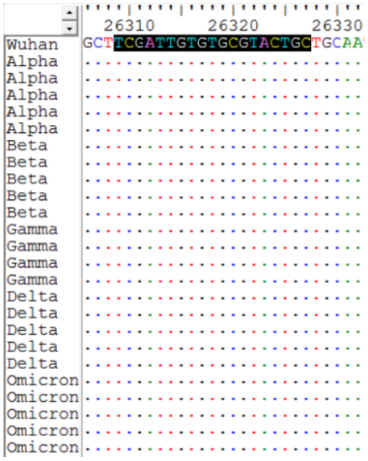

Target: N gene

Oligo: gRNA for the N gene

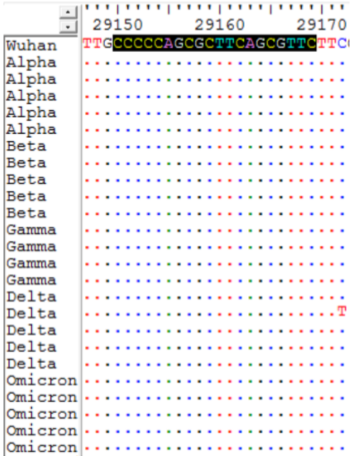

Target: E gene

Oligo: gRNA for the E gene

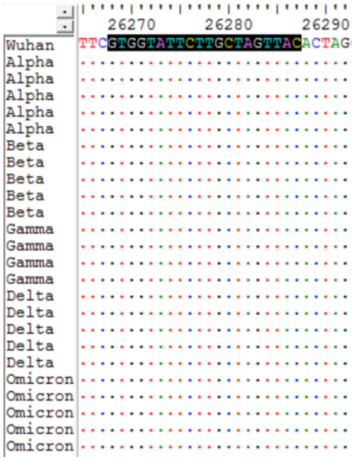

Title: MeCas12a, a Highly Sensitive and Specific System for COVID-19 Detection

Target: E gene

Oligo: SC2-E-RPA-F

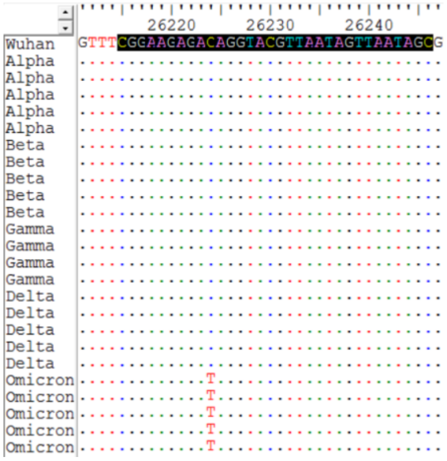

Target: E gene

Oligo: SC2-E-RPA-R (anti-sense)

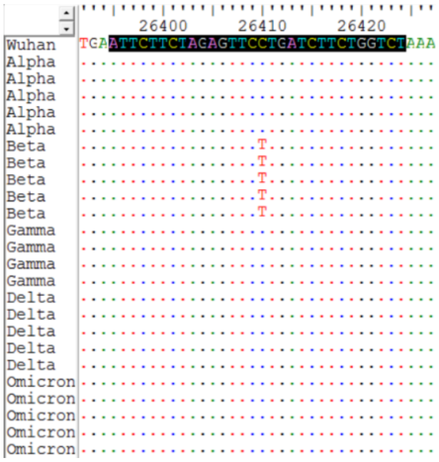

Target: E gene

Oligo: SC2-E-IVT-F

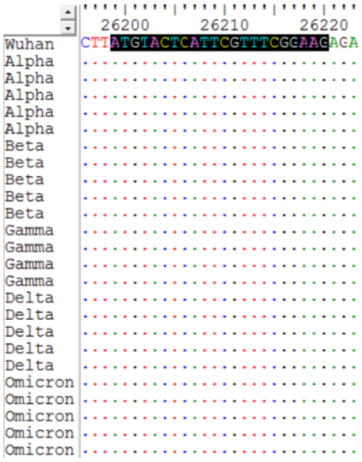

Title: Minimally instrumented SHERLOCK (miSHERLOCK) for CRISPR-based point-of-care diagnosis of SARS-CoV-2 and emerging variants

Target: N gene

Oligo: SARS-CoV-2 Nucleoprotein Forward Primer

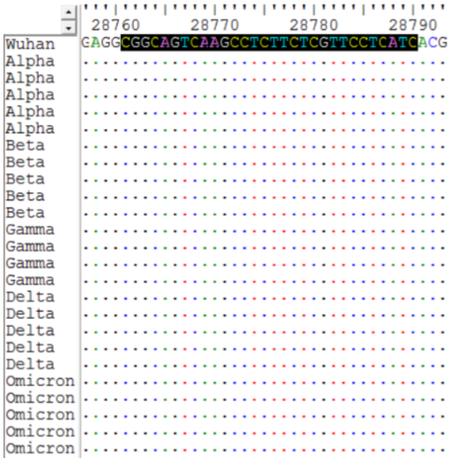

Target: N gene

Oligo: SARS-CoV-2 Nucleoprotein Reverse Primer (anti-sense)

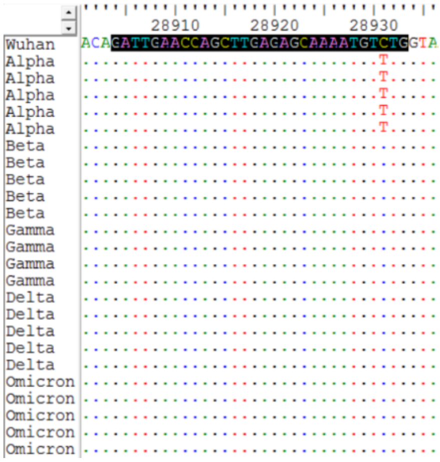

Target: N gene

Oligo: Nucleoprotein crRNA (anti-sense)

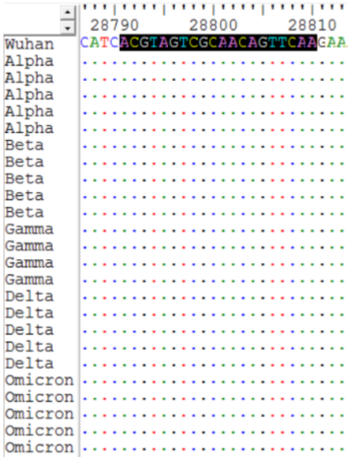

Title: One-tube SARS-CoV-2 detection platform based on RT-RPA and CRISPR/Cas12a

Target: RdRp gene

Oligo: forward primer with T7 promoter

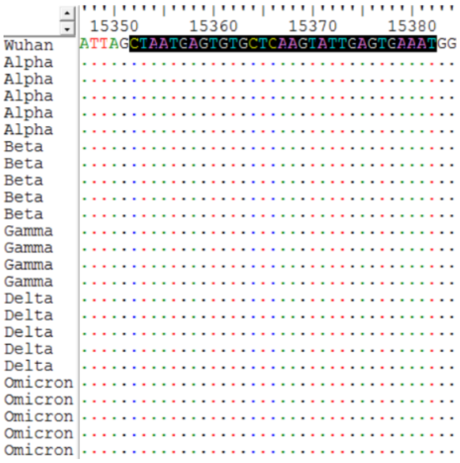

Target: RdRp gene

Oligo: reverse primer (anti-sense)

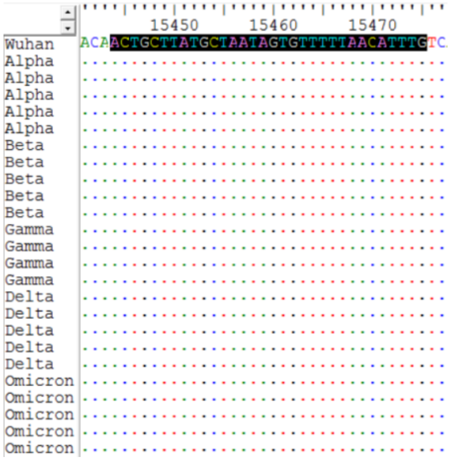

Target: N gene

Oligo: forward primer with T7 promoter

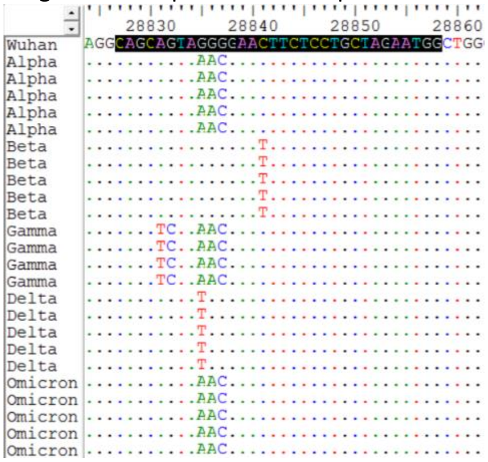

Target: N gene

Oligo: reverse primer (anti-sense)

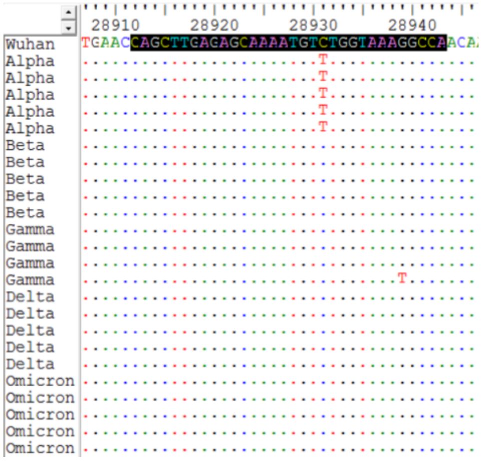

Target: RdRp gene

Oligo: crRNA for RdRp gene (anti-sense)

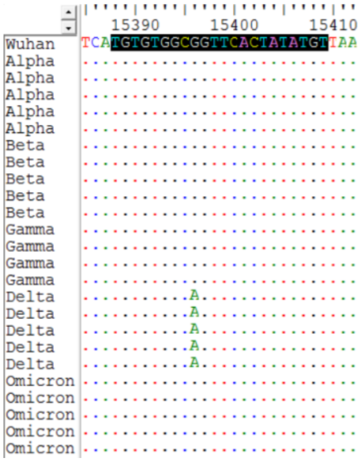

Target: N gene

Oligo: crRNA for N gene

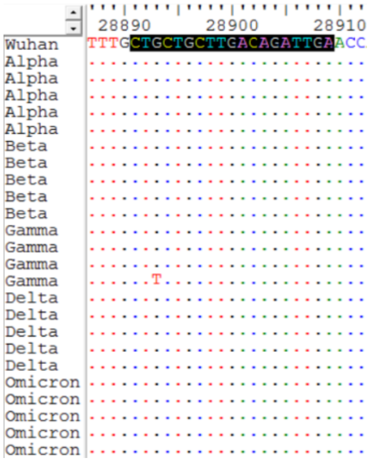

Title: Point-of-care CRISPR-Cas-assisted SARS-CoV-2 detection in an automated and portable droplet magnetofluidic device

Target: N gene

Oligo: RPA Forward primer

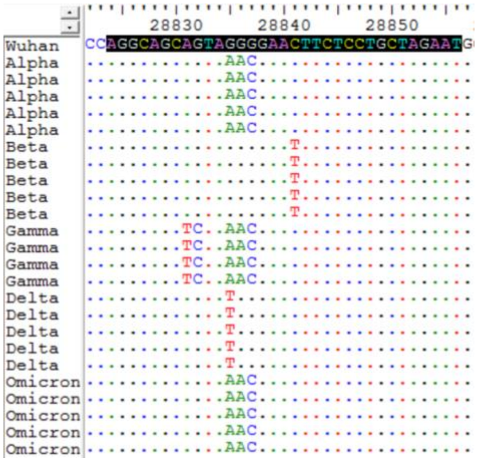

Target: N gene

Oligo: RPA Reverse primer (anti-sense)

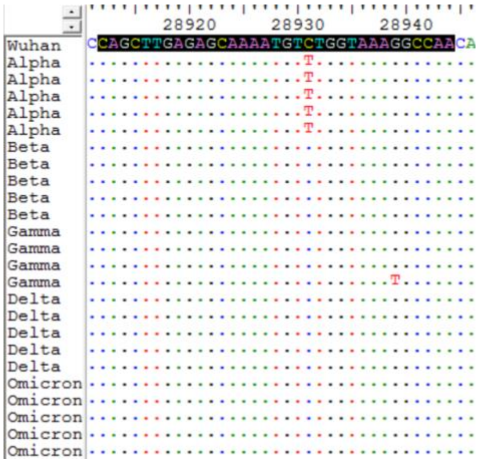

Target: N gene

Oligo: Cas12a-guide RNA1 (anti-sense)

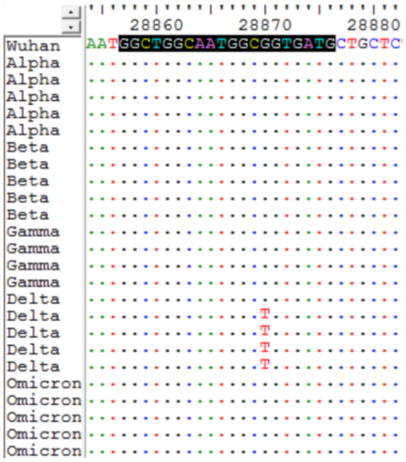

Target: N gene

Oligo: Cas12a-guide RNA2

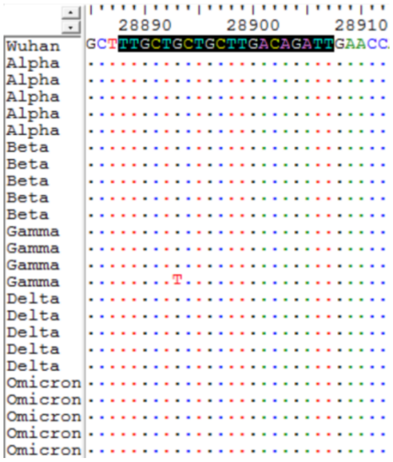

Title: Point-of-care testing for COVID-19 using SHERLOCK diagnostics

Target: N gene

Oligo: F3

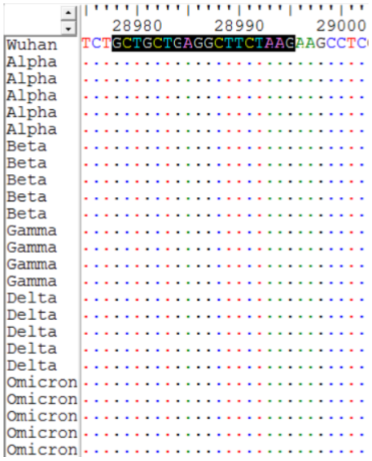

Target: N gene

Oligo: B3 (anti-sense)

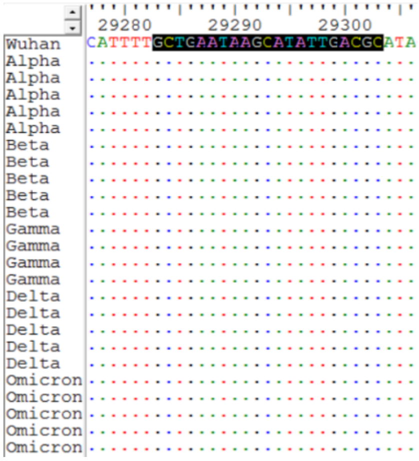

Target: N gene

Oligo: FIP

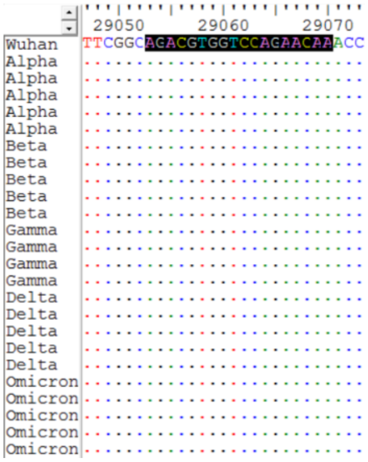

Target: N gene

Oligo: BIP (anti-sense)

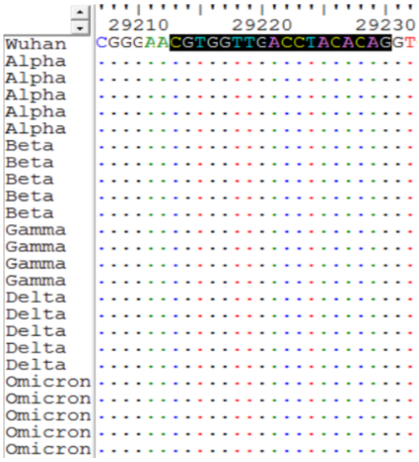

Target: N gene

Oligo: Loop Forward (anti-sense)

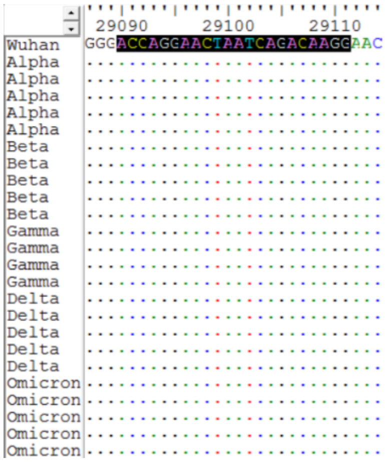

Target: N gene

Oligo: Loop Reverse

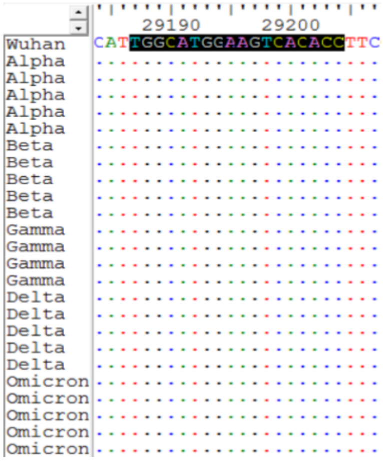

Target: N gene

Oligo: AapCas12b Guide RNA

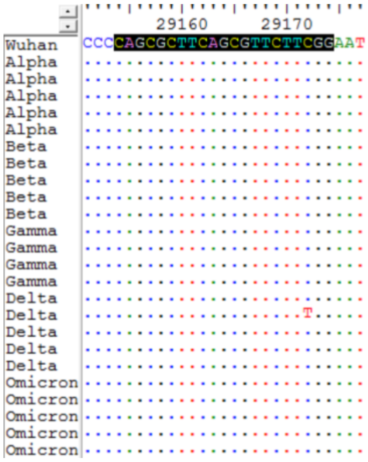

Title: Rapid and sensitive detection of COVID-19 using CRISPR/Cas12a-based detection with naked eye readout, CRISPR/Cas12a-NER

Target: Orf1a
Oligo: orf1a-RPA-F

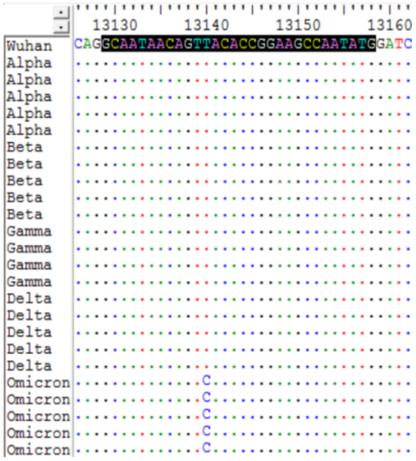

Target: Orf1a
Oligo: orf1a-RPA-R

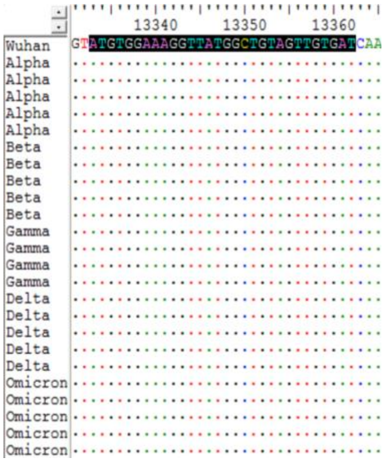

Target: Orf1b
Oligo: orf1b-RPA-F

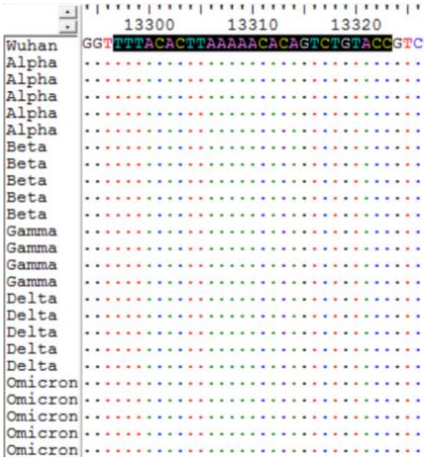

Target: Orf1b
Oligo: orf1b-RPA-R

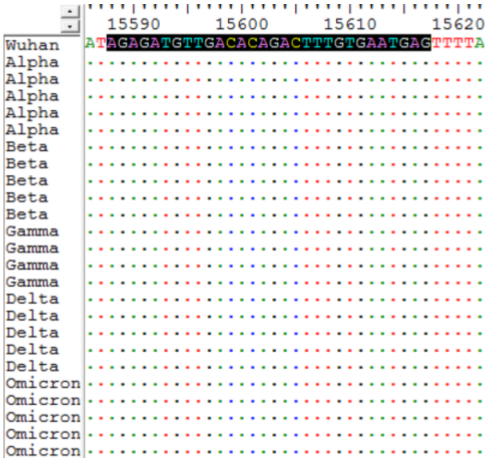

Target: E gene
Oligo: E-RPA-F

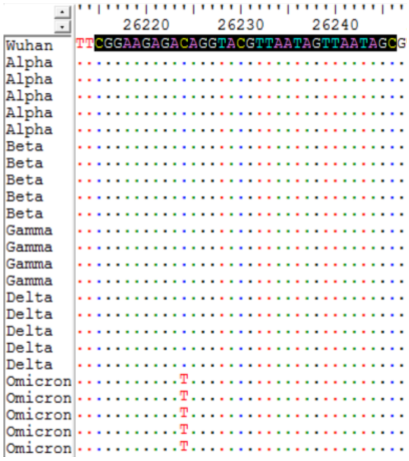

Target: E gene
Oligo: E-RPA-R (anti-sense)

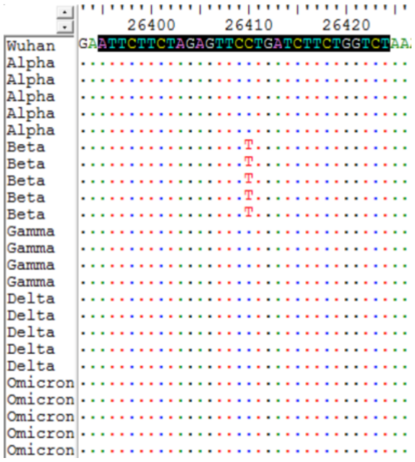

Target: N gene
Oligo: N-RPA-F

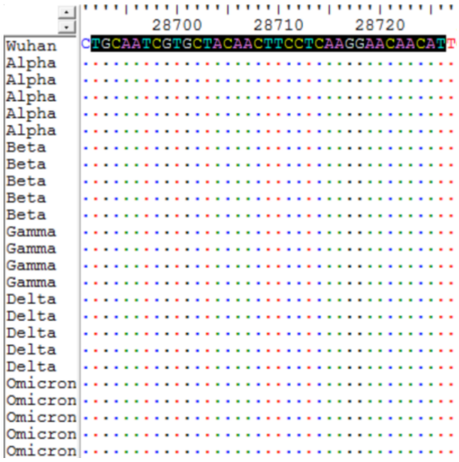

Target: N gene
Oligo: N-RPA-R

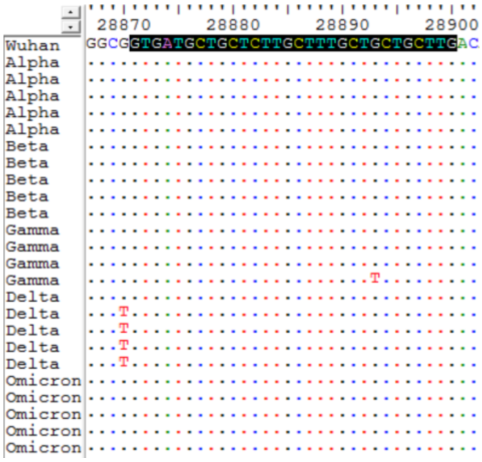

Target: Orf1a
Oligo: orf1a-crRNA1

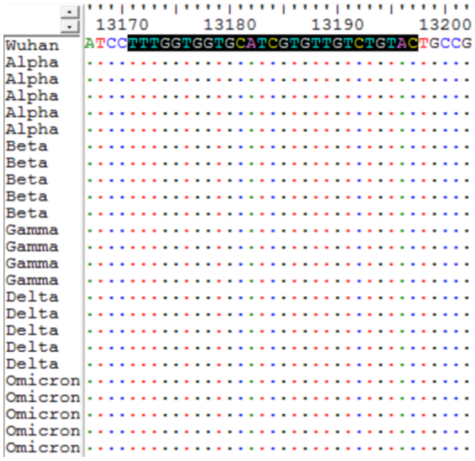

Target: Orf1a
Oligo: orf1a-crRNA2

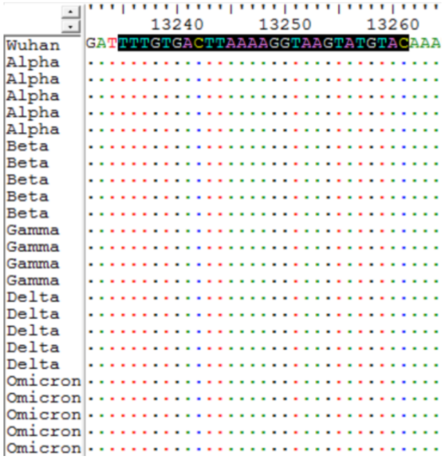

Target: Orf1a
Oligo: orf1a-crRNA3 (anti-sense)

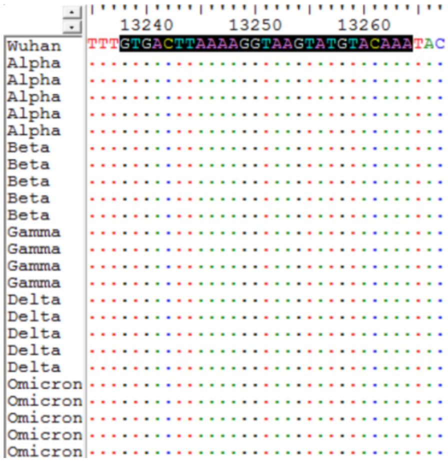

Target: Orf1a
Oligo: orf1a-crRNA4 (anti-sense)

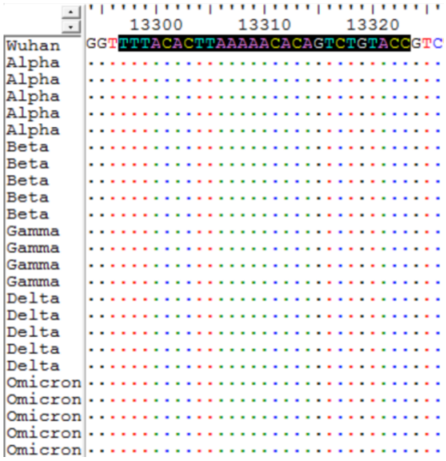

Target: Orf1b

Oligo: orf1b-crRNA1 (anti-sense)

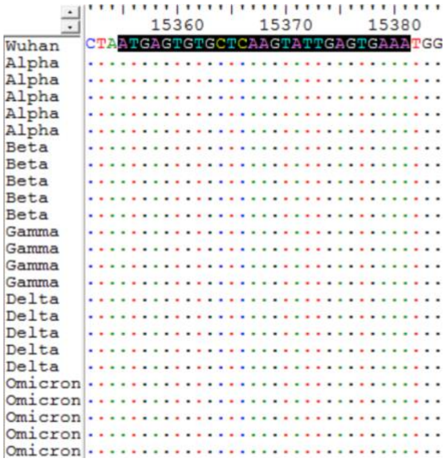

Target: Orf1b

Oligo: orf1b-crRNA2

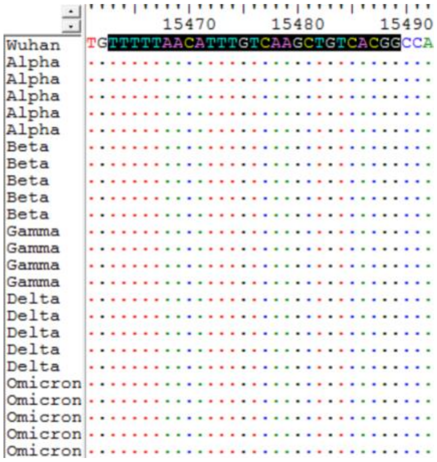

Target: Orf1b

Oligo: orf1b-crRNA3

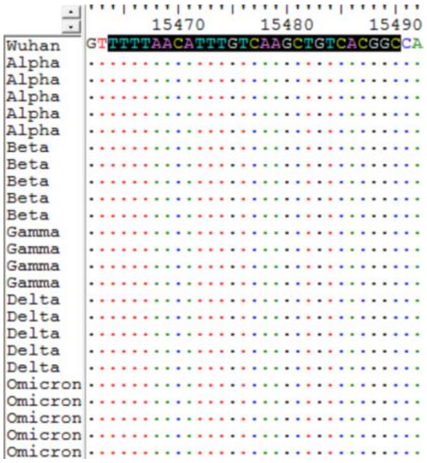

Target: Orf1b

Oligo: orf1b-crRNA4

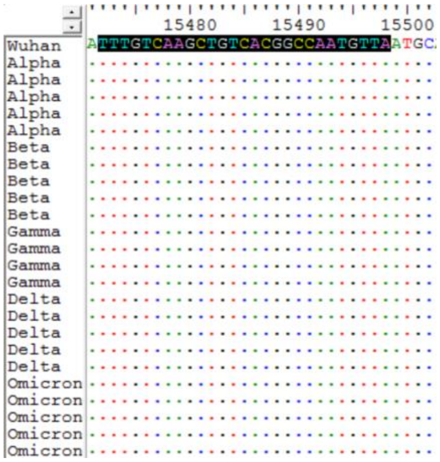

Target: E gene

Oligo: E-crRNA1

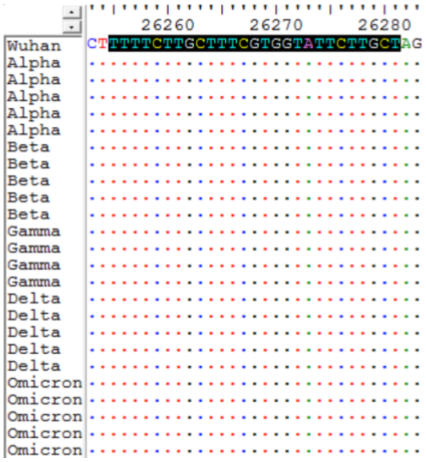

Target: E gene

Oligo: E-crRNA2

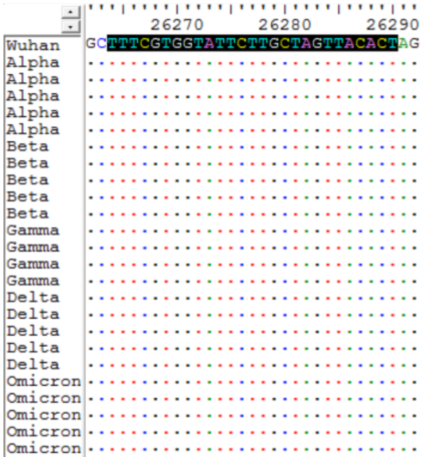

Target: E gene

Oligo: E-crRNA3 (anti-sense)

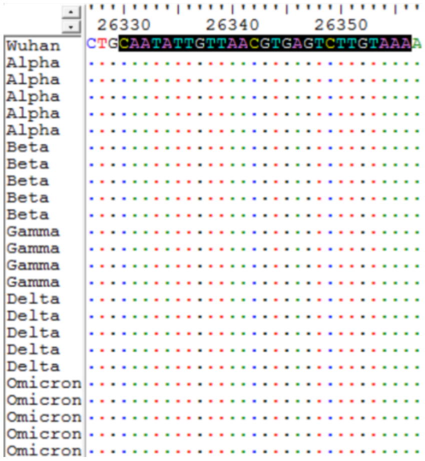

Target: E gene

Oligo: E-crRNA4

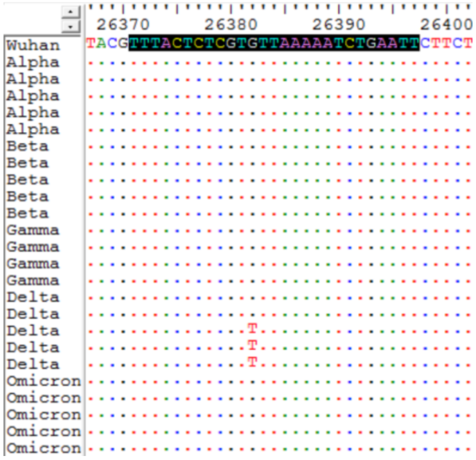

Target: N gene

Oligo: N-crRNA1 (anti-sense)

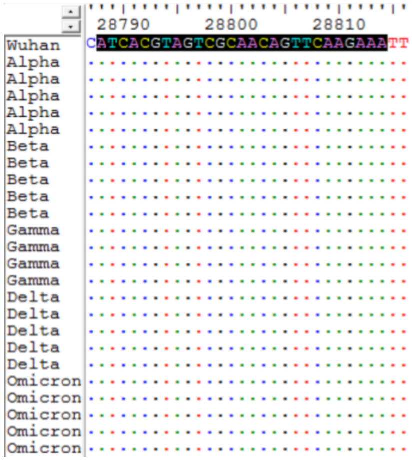

Target: N gene

Oligo: N-crRNA2

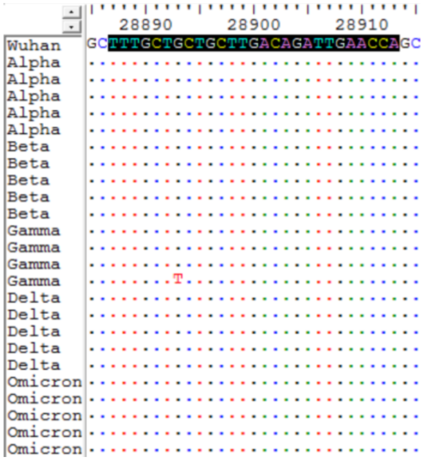

Target: N gene

Oligo: N-crRNA3 (anti-sense)

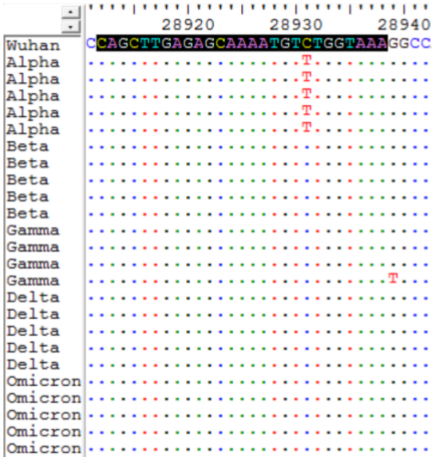

Title: Rapid and Sensitive Detection of SARS-CoV-2 Using Clustered Regularly Interspaced Short Palindromic Repeats

Target: M gene

Oligo: COVID19 M-RPAF

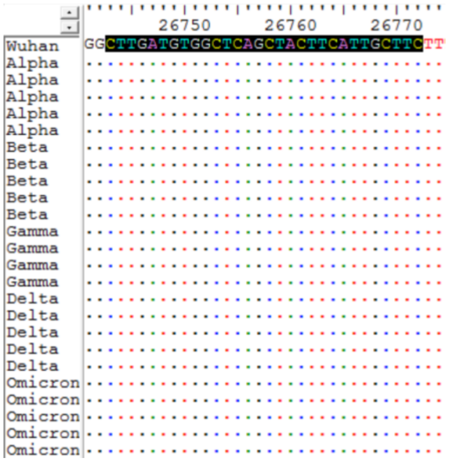

Target: M gene

Oligo: COVID19 M-RPAR

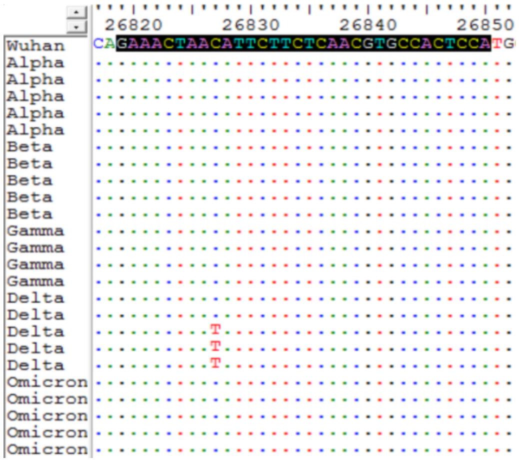

Target: N gene

Oligo: COVID19 N2-RPAF

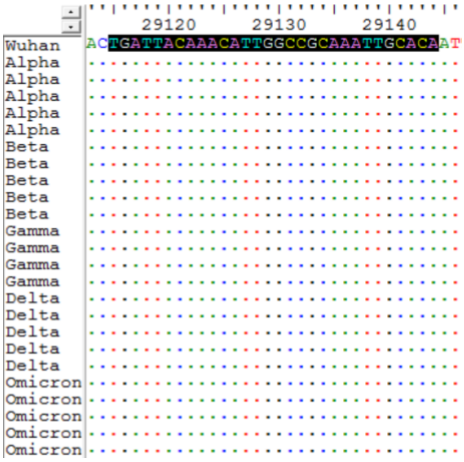

Target: N gene

Oligo: COVID19 N2-RPAR

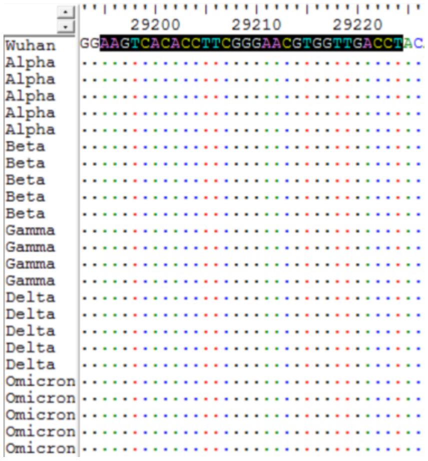

Target: S gene

Oligo: COVID19 S2-RPAF

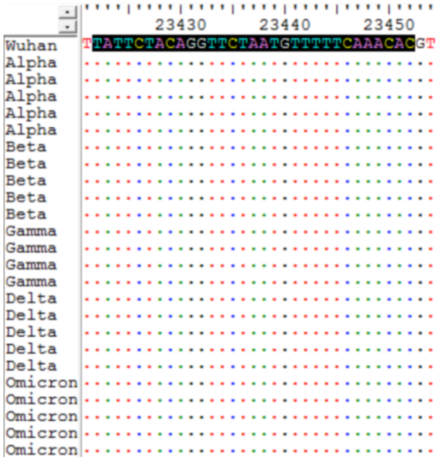

Target: S gene

Oligo: COVID19 S2-RPAR

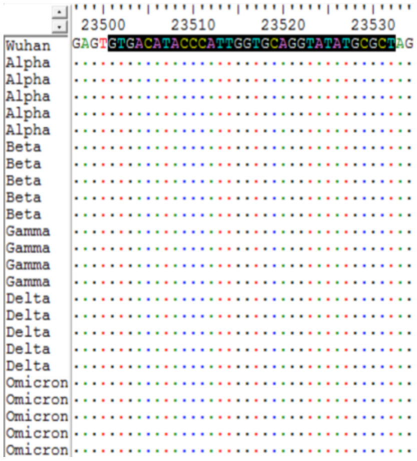

Target: M gene

Oligo: COVID19 M gRNA

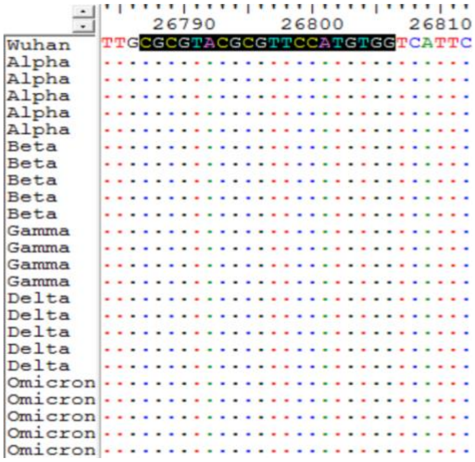

Target: N gene

Oligo: COVID19 N2 gRNA

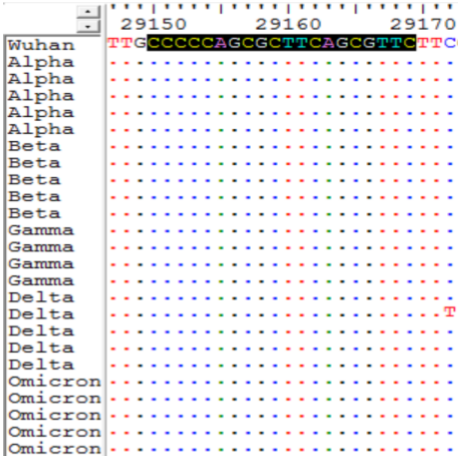

Target: S gene

Oligo: COVID19 S2 gRNA

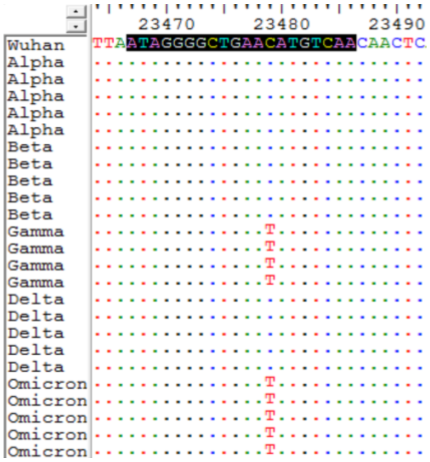

Title: Rapid Detection of 2019 Novel Coronavirus SARS-CoV-2 Using a CRISPR-based DETECTR Lateral Flow Assay

Target: E gene
Oligo: E-Sarbeco_F1_RPA

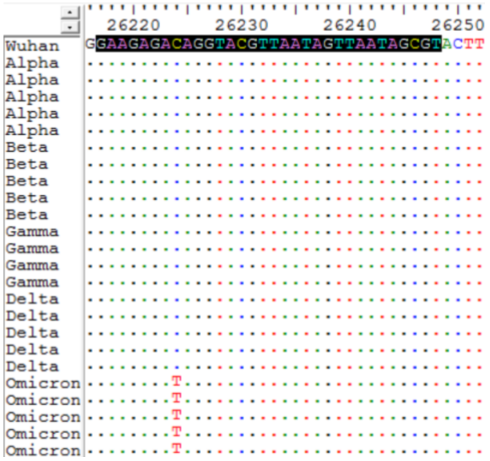

Target: E gene
Oligo: E-Sarbeco-R2_RPA

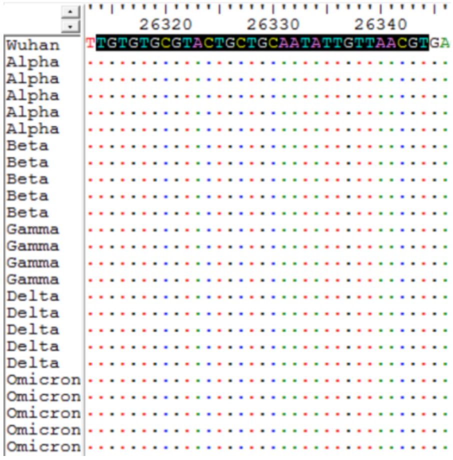

Target: N gene
Oligo: 2019-nCoV_N2-F_RPA

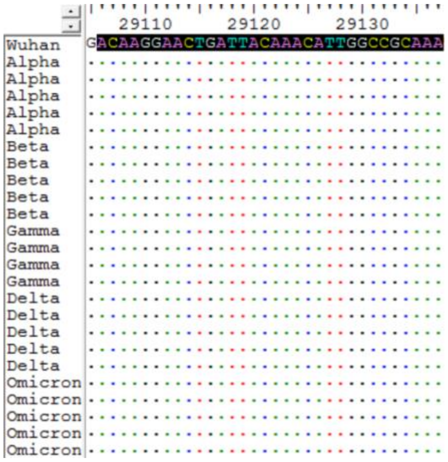

Target: N gene
Oligo: 2019-nCoV_N2-R_RPA

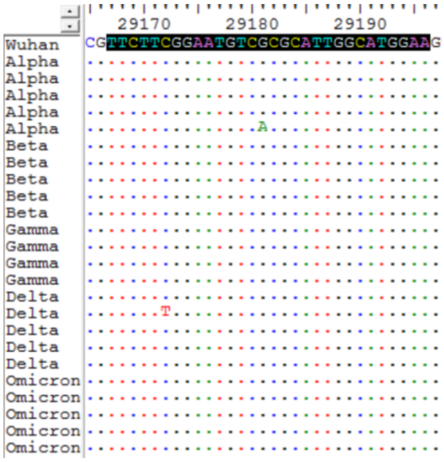

Target: N gene
Oligo: N-gene F3

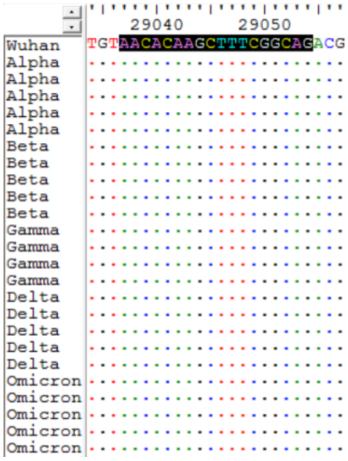

Target: N gene
Oligo: N-gene B3

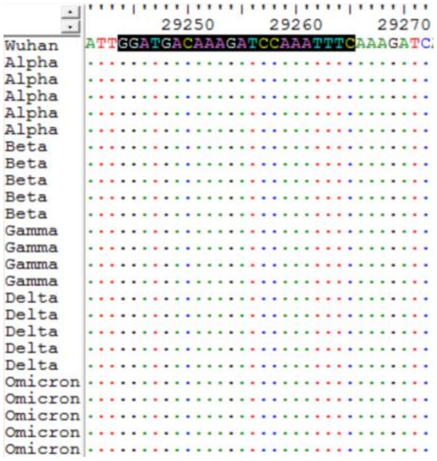

Target: N gene
Oligo: N-gene FIP

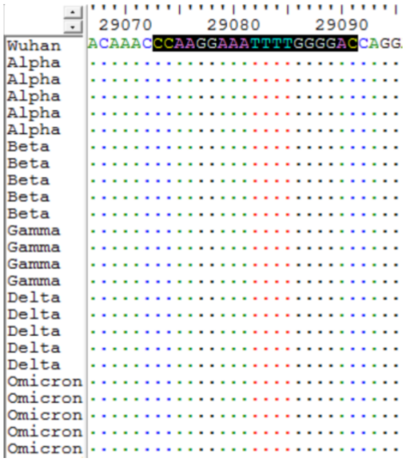

Target: N gene
Oligo: N-gene BIP

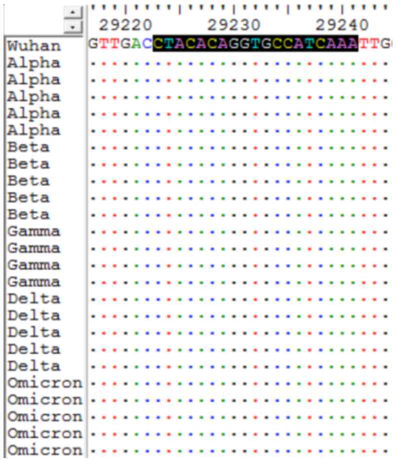

Target: N gene
Oligo: N-gene-LF (anti-sense)

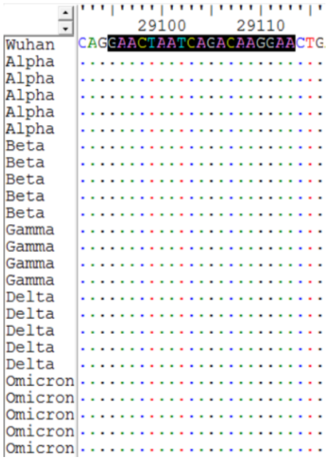

Target: N gene
Oligo: N-gene-LB

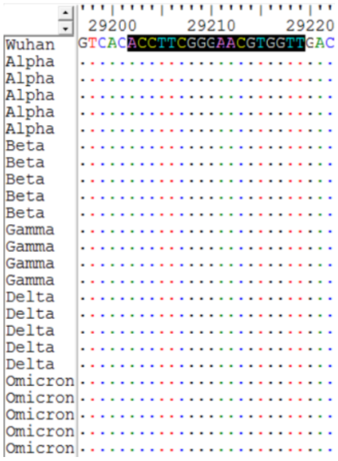

Target: E gene
Oligo: E-gene F3

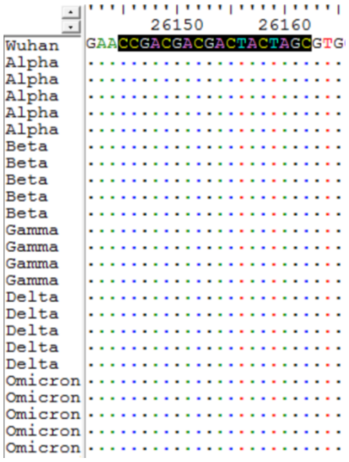

Target: E gene
Oligo: E-gene B3 (anti-sense)

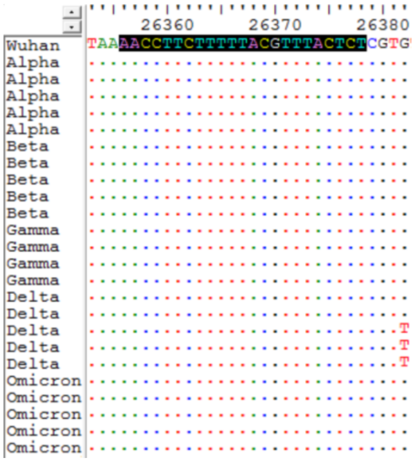

Target: E gene
Oligo: E-gene FIP

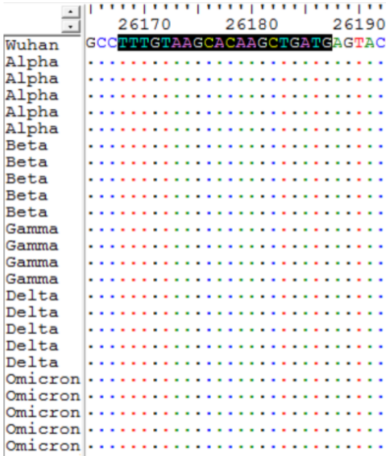

Target: E gene
Oligo: E-gene BIP (anti-sense)

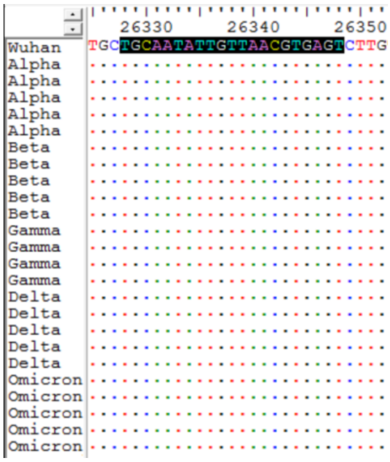

Target: E gene
Oligo: E-gene FL (anti-sense)

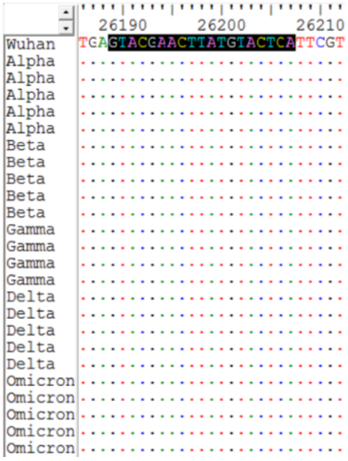

Target: E gene
Oligo: E-gene BL

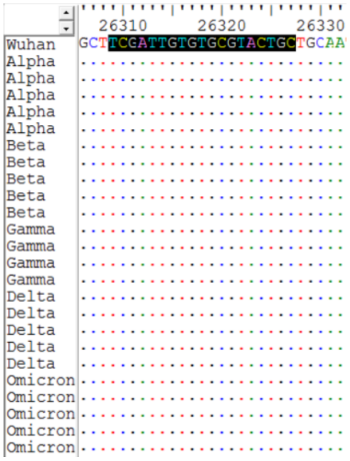

Target: E gene
Oligo: E-gene gRNA

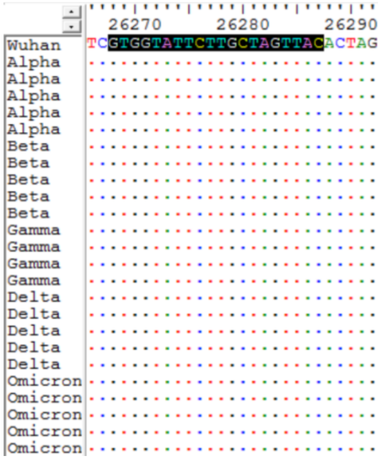

Target: N gene
Oligo: N-gene gRNA

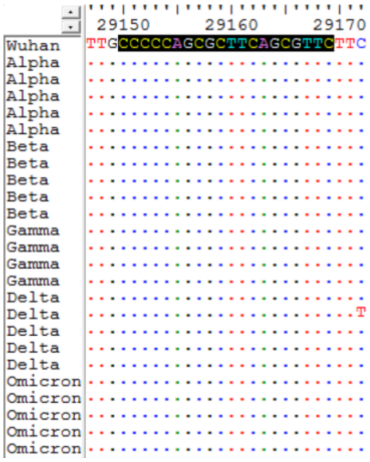

Title: Rapid detection of SARS-CoV-2 with CRISPR-Cas12a

Target: Orf1ab

Oligo: ORF1ab-F5

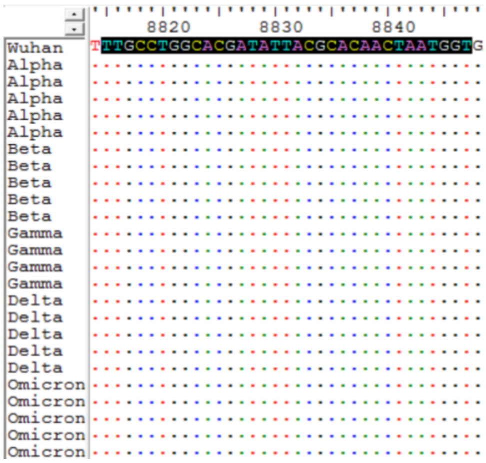

Target: Orf1ab

Oligo: ORF1ab-R1 (anti-sense)

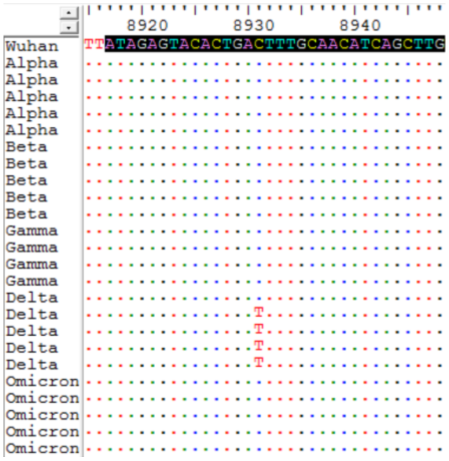

Target: N gene

Oligo: N-F4

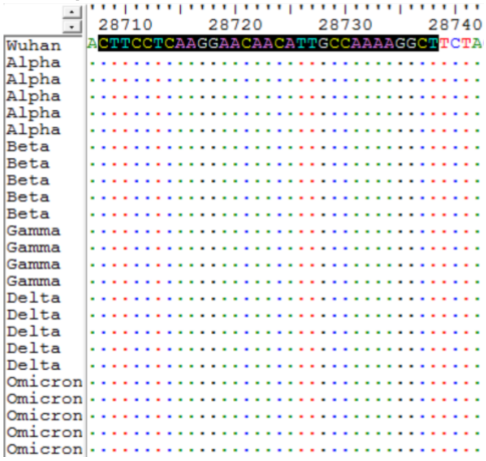

Target: N gene

Oligo: N-R2 (anti-sense)

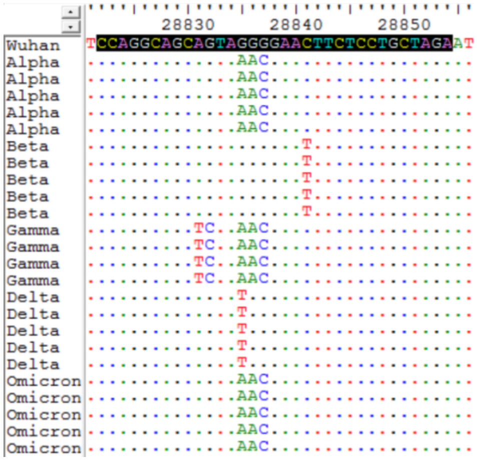

Target: Orf1ab

Oligo: crRNA-ORF1ab

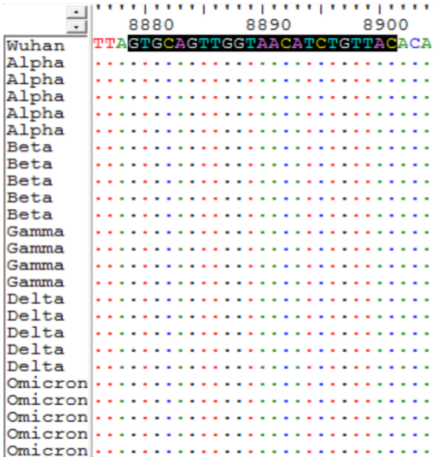

Target: N gene

Oligo: crRNA-nCOV-N (anti-sense)

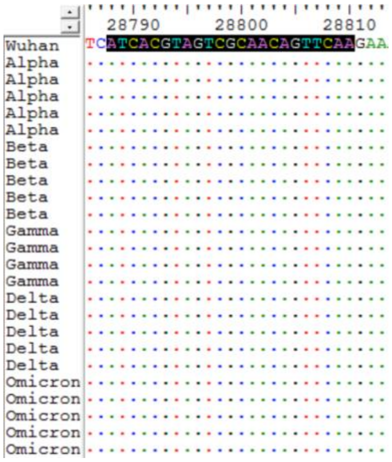

Title: Rapid SARS-CoV-2 testing in primary material based on a novel multiplex RT-LAMP assay

Target: Orf1a
Oligo: ORF1a-A-F3

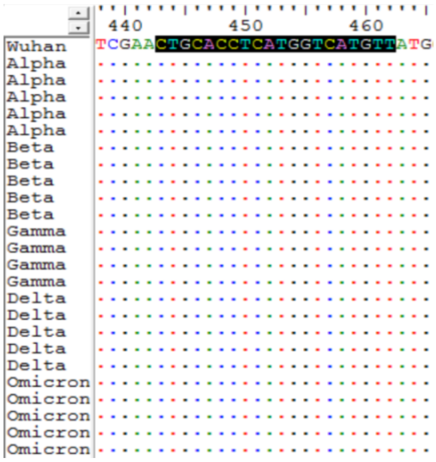

Target: Orf1a
Oligo: ORF1a-A-B3 (anti-sense)

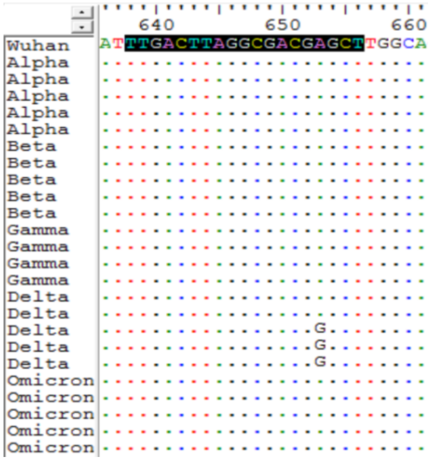

Target: Orf1a
Oligo: ORF1a-A-FIP

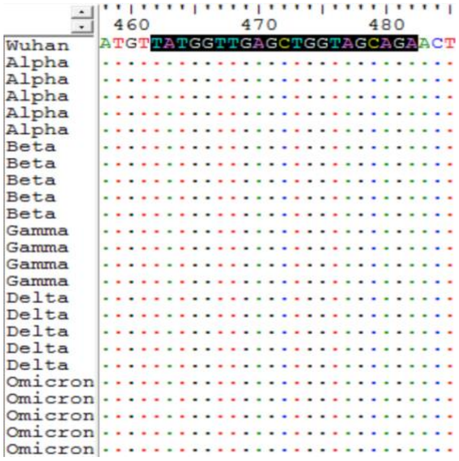

Target: Orf1a
Oligo: ORF1a-A-BIP (anti-sense)

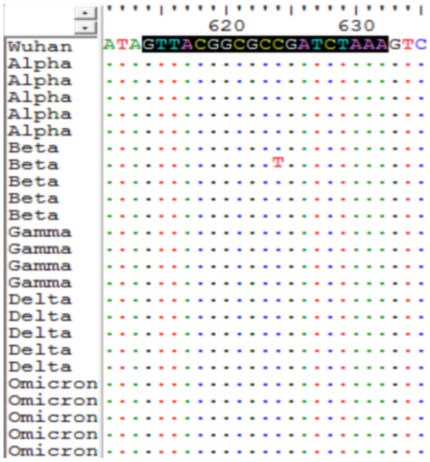

Target: Orf1a
Oligo: ORF1a-A-LF (anti-sense)

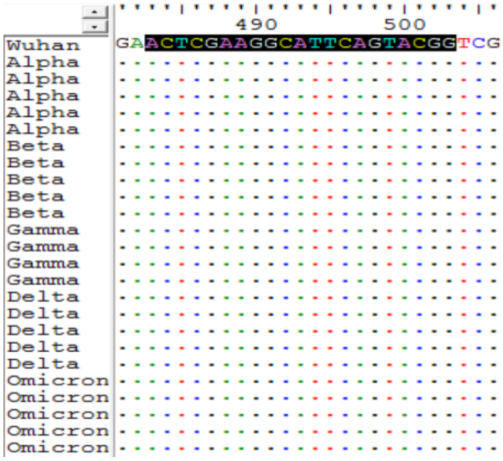

Target: Orf1a
Oligo: ORF1a-A-LB

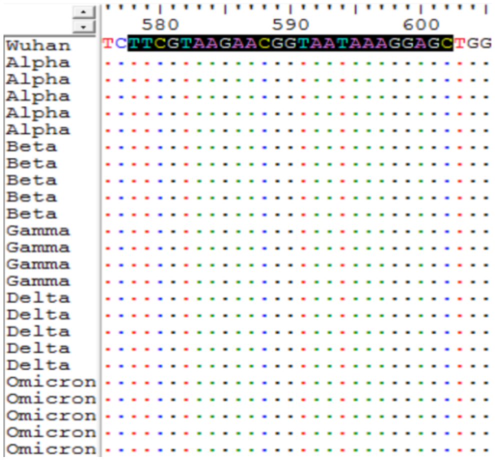

Target: N gene

Oligo: GeneN-A-F3

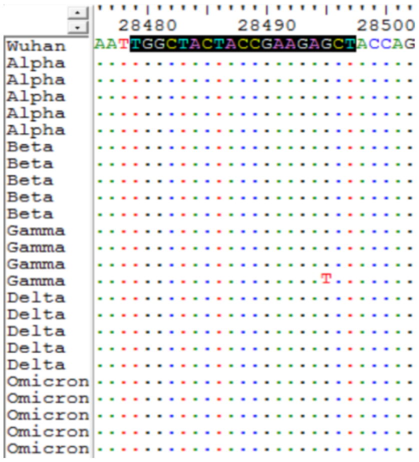

Target: N gene

Oligo: Gene N-A-FIP

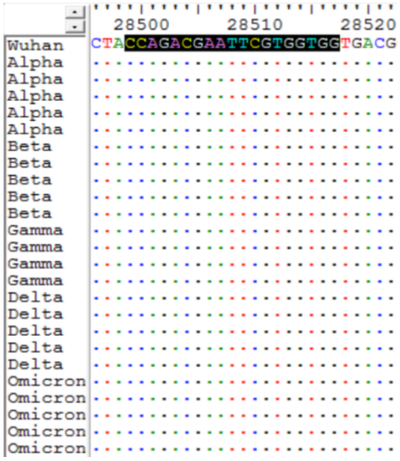

Target: N gene

Oligo: Gene N-A-LF (anti-sense)

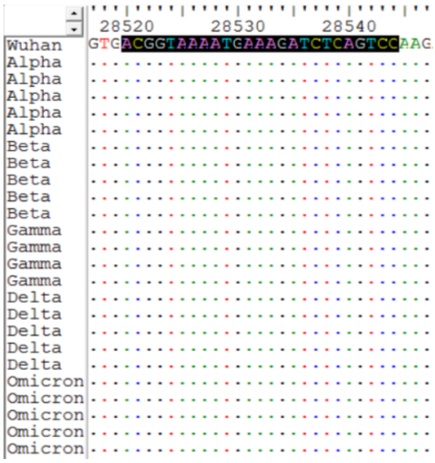

Target: N gene

Oligo: Gene N-A-B3 (anti-sense)

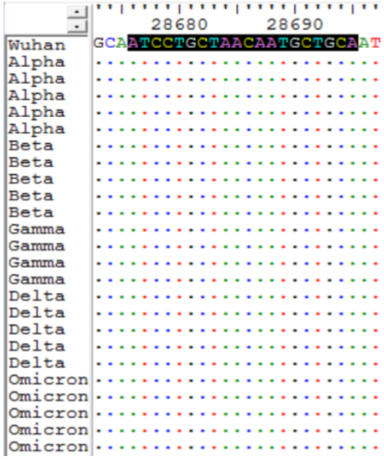

Target: N gene

Oligo: Gene N-A-BIP (anti-sense)

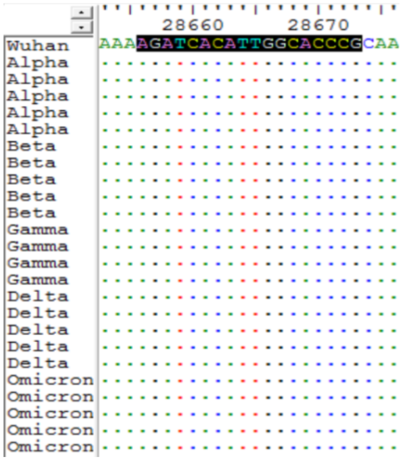

Target: N gene

Oligo: Gene N-A-LB

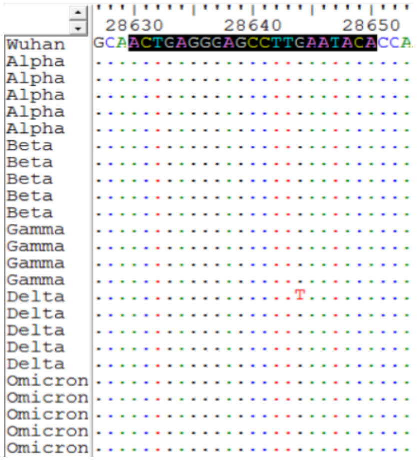

Target: ORF7a
Oligo: ORF7a-F3

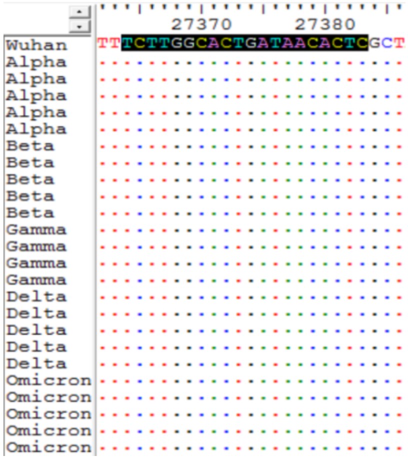

Target: ORF7a
Oligo: ORF7a-B3 (anti-sense)

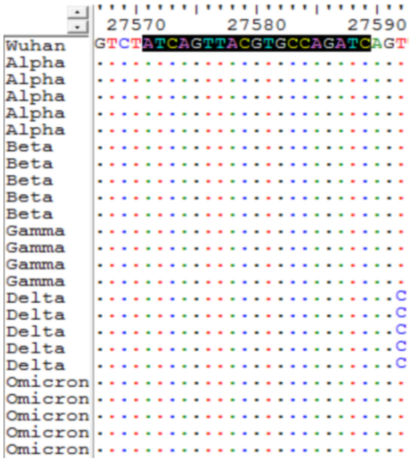

Target: ORF7a
Oligo: ORF7a-FIP

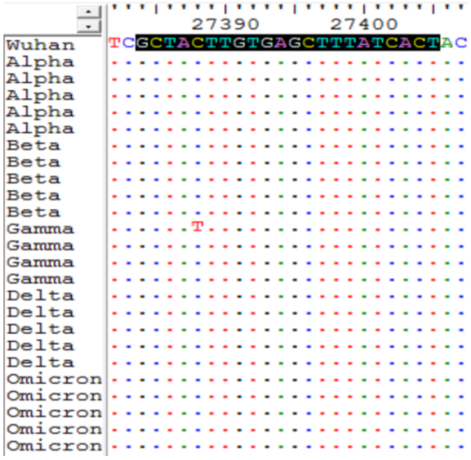

Target: ORF7a
Oligo: ORF7a-BIP (anti-sense)

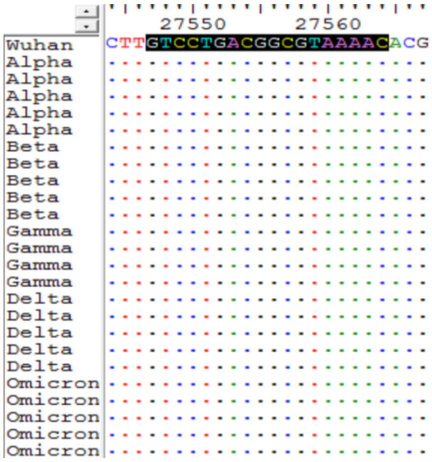

Target: ORF7a
Oligo: ORF7a-LF (anti-sense)

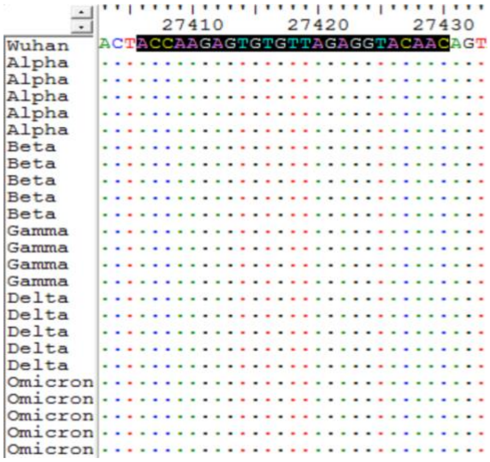

Target: ORF7a
Oligo: ORF7a-LB

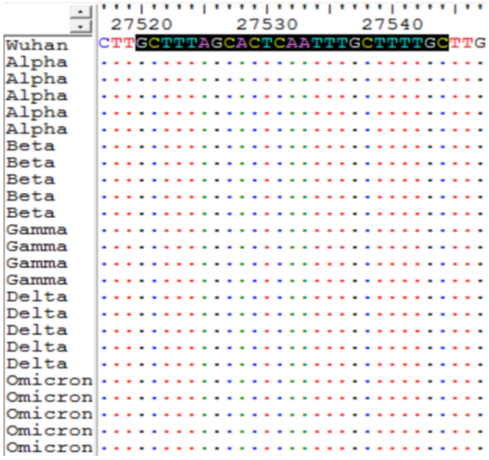

Target: ORF3a

Oligo: ORF3a-A-F3

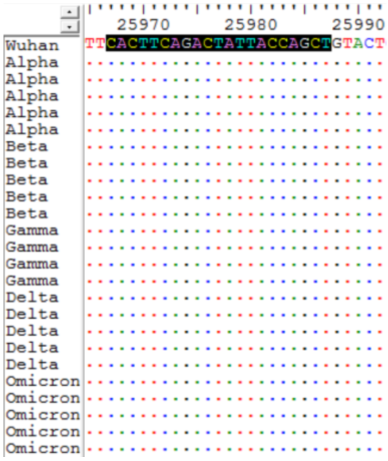

Target: ORF3a

Oligo: ORF3a-A-B3 (anti-sense)

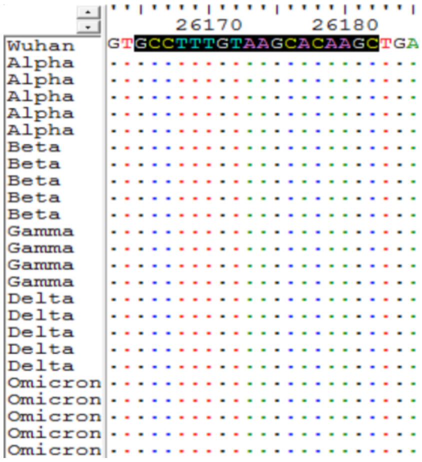

Target: ORF3a

Oligo: ORF3a-A-FIP

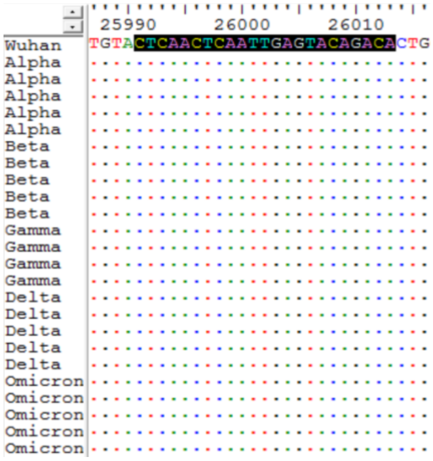

Target: ORF3a

Oligo: ORF3a-A-BIP (anti-sense)

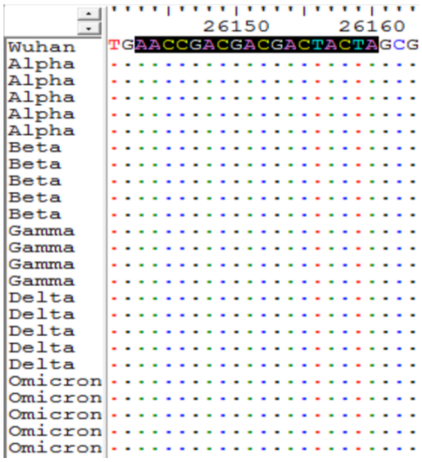

Target: ORF3a

Oligo: ORF3a-A-LF (anti-sense)

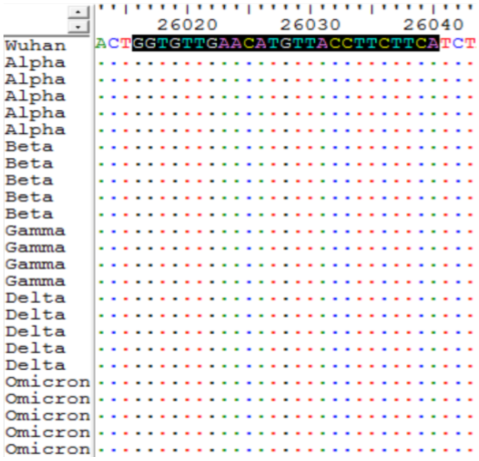

Target: ORF3a

Oligo: ORF3a-A-LB

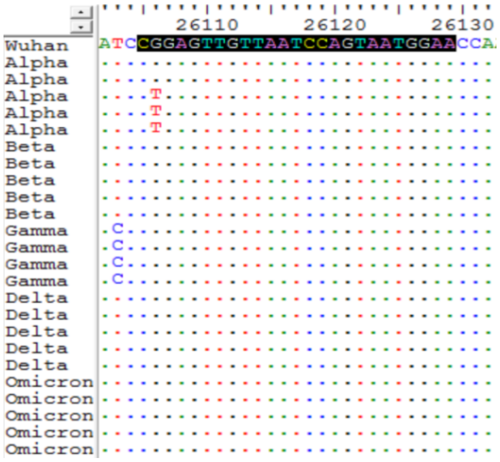

Target: Orf1a

Oligo: LwaCas13a crRNA ORF1ab2 (anti-sense)

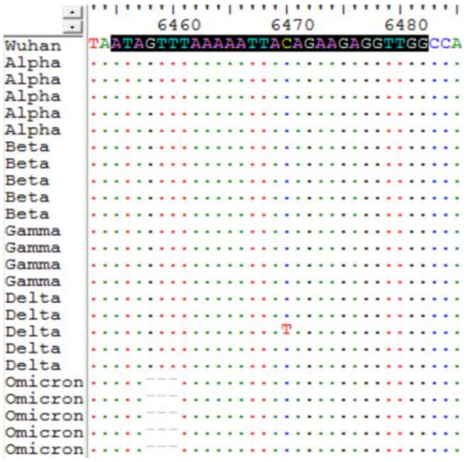

Target: N gene

Oligo: LwaCas13a crRNA Gene-N

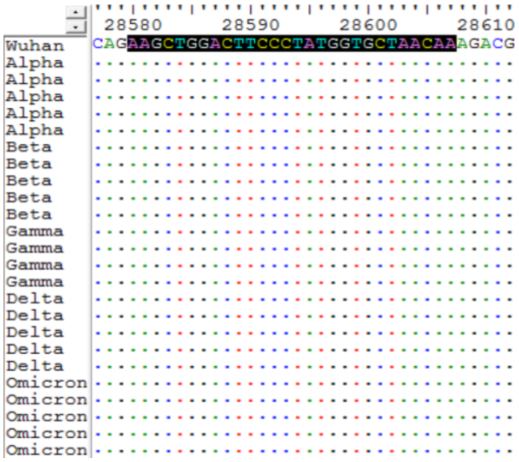

Target: ORF7a

Oligo: LwaCas13a crRNA Orf7 (anti-sense)

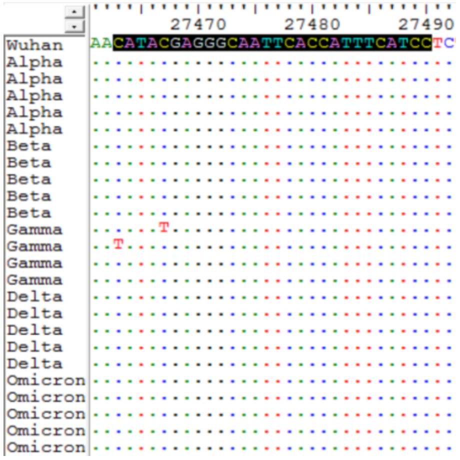

Target: ORF3a

Oligo: LwaCas13a crRNA Orf3 (anti-sense)

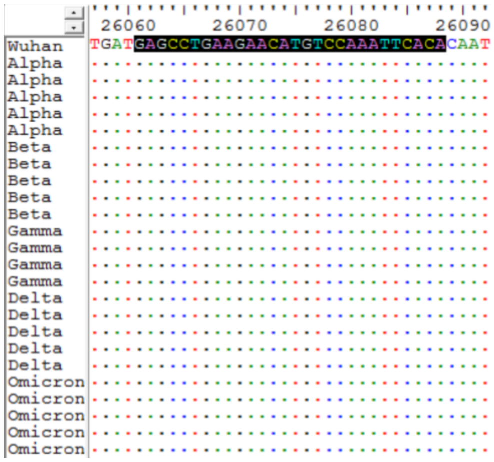

Target: Orf1ab

Oligo: Orf1ab-Fw_v12

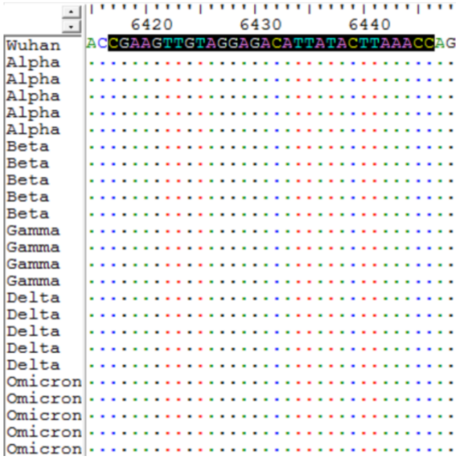

Target: Orf1ab

Oligo: Orf1ab-Rev_v12 (anti-sense)

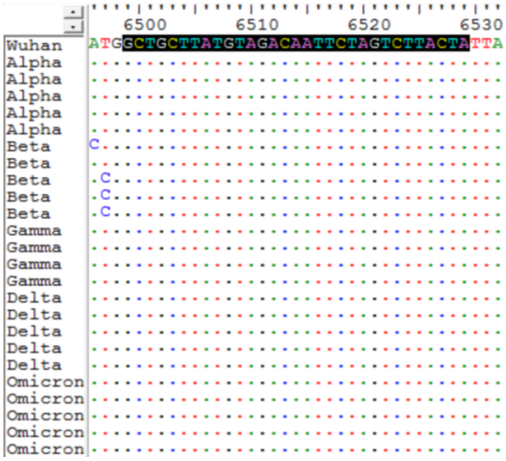

Target: S gene

Oligo: S-RPA-Fw_v12

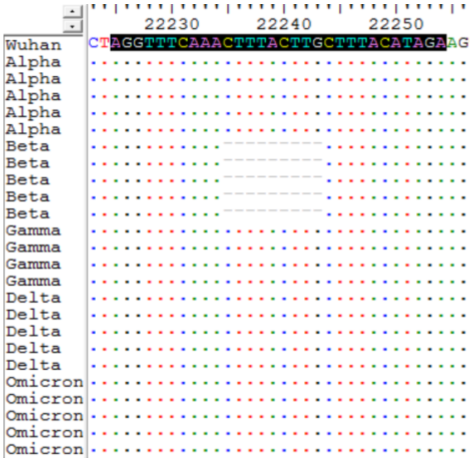

Target: S gene

Oligo: S-RPA-Rev_v12 (anti-sense)

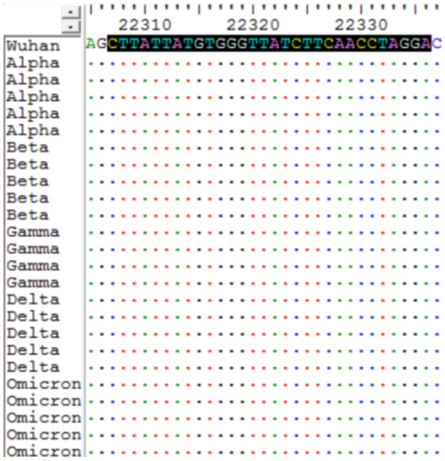

Target: Orf1ab

Oligo: LwaCas13a crRNA ORF1ab2 (anti-sense)

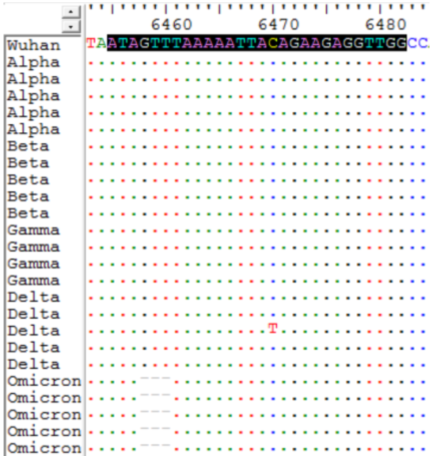

Target: S gene

Oligo: LwaCas13a crRNA S Gene2 (anti-sense)

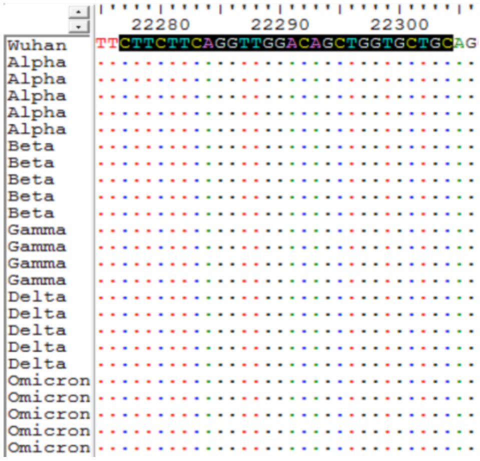

Title: Reverse Transcription Recombinase Polymerase Amplification Coupled with CRISPR-Cas12a for Facile and Highly Sensitive Colorimetric SARS-CoV-2 Detection

Target: Orf1ab
Oligo: Forward primer (OFR1ab)

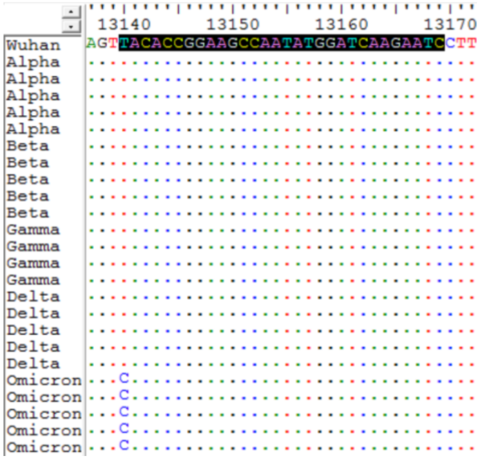

Target: Orf1ab
Oligo: Reverse primer (OFR1ab) (anti-sense)

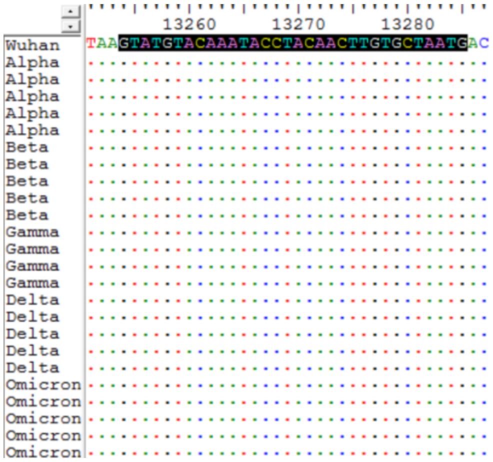

Target: N gene
Oligo: Forward primer (N region)

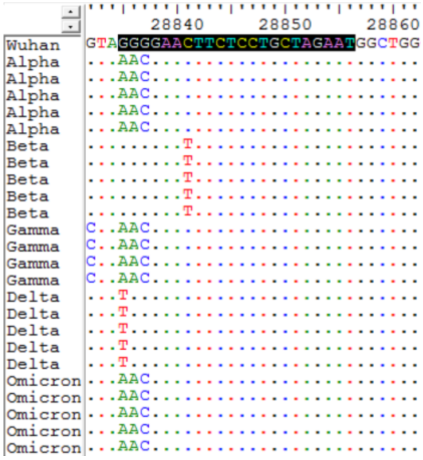

Target: N gene
Oligo: Reverse primer (N region) (anti-sense)

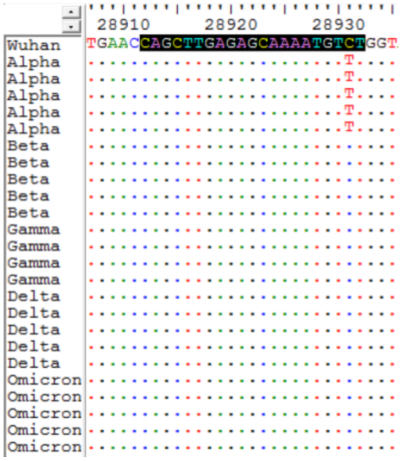

Target: Orf1ab
Oligo: crRNA (OFR1ab)

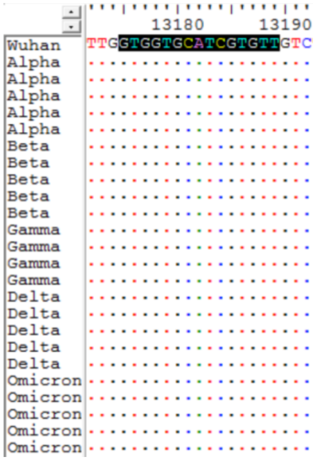

Target: N gene
Oligo: crRNA (N region)

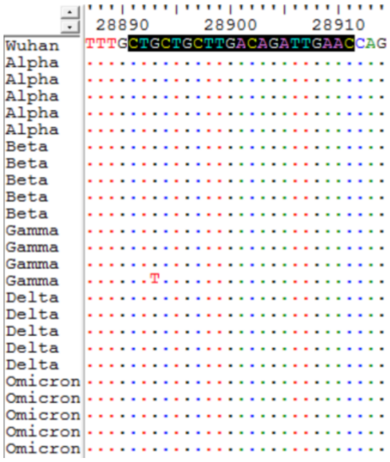

Title: SARS-CoV-2 detection with CRISPR diagnostics

Target: RdRp gene
Oligo: RPA-RdRp-F0

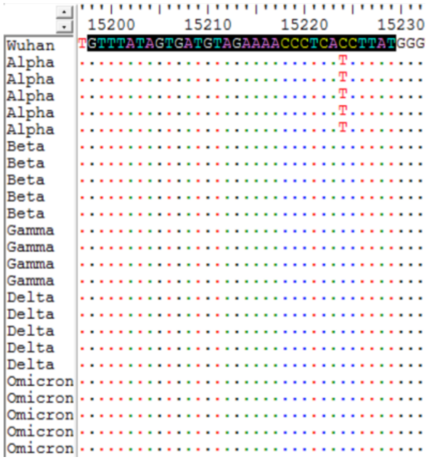

Target: RdRp gene
Oligo: RPA-RdRp-R2 (anti-sense)

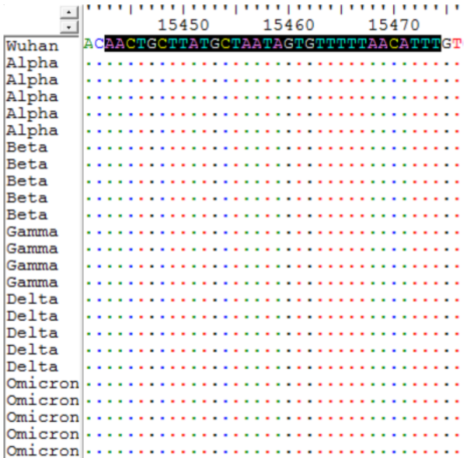

Target: RdRp gene
Oligo: AasgRNA-RdRp-3 (anti-sense)

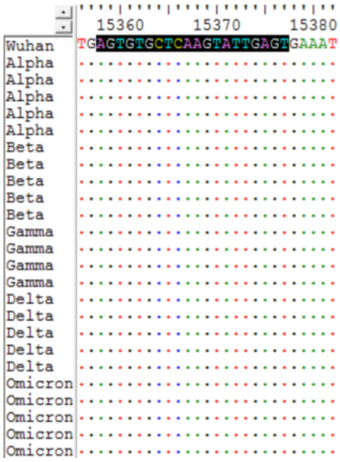

Title: SARS-CoV-2 Direct Detection Without RNA Isolation With Loop-Mediated Isothermal Amplification (LAMP) and CRISPR-Cas12

Target: N gene

Oligo: N-geneF3

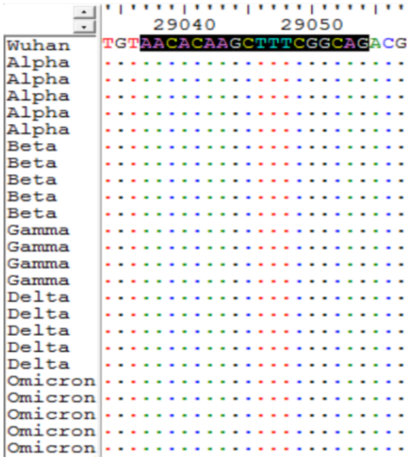

Target: N gene

Oligo: N-geneB3 (anti-sense)

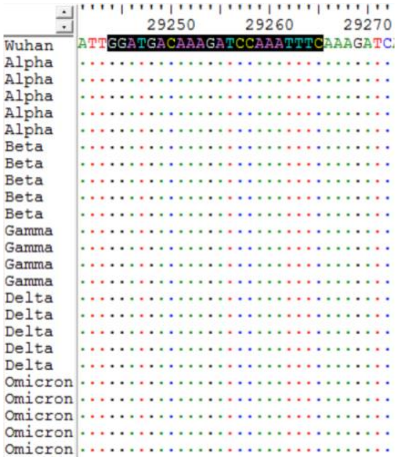

Target: N gene

Oligo: N-gene FIP

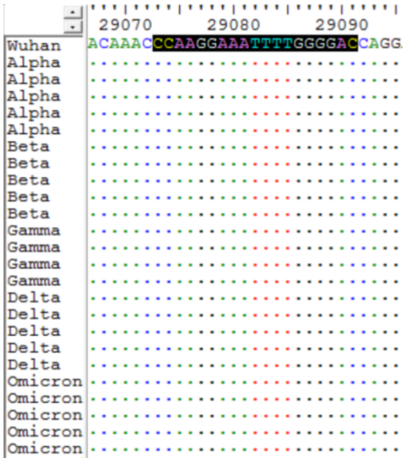

Target: N gene

Oligo: N-gene BIP (anti-sense)

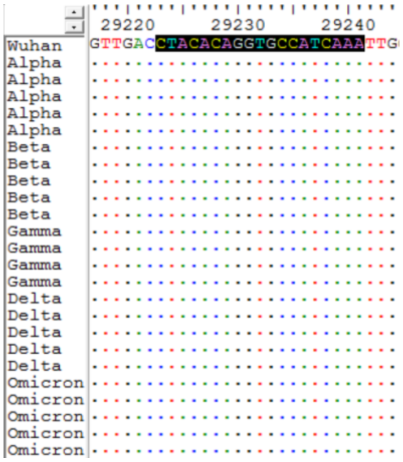

Target: N gene

Oligo: N gene FL (anti-sense)

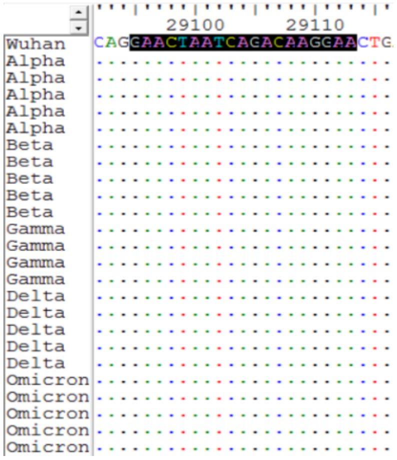

Target: N gene

Oligo: N gene BL

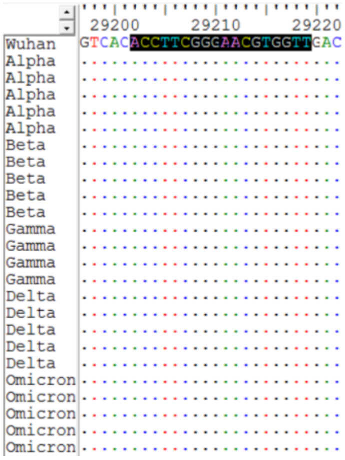

Target: N gene

Oligo: N gene gRNA

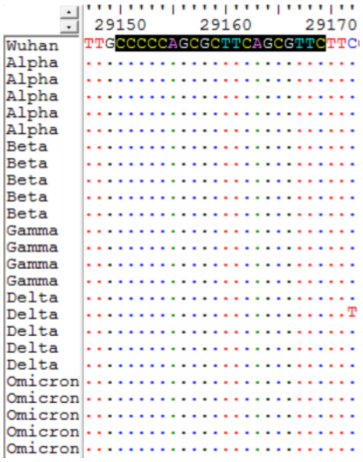

Title: SARS-CoV-2 RNA Detection by a Cellphone-Based Amplification-Free System with CRISPR/CAS-Dependent Enzymatic (CASCADE) Assay

Target: Orf1ab

Oligo: Orf1ab forward primer

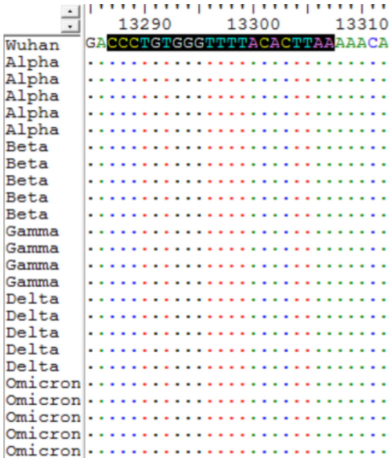

Target: Orf1ab

Oligo: Orf1ab reverse primer (anti-sense)

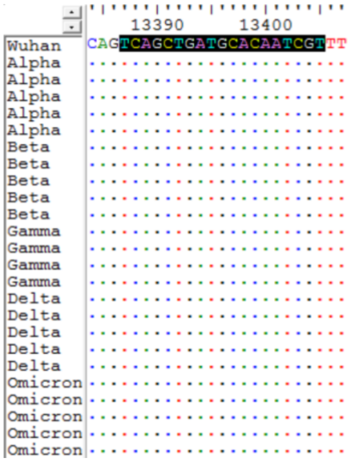

Target: Orf1ab

Oligo: Orf1ab crRNA (anti-sense)

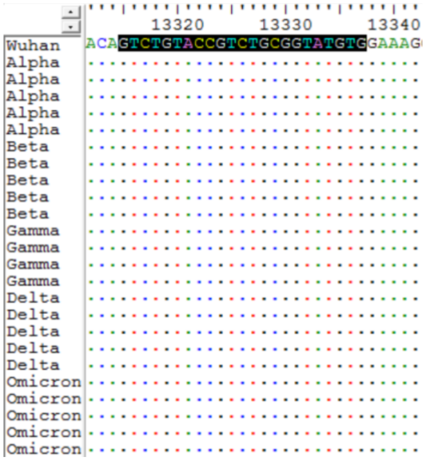

Title: Sensitive and Easy-Read CRISPR Strip for COVID-19 Rapid Point-of-Care Testing

Target: N gene
Oligo: nCoVnp-F1

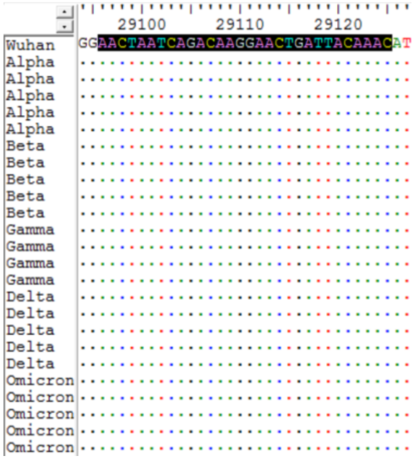

Target: N gene
Oligo: nCoVnp-R1

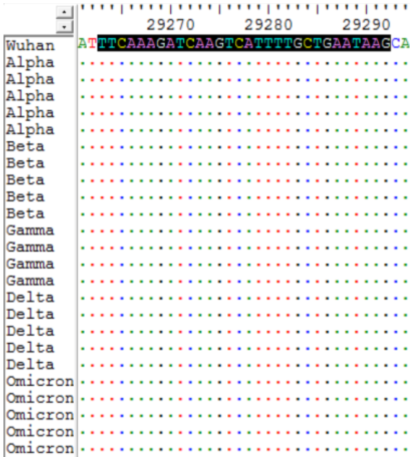

Target: N gene
Oligo: crRNA

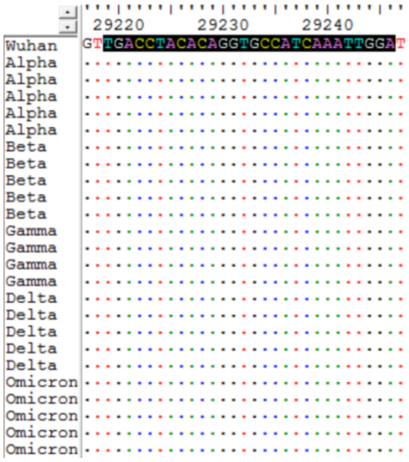

Title: Sensitive and rapid on-site detection of SARS-CoV-2 using a gold nanoparticle-based high-throughput platform coupled with CRISPR/Cas12-assisted RT-LAMP

Target: N gene

Oligo: N-gene-F3

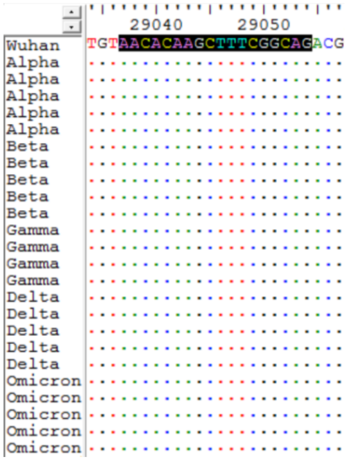

Target: N gene

Oligo: N-gene-B3 (anti-sense)

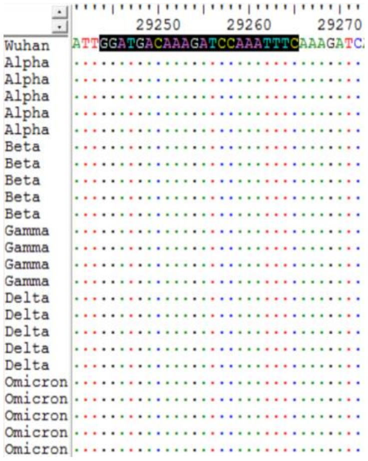

Target: N gene

Oligo: N-gene-FIP

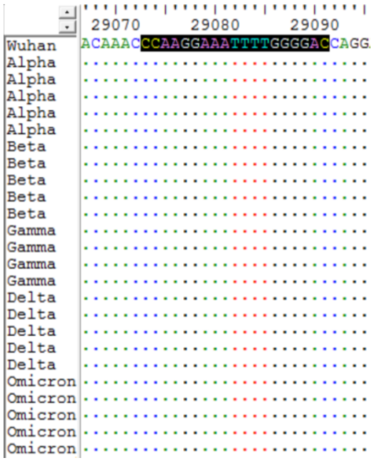

Target: N gene

Oligo: N-gene-BIP (anti-sense)

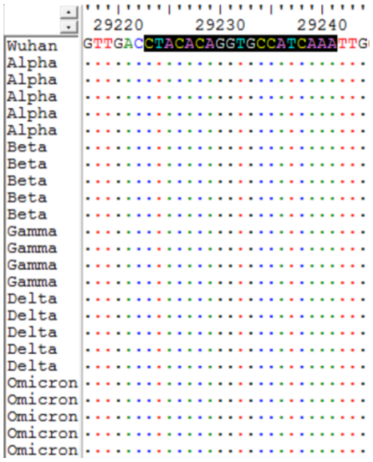

Target: N gene

Oligo: N gene-FL (anti-sense)

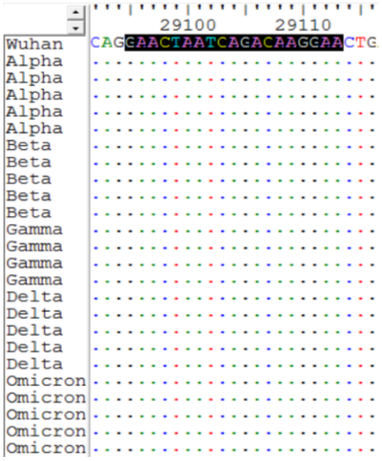

Target: N gene

Oligo: N gene-BL

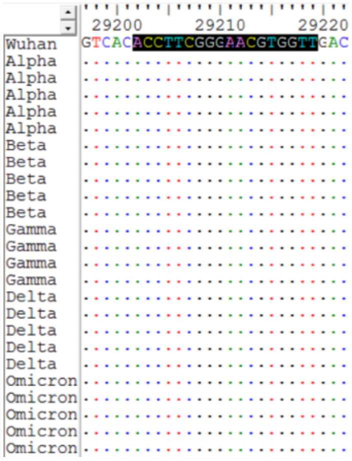

Target: N gene

Oligo: gRNA

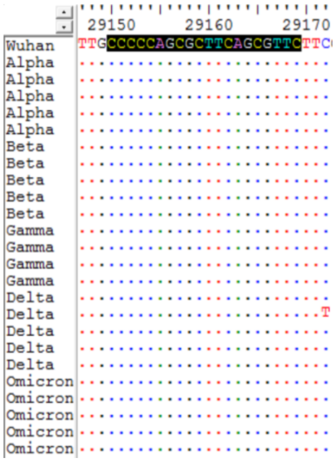

Title: Sensitive tracking of circulating viral RNA through all stages of SARS-CoV-2 infection

Target: Orf1ab

Oligo: ORF1ab-F

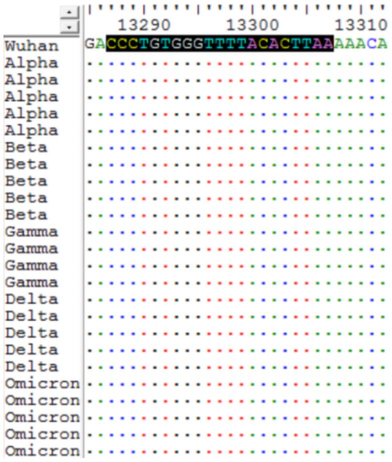

Target: Orf1ab

Oligo: ORF1ab-R (anti-sense)

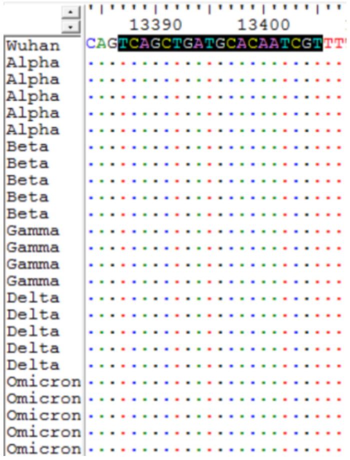

Target: Orf1ab

Oligo: gRNA -ORF1ab (anti-sense)

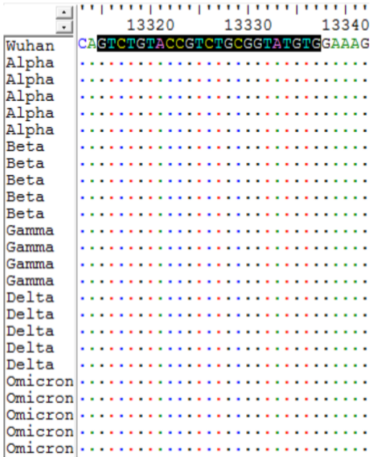

Title: Streamlined inactivation, amplification, and Cas13-based detection of SARS-CoV-2

Target: Orf1ab
Oligo: RPA Primer (forward)

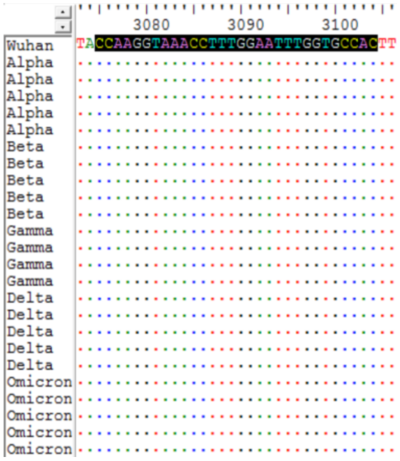

Target: Orf1ab
Oligo: RPA Primer (reverse)

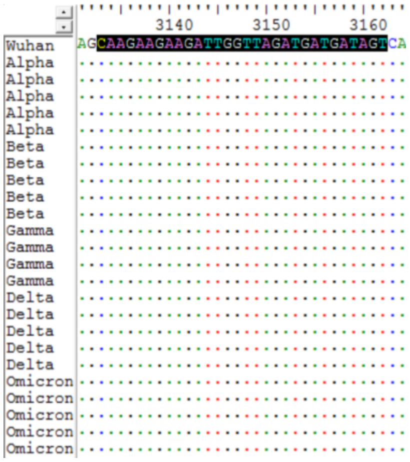

Target: Orf1ab
Oligo: Cas13a crRNA (anti-sense)

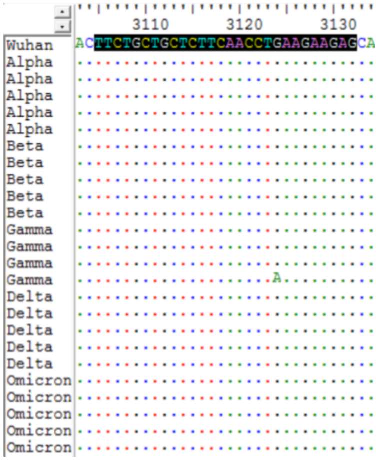

Title: Ultra-sensitive and high-throughput CRISPR-powered COVID-19 diagnosis

Target: Orf1ab
Oligo: ORF1ab-F

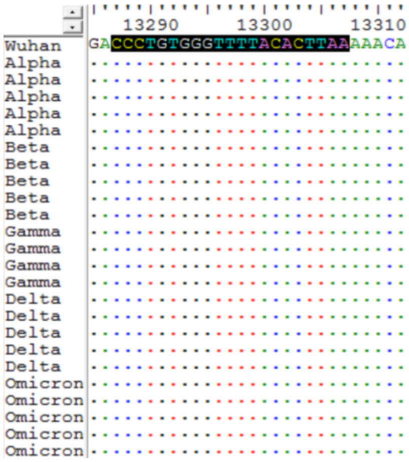

Target: Orf1ab
Oligo: ORF1ab-R (anti-sense)

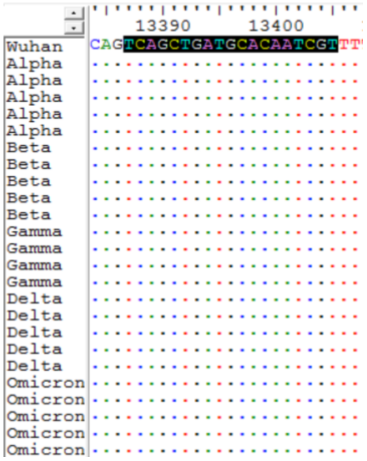

Target: N gene
Oligo: N-F

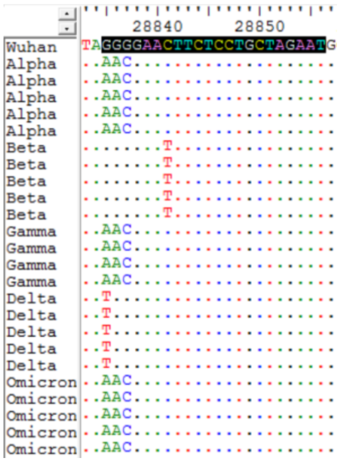

Target: N gene
Oligo: N-R (reverse)

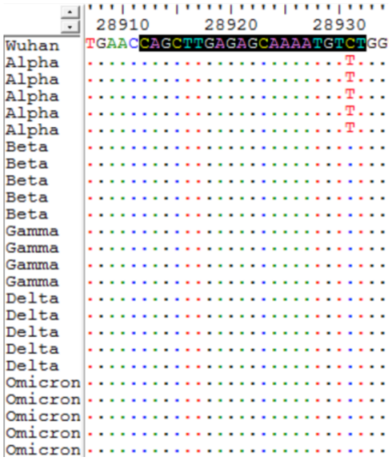

Target: Orf1ab
Oligo: gRNA -ORF1ab (anti-sense)

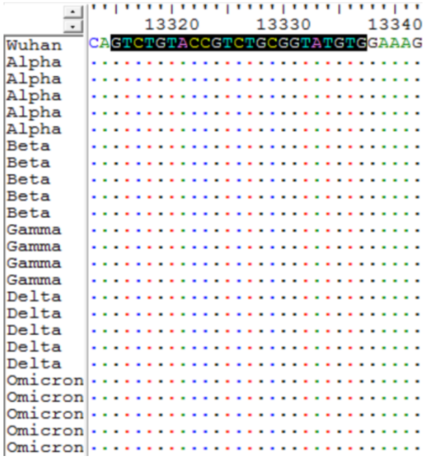

Target: N gene
Oligo: gRNA - N

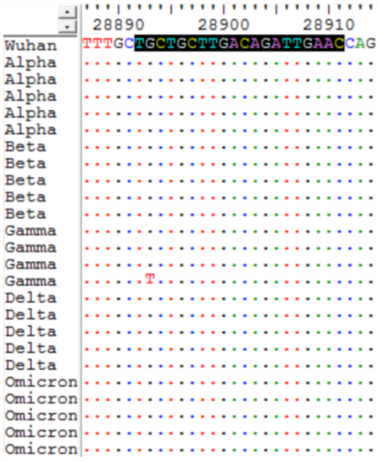

Title: Ultrasensitive and visual detection of SARS-CoV-2 using all-in-one dual CRISPR-Cas12a assay

Target: N gene

Oligo: Forward primer

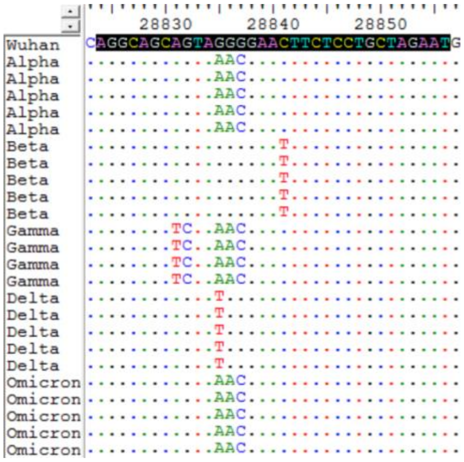

Target: N gene

Oligo: Reverse primer (anti-sense)

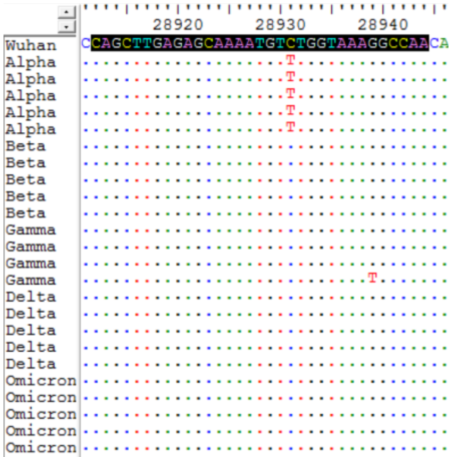

Target: N gene

Oligo: crRNA1 (anti-sense)

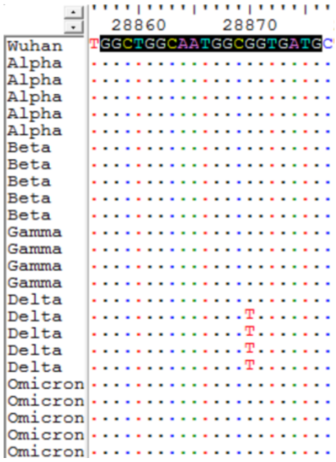

Target: N gene

Oligo: crRNA2

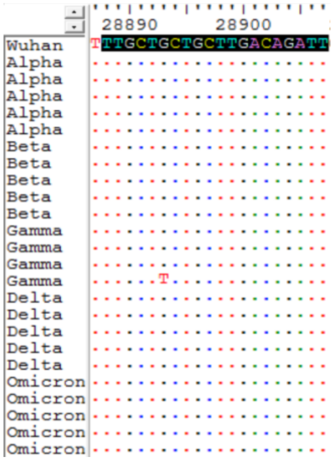

Target: N gene

Oligo: crRNA3

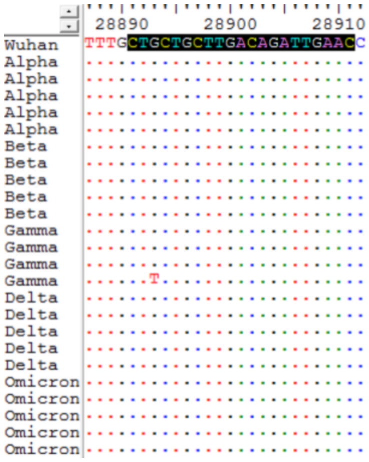

Title: UnCovid: A versatile, low-cost, and open-source protocol for SARS-CoV-2 RNA detection

Target: Orf1ab

Oligo: FCb3

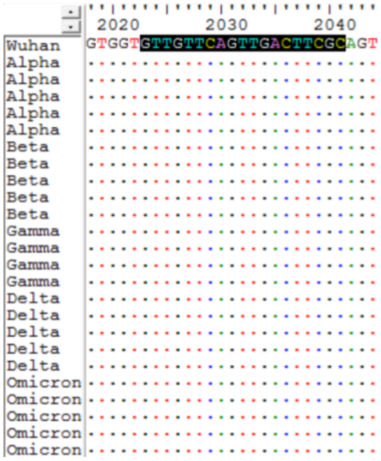

Target: Orf1ab

Oligo: RCb2 (anti-sense)

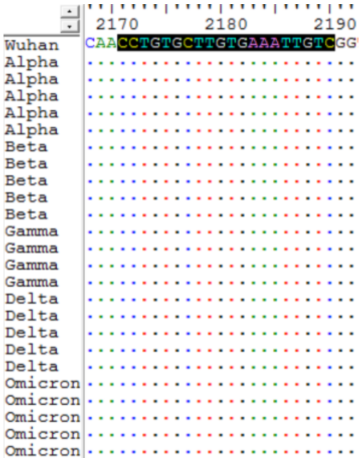

Target: N gene

Oligo: FN1

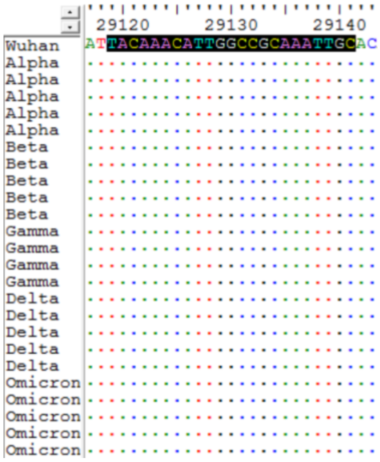

Target: N gene

Oligo: RN2 (anti-sense)

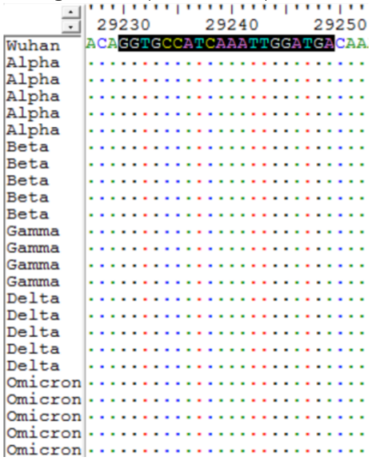

Target: Orf1ab

Oligo: ORF1ab crRNA

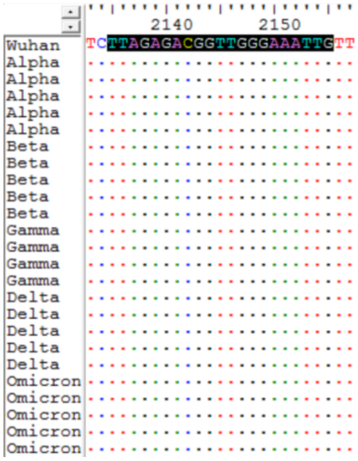

Target: N gene

Oligo: N crRNA

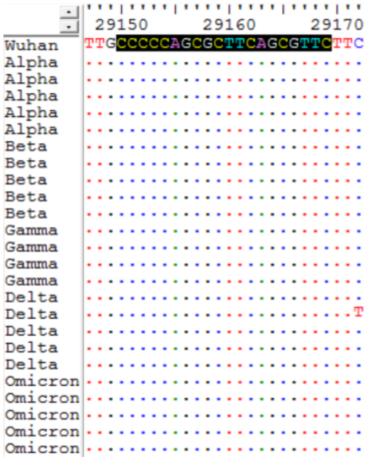

Title: Universally Stable and Precise CRISPR-LAMP Detection Platform for Precise Multiple Respiratory Tract Virus Diagnosis Including Mutant SARS-CoV-2 Spike N501Y

Target: N gene

Oligo: F3

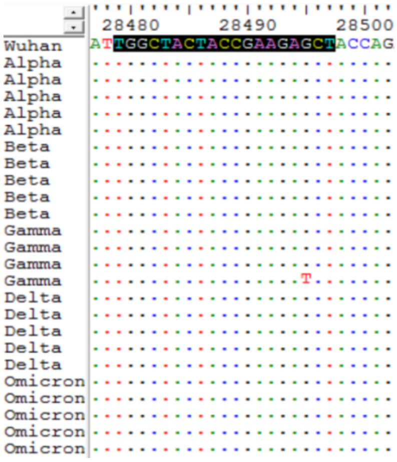

Target: N gene

Oligo: B3 (anti-sense)

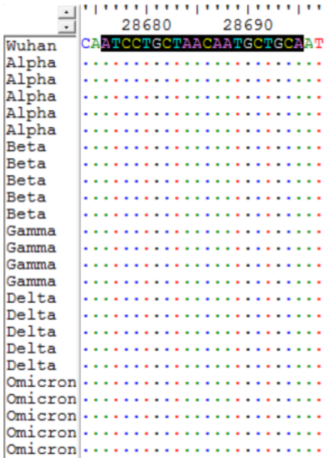

Target: N gene

Oligo: FIP

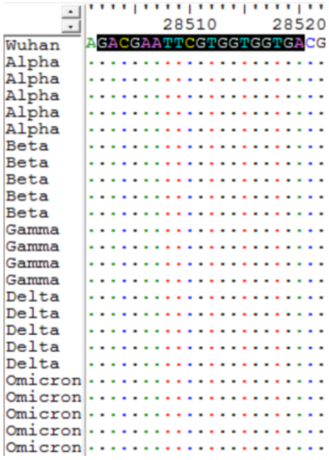

Target: N gene

Oligo: BIP (anti-sense)

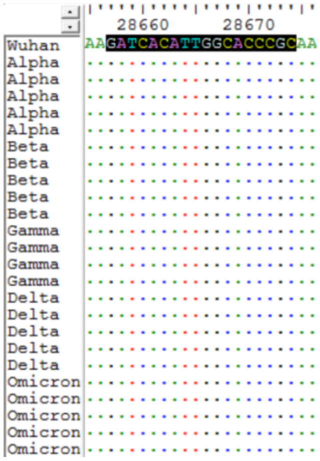

Target: N gene

Oligo: LF (anti-sense)

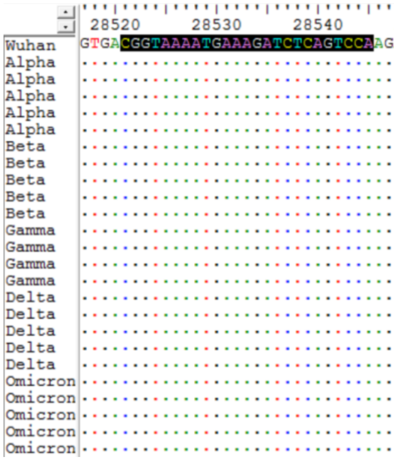

Target: N gene

Oligo: LB

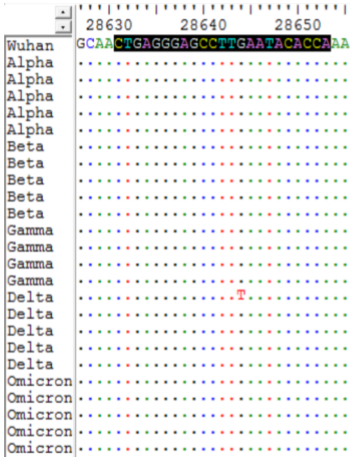

Target: S gene

Oligo: F3

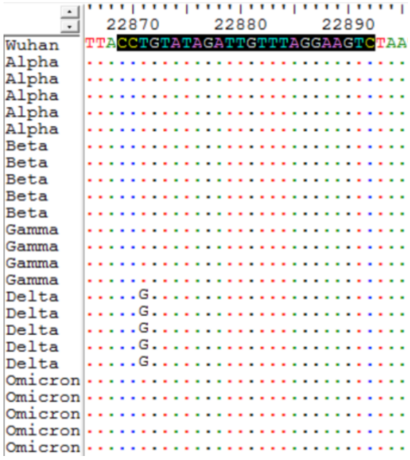

Target: S gene

Oligo: B3 (anti-sense)

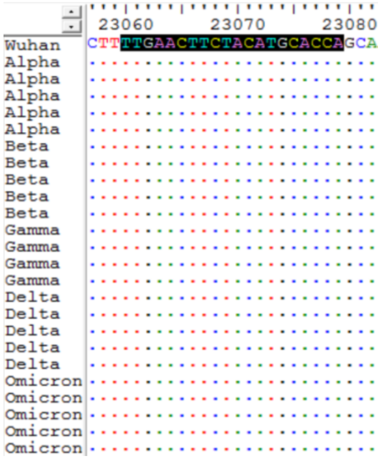

Target: S gene

Oligo: FIP

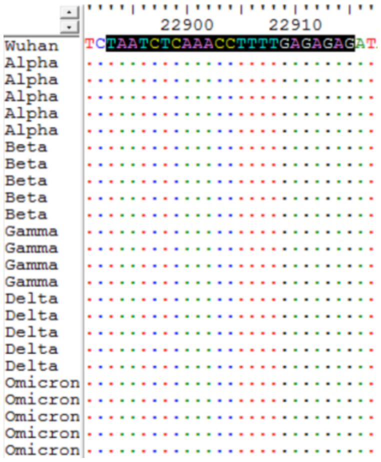

Target: S gene

Oligo: BIP (anti-sense)

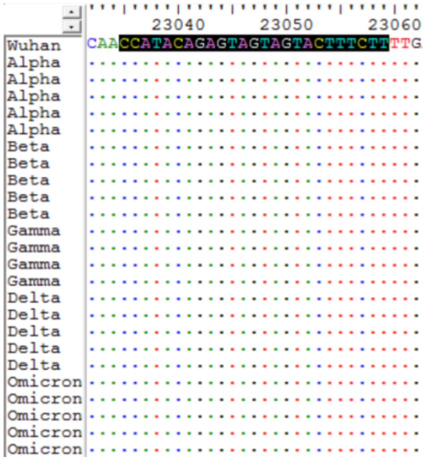

Target: S gene

Oligo: LF (anti-sense)

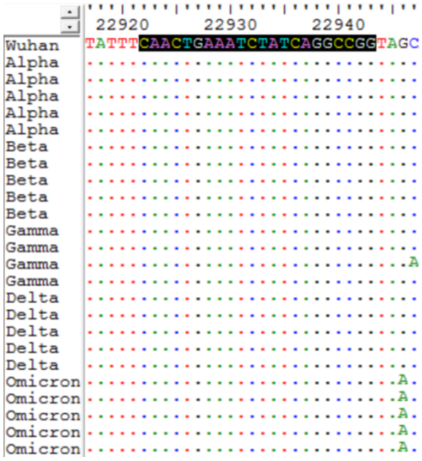

Target: RdRp gene

Oligo: F3

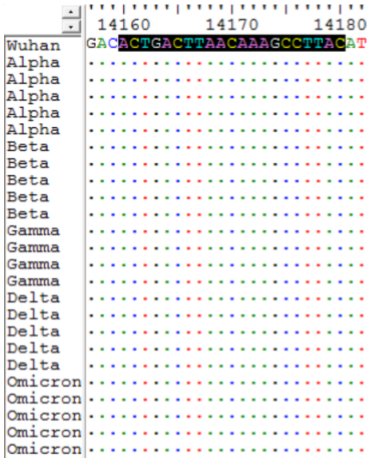

Target: RdRp gene

Oligo: B3 (anti-sense)

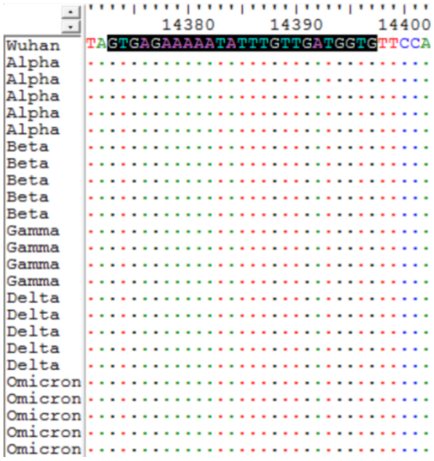

Target: RdRp gene

Oligo: FIP

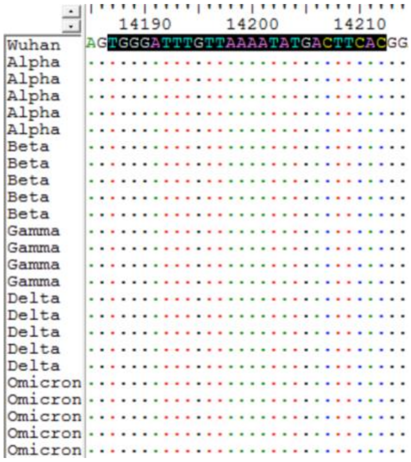

Target: RdRp gene

Oligo: BIP (anti-sense)

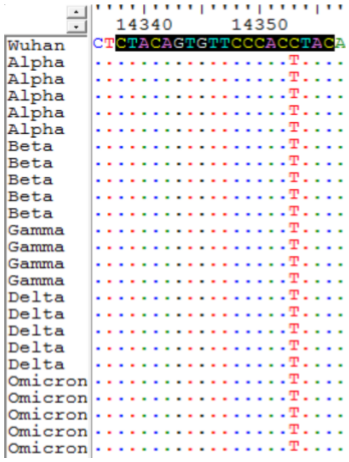

Target: RdRp gene

Oligo: LF (anti-sense)

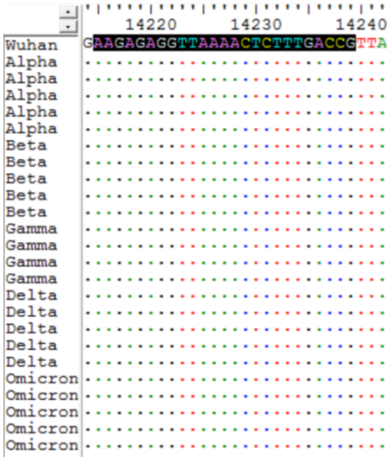

Target: RdRp gene

Oligo: LB

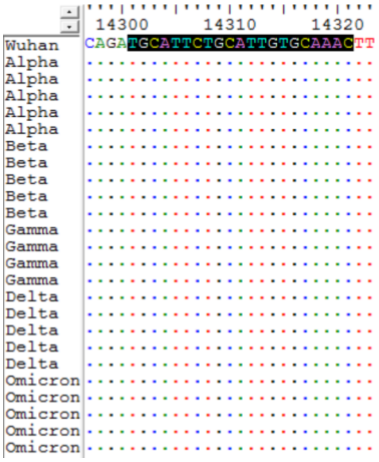

Target: N gene
Oligo: N-crRNA1

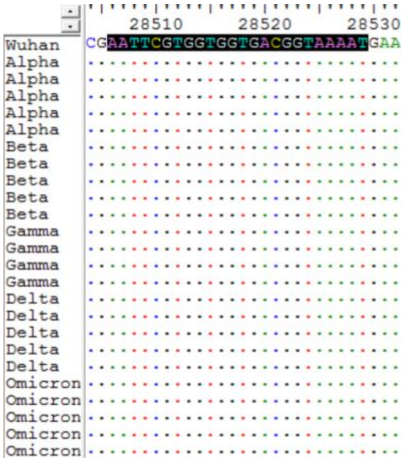

Target: S gene
Oligo: crRNA for S gene

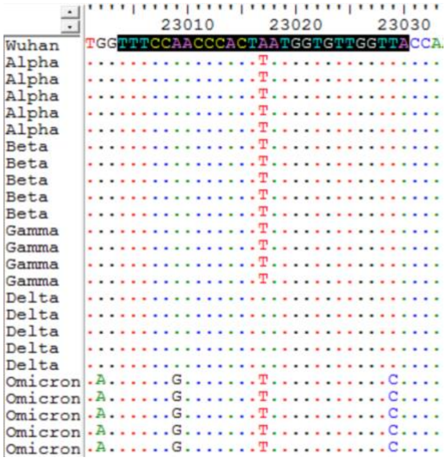

Target: RdRp gene
OligoRdRp-crRNA4 (anti-sense)

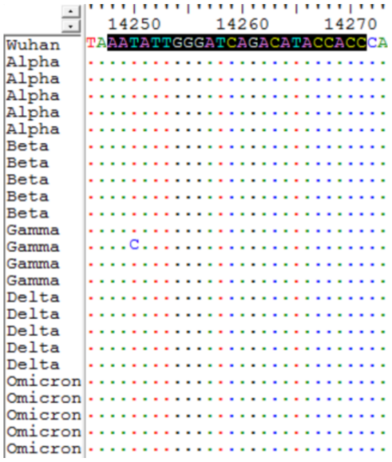

Title: Unlocking SARS-CoV-2 detection in low- and middle-income countries

Target: N gene

Oligo: Fn1

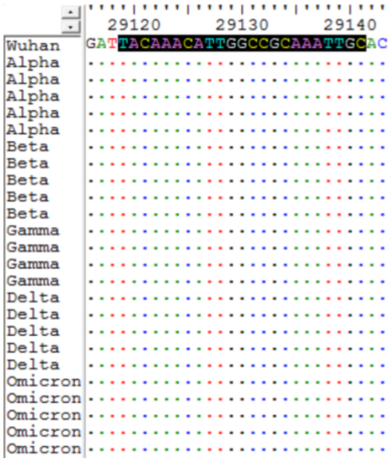

Target: N gene

Oligo: Fn2

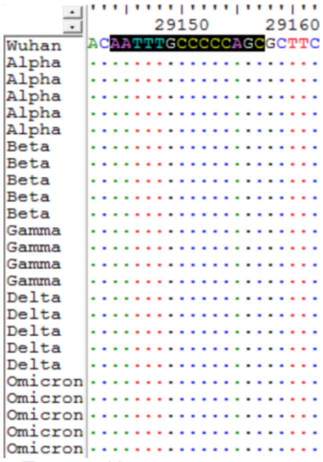

Target: N gene

Oligo: Fn3

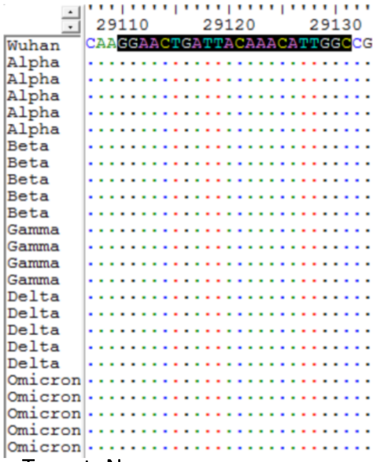

Target: N gene

Oligo: Rn1 (anti-sense)

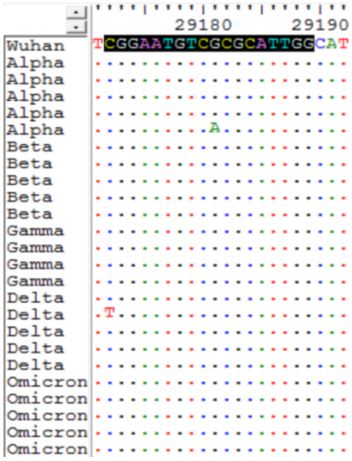

Target: N gene

Oligo: Rn2 (anti-sense)

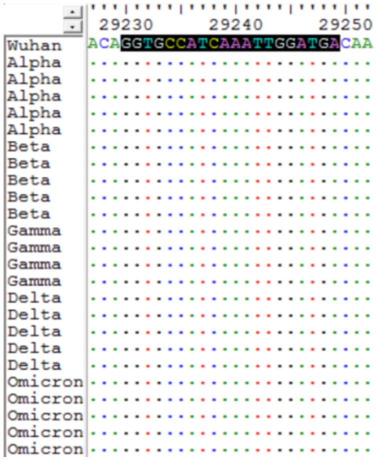

Target: ORF1ab

Oligo: Fcb1

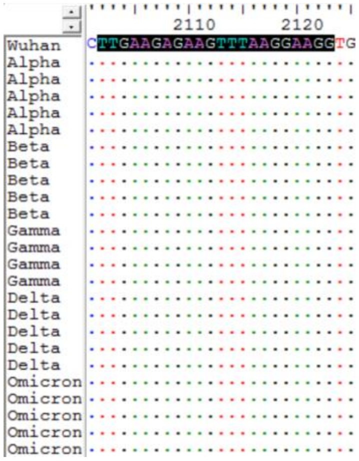

Target: ORF1ab

Oligo: Fcb2

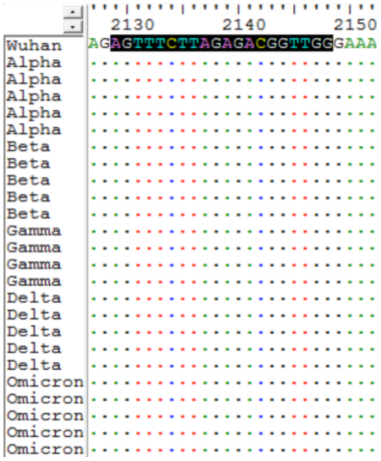

Target: ORF1ab

Oligo: Fcb3

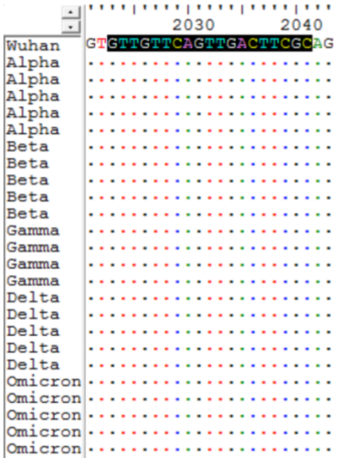

Target: ORF1ab

Oligo: Rcb1 (anti-sense)

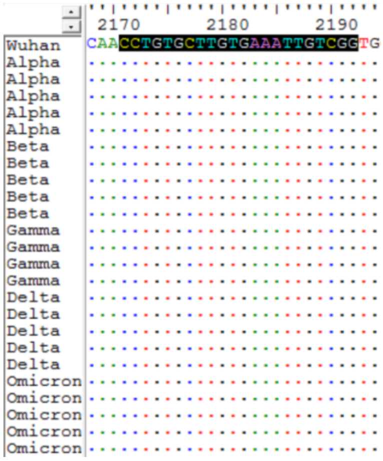

Target: ORF1ab

Oligo: Rcb2 (anti-sense)

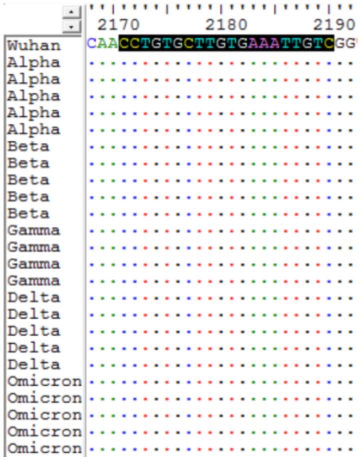

Target: N gene

Oligo: N_crRNA

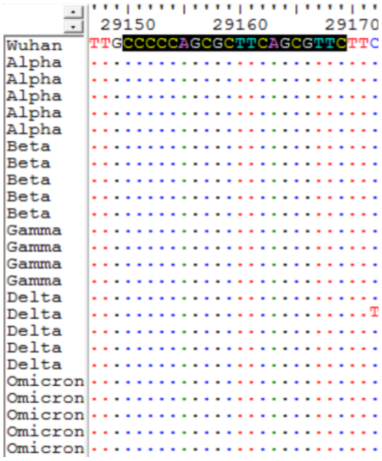

Target: ORF1ab

Oligo: ORF1ab_crRNA

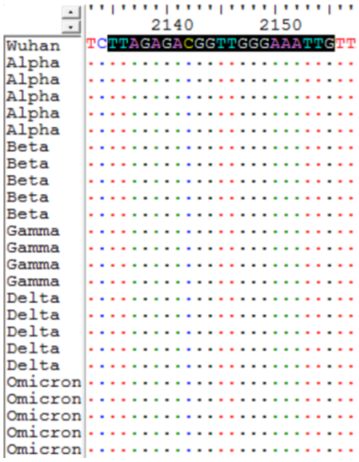

Supplement: Supplementary file 1 [file pathogens-11-00968-s001.zip › Figure S1-Multiple sequence alignments of SARS-CoV-2 VOC.pdf]
